# Synthesis of α-indolylacrylates as potential anticancer agents using a Brønsted acid ionic liquid catalyst and the butyl acetate solvent†

**DOI:** 10.1039/d0ra00990c

**Published:** 2020-04-02

**Authors:** Ahmed El-Harairy, Mennatallah Shaheen, Jun Li, Yuzhou Wu, Minghao Li, Yanlong Gu

**Affiliations:** Environmental, Energy and Green Chemistry Laboratory, Faculty of Agriculture, Damietta University 34511 Damietta Egypt ahmed.elharairy@hotmail.com; School of Chemistry and Chemical Engineering, Huazhong University of Science and Technology 430074 Wuhan China; Department of Pharmaceutical Chemistry, Faculty of Pharmacy, Horus University 34511 New Damietta Damietta Egypt; State Key Laboratory for Oxo Synthesis and Selective Oxidation, Lanzhou Institute of Chemical Physics Lanzhou 730000 China

## Abstract

In this study, new α-indolylacrylate derivatives were synthesized by the reaction of 2-substituted indoles with various pyruvates using a Brønsted acid ionic liquid catalyst in butyl acetate solvent. This is the first report on the application of pyruvate compounds for the synthesis of indolylacrylates. The acrylate derivatives could be obtained in good to excellent yields. A preliminary biological evaluation revealed their promising anticancer activity (IC_50_ = 9.73 μM for the compound 4l) and indicated that both the indole core and the acrylate moieties are promising for the development of novel anticancer drugs. The Lipinski's rule and Veber's parameters were assessed for the newly synthesized derivatives.

## Abstract 2

4lNew α-indolylacrylate derivatives were synthesized by reaction of 2-substituted indoles with various pyruvates using a Brønsted acid ionic liquid catalyst in butyl acetate solvent. This is the first application of pyruvate compounds for the synthesis of indolylacrylates.

## Graphical abstract

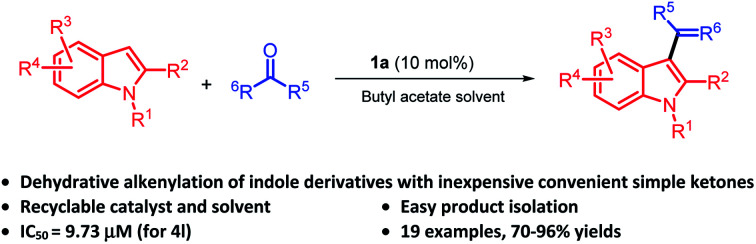

## Introduction

**Fig. 1:**
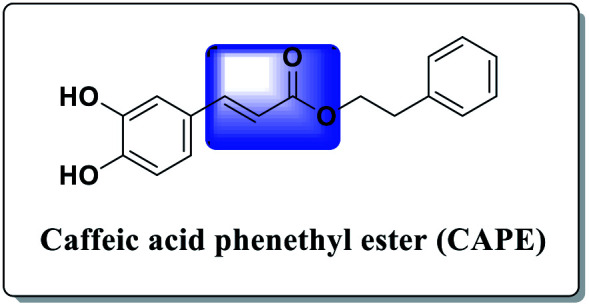
Chemical structure of caffeic acid phenethyl ester (CAPE).

The chemistry of indoles is an extensive research topic in organic synthesis because indole derivatives have unique biological activities.^1^ In the diverse chemical libraries of reported potent antitumor agents, several indole-based derivatives have been described as effective antitumor agents.^2^ Moreover, acrylate-containing derivatives have been proven to display prominent antitumor potency.^3^ Caffeic acid phenethyl ester (CAPE) (Fig. 1) is the main constituent of propolis, a resinous substance used in folk medicine for treating various ailments. CAPE has been widely reported to possess antitumor activities.^2*a*^

**Fig. 2:**
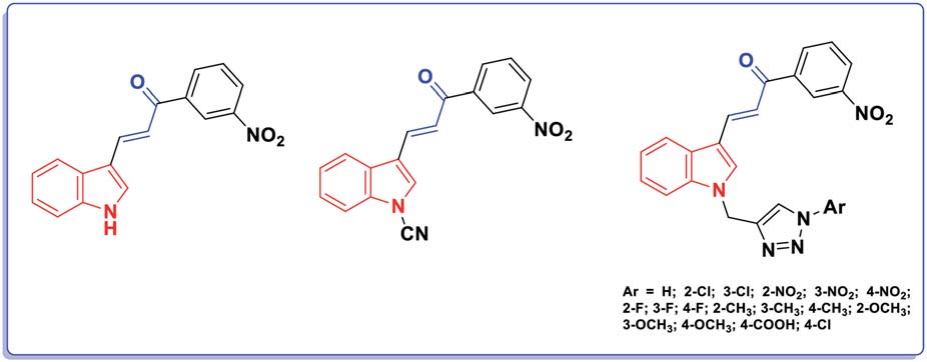
The reported indole acrylate-based anticancer agents.

Herein, our rational design is based on molecular hybridization. Molecular hybridization is a useful approach to design new biologically active agents. It involves the combination of two or more pharmacophoric entities with relevant biological activities to attain new hybrids with improved activity, selectivity, and safety.^4^ In addition, two structural motifs were considered in the design of the proposed scaffold: the indole moiety and the acrylate structure. Although the acrylic linkage has significant potency, a literature survey revealed that only few studies have been reported on the synthesis of α-indolylacrylate analogs^5^ and their biological activity as promising anticancer agents was evaluated in 2020 for the first time (Fig. 2).^6^

Some methods, such as a straightforward strategy involving the use of a Michael acceptor for the synthesis of α,β-unsubstituted indoles, have been developed to transform C–H bonds into C–C bonds by Friedel–Crafts alkylation and acylation reactions,^7^ also called cross-dehydrogenative coupling (CDC) reactions.^8^

**Scheme 1:**
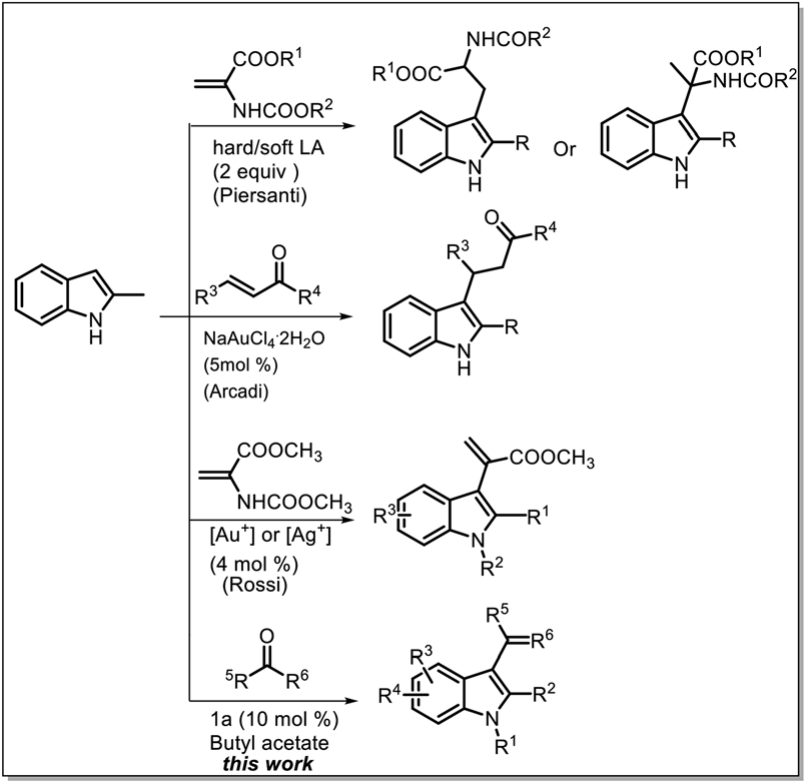
Routes for the synthesis of C3-functionalized indoles.

Most of the widely used methods involve the conjugate addition of heterocycles to α,β-unsaturated carbonyl compounds; however, most of these methods require different catalysts such as chiral transition-metal catalysts and organocatalysts.^9^ Moreover, in another reported study, directing groups or auxiliary amine catalysts were used to convert the β-position to the α-position through dehydrogenation at the β-position; however, studies based on this strategy are rare.^10^ Recently, some studies have reported the use of acrylate derivatives for the synthesis of indolylacrylates under harsh conditions such as the use of expensive catalysts, toxic solvents, and high temperatures (Scheme 1).^11^ Recently, we proposed the use of a sulfone-containing imidazolium-based Brønsted acid ionic liquid as a catalyst and butyl acetate as a solvent to replace dipolar and aprotic solvents.^12^ In this study, we investigated the possibility of using reactive carbonyl compounds, *i.e.* pyruvates, for the synthesis of α-indolylacrylate derivatives under green conditions, and to our delight, highest reactivity of pyruvate with indole was observed in a biphasic system with an ionic liquid as a catalyst and butyl acetate as a solvent.

## Results and discussion

We used our system 1a/butyl acetate^12^ in a simple protocol involving the reaction of 2-methyl indole with ethyl pyruvate as a model reaction (Table 1); this is the first report on the application of pyruvate compounds for the synthesis of α-indolylacrylate derivatives and replacement of dipolar, aprotic, and hazardous solvents by butyl acetate.

**Table 1:** The reaction of 2a with 3a under different conditions

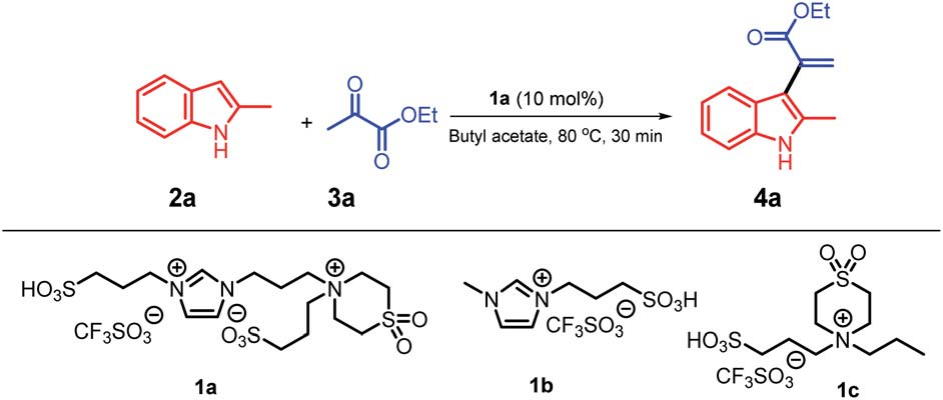			
Entry	Catalyst	Solvent	Yield (%)
1^a^	1a	Butyl acetate	91
2	1b	Butyl acetate	25
3	1c	Butyl acetate	65
4	1a	Ethyl acetate	70
5	TfOH	Butyl acetate	50
6	PTSA	Butyl acetate	47
7	TfOH	EtOH	35
8	TfOH	Anisole	40
9	TfOH	Nitromethane	10
10	TfOH	1,2-Dichloroethane	30
11	TfOH	1,4-Dioxane	7
12	TfOH	Toluene	70
13	TfOH	Benzene	65
14^b^	1a (15%)	Butyl acetate	91
15^c^	1a (5%)	Butyl acetate	72

a Reaction conditions: 2a, 0.3 mmol; 3a, 0.3 mmol; 4a, 0.3 mmol; catalyst, 0.03 mmol; medium, 0.5 ml; temperature, 80 °C; and time, 2 h.

b Temperature, 60 °C.

c Time, 1 h.

The reaction of 2-methylindole 2a with ethyl pyruvate 3a was conducted in a biphasic system at 80 °C for 30 minutes in the presence of 10 mol% of ionic liquid 1a as a catalyst and butyl acetate as a solvent; the ratio of 2a/3a was 1 : 1, and the product 4a was obtained in an excellent yield (91%) (Table 1, entry 1). With the Forbes's ionic liquid 1b,^12^4a was obtained in a 25% yield (entry 2), whereas with the ionic liquid 1c,^12^ the yield of 4a was increased to 65% (entry 3). Note that when we replaced the butyl acetate solvent with the ethyl acetate solvent, the yield of 4a reached 70% (entry 4). Strong acids, such as triflic acid and PTSA, were used with the butyl acetate solvent, and 4a was obtained in 50% and 47% yields (entries 5 and 6), respectively. We examined other green solvents such as ethanol and anisole, and 4a could be isolated in 35% and 40% yields, respectively. Nitromethane was tested as a polar solvent, and 4a was obtained in a 10% yield; moreover, when other dipolar and aprotic solvents, such as 1,2-dichloroethane (DCE) and 1,4-dioxane, were used, the yields of 4a were 30% and 7% (entries 10 and 11), respectively. These results indicate that the abovementioned reaction does not require dipolar and aprotic solvents, and the product 4a can be obtained in an excellent yield using weakly-to-moderately polar butyl acetate. Moreover, we investigated the reactivity of the substrate using non-polar solvents such as toluene and benzene, and the yields of 4a were significantly increased to 70 and 65% (entry 12 and 13), respectively. In addition, we increased the amount of the ionic liquid catalyst 1a; however, no further increase in the yield was observed (entry 14). On the other hand, when we decreased the amount of the ionic liquid catalyst 1a to 5 mol%, 4a was obtained in a 72% yield (entry 15). Moreover, the results of this reaction completely confirmed that a balance between butyl acetate as a solvent and ionic liquid 1a as a catalyst was required and the substrates were more active in this biphasic system. Moreover, the recyclability of the ionic liquid 1a was studied for conducting this model reaction on a large scale using 10 mmol of the substrate to obtain the target product 4a in a 91% yield; the results indicated that 1a could be recycled 8 times without any significant loss in its activity, and the recovery of the butyl acetate solvent reached about 96%.

**Scheme 2:**
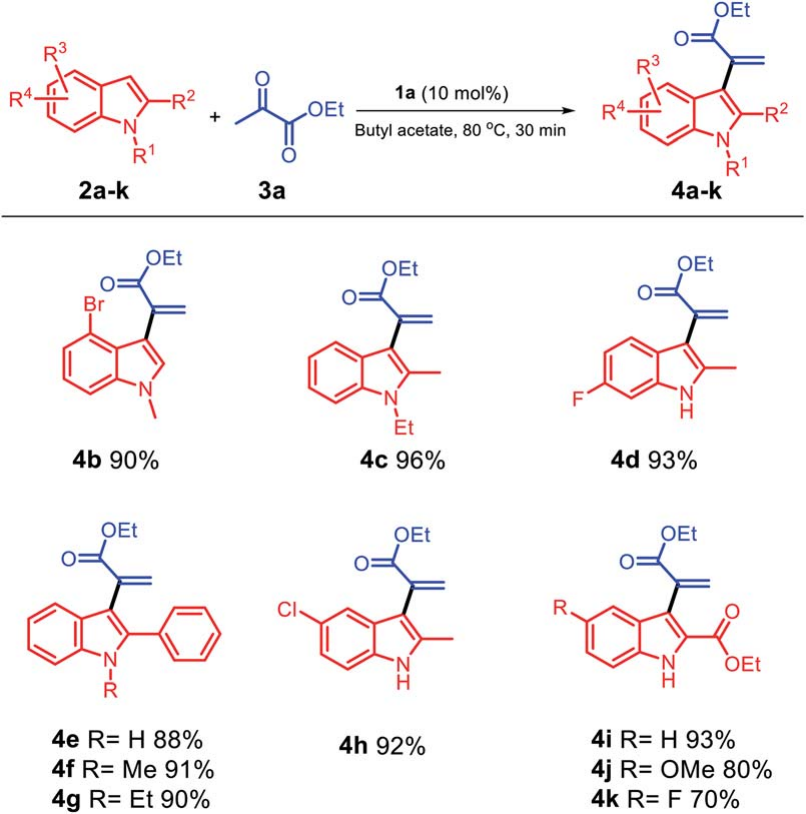
Scope of the substrates with respect to the indole component.

Based on these results, we selected the condition stated in entry 1 as an identical condition to extend the scope of the substrate by smoothly reacting different varieties of indole substituents with ethyl pyruvate 3a (Scheme 2); moreover, different positions of indole 2a–k were investigated for this reaction, and the corresponding α-indolylacrylates 4a–k with generally excellent to good yields were obtained. Both electron-rich (2a, 2c, and 2e–g) and moderately electron-poor (2b, 2d, and 2h) indoles readily participated in the reaction; however, when different positions of electron-poor indoles were used, the yield of 4k was affected and reached 70%. Moreover, when we changed the group at the C5 position to hydrogen and methoxy, 4i and 4j were obtained with increased yields, respectively. When electron-rich indoles were used, 4c and 4i were obtained in excellent yields (96% and 93%), respectively; using indoles with a phenyl group at the C2 position and different groups at the C1 position, the corresponding products 4e, 4f, and 4g could be obtained in 88%, 91%, and 90% yields, respectively. When the moderately electron-poor indoles 2b, 2d, and 2h with different groups were used, the corresponding products 4b, 4d, and 4h were obtained in very satisfactory yields (90%, 93%, and 92%), respectively, and no detectable formation of decomposition products occurred.

**Scheme 3:**
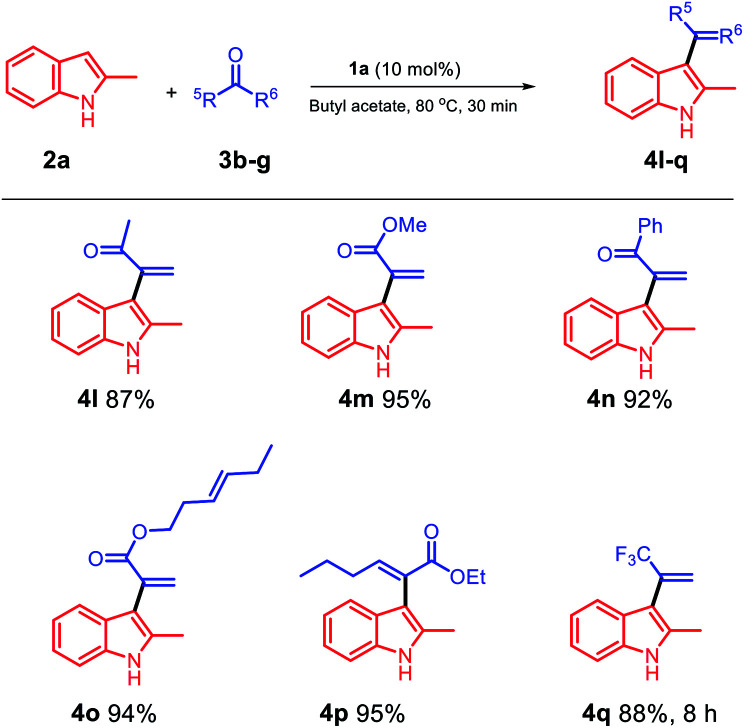
Scope of the substrates with respect to the ketone component.

Then, we probed the scope of the reaction with respect to the ketone component. The reaction of methyl pyruvate with methoxy pyruvate was also investigated, and the products 4l and 4m were obtained in excellent yields (Scheme 3). Moreover, we used a pyruvate substrate with a phenyl group at the C5 position, and the obtained product 4n was isolated with a 92% yield. In addition, *trans*-3-hexenyl pyruvate (3e) smoothly reacted with 2-methylindole using butyl acetate as a solvent and 1a as a catalyst, and the *trans*-C=C bond was effectively retained in the product 4o isolated with an excellent yield of 94%. Ethyl 2-oxohexanoate was examined in the biphasic system of 1a/butyl acetate, and 4p was obtained in a 95% yield. We used trifluoroacetone to see if it would react in the 1a/butyl acetate biphasic system, and to our delight, the target product 4q was obtained in an 88% yield after 8 h.

**Scheme 4:**
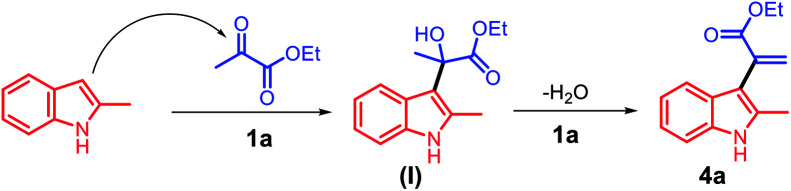
The proposed reaction mechanism for the synthesis of α-indolylacrylates.

Based on the abovementioned results, a plausible mechanism was proposed (Scheme 4). The C3 position of 2-methyl indole is a reactive nucleophilic site and can attack the ketone carbonyl group of ethyl pyruvate. The initial episode of the reaction involved the formation of an intermediate hydroxyl group (I) from 2a and 3a in the presence of the acid catalyst 1a. The intermediate (I) can be possibly converted to 4a by eliminating one molecule of water.

**Scheme 5:**
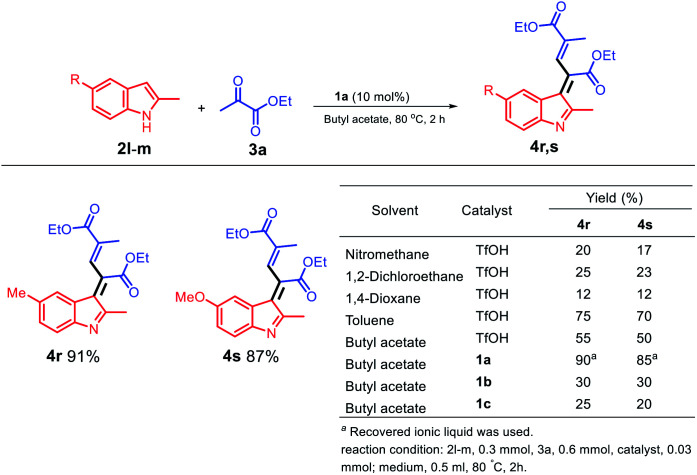
Schematic for the synthesis of electro-rich indolylacrylates.

When an electro-rich group, such as methyl and methoxy group, was used at the C3 position, different structures were obtained; moreover, two molecules of ethyl pyruvate were reacted with one molecule of a 2-methylindole derivative (Scheme 5) to optimize the conditions for the solvents and acid catalysis, and the final products 4r and 4s were obtained in excellent yields. However, when a dipolar solvent was used (Scheme 5), 4r and 4s were obtained in poor yields; moreover, using the non-polar solvent toluene, the yield of 4s was increased to 70%. As observed from the table shown in Scheme 5, the 1a/butyl acetate system is a unique system because it not only can replace the dipolar aprotic solvent but can also replace a non-polar toxic solvent such as toluene.

**Scheme 6:**
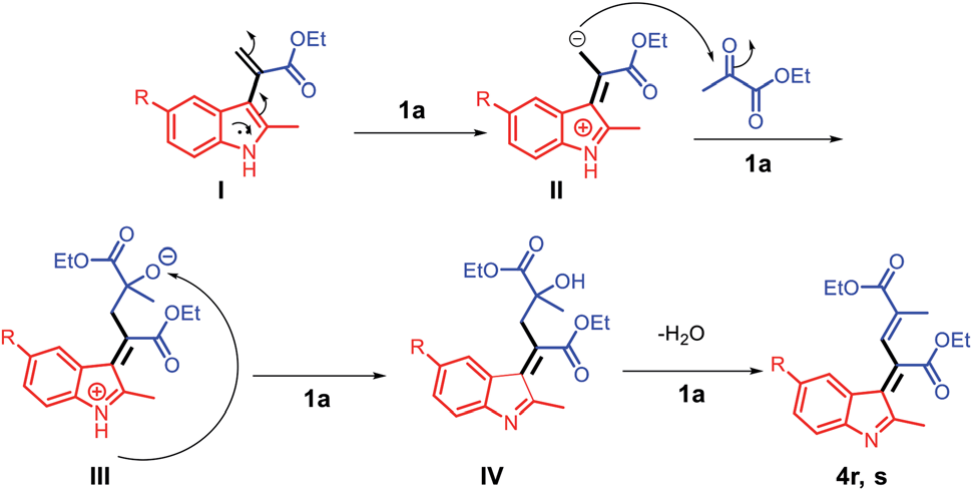
The proposed reaction mechanism for the synthesis of the compounds 4r and 4s.

Considering the formation of the two products 4r and 4s, the nucleophilicities of 2l and 2m in the presence of acid can be considered as the key for rendering the reaction possible. The C3 positions of 2l and 2m are reactive nucleophilic sites that can attack the electrophilic carbonyl group of 3a; using the acid ionic liquid catalyst 1a, the intermediate (I) can be converted to intermediate (II), which has a nucleophilic carbon and can attack the carbonyl group of another molecule of 3a and form the intermediate (III). The abstraction of the NH proton by the negatively charged oxygen using the Brønsted acid ionic liquid catalyst 1a leads to the formation of (IV), which undergoes water elimination to finally afford the products 4r and 4s (Scheme 6).

We studied the reaction of 2-unsubstituted indole with ethyl pyruvate, and the C3 position of the indole nucleus reacted with the α-carbon of ethyl pyruvate to form a new carbon–carbon bond. We optimized the conditions of this reaction using different solvents and catalysts; the ionic liquid was immiscible with the organic phase, thus forming a biphasic reaction system. The designed structure of the ionic liquid 1a involved a cyclic sulfone fragment, which enabled the ionic phase to provide a suitable reaction microenvironment for the stabilization of the reaction intermediate. Other ionic liquids, such as 1b and 1c, do not have this property due to the lack of a strongly polar part. Moreover, the biphasic system of 1a/butyl acetate is the best condition for this reaction (Table 2, entry 8).

**Table 2:** The behaviour of 2-unsubstituted indoles^a^

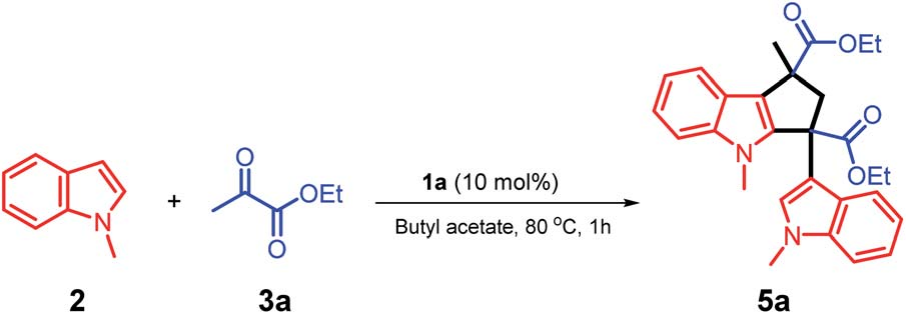			
Entry	Catalyst	Solvent	Yield
1	TfOH	1,4-Dioxane	10
2	TfOH	1,2-Dichloroethane	42
3	TfOH	MeNO_2_	20
4	TfOH	EtOH	38
5	TfOH	Anisole	15
6	TfOH	Ethyl acetate	35
7	TfOH	Butyl acetate	42
8	1a	Butyl acetate	92
9	1b	Butyl acetate	33
10	1c	Butyl acetate	63

a 2a, 0.3 mmol; 3a, 0.3 mmol, TfOH, 0.03 mmol; 1a, 0.03 mmol; solvent, 10 ml, 80 °C, 1 h.

**Scheme 7:**
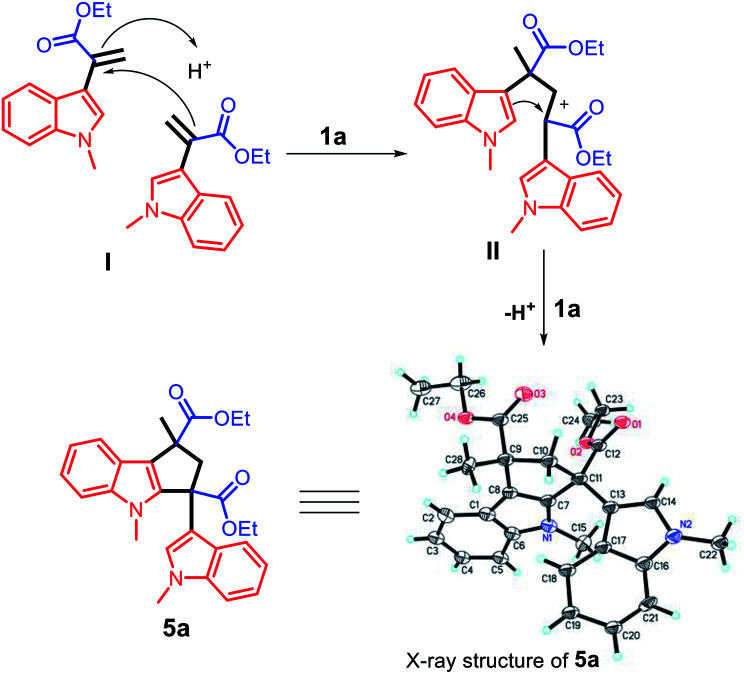
The proposed reaction mechanism for the synthesis of compound 5a.

The unique acidic environment offered by the ionic liquid catalyst 1a should be the key to initiate the dimerization of two molecules of intermediate (I) to form the diester indole intermediate (II); the C2 position of the indole ring will act as a nucleophilic site to attack the positively charged carbon of (II) to afford the product 5a (Scheme 7). The crystal and molecular structures of 5a were further confirmed using single-crystal X-ray diffraction (ESI†).

**Fig. 3:**
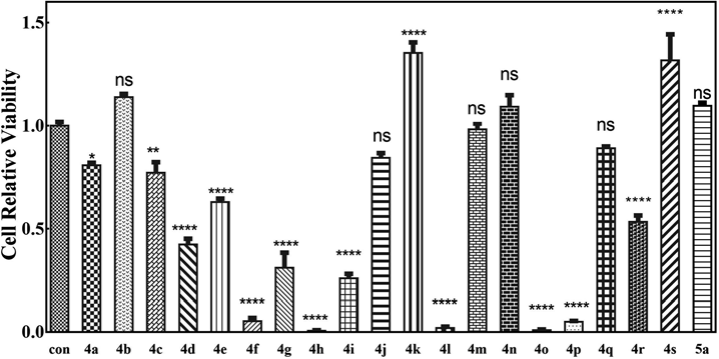
MTT assays of cell relative viability. (a) Herein, 100 μM 4a–s and 5a were incubated with the HeLa cells for 48 h. ^ns^ represents *P* > 0.05, * represents *p* value < 0.05, ** represents *p* value < 0.01, and **** represents *p* value < 0.001. The significant difference was compared with the control group.

**Fig. 4:**
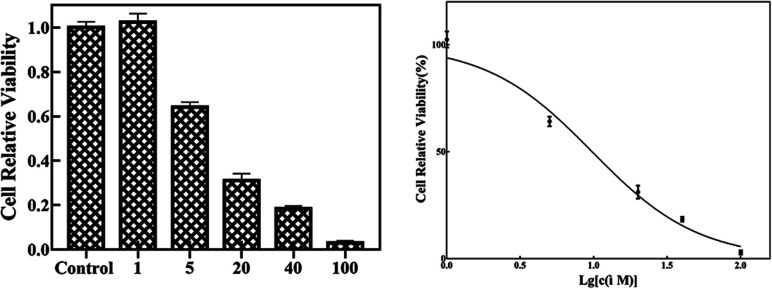
The compound 4l at different concentrations was incubated with the HeLa cells for 48 h. The IC_50_ is 9.73 μM.

Since many organic acrylates and indole-containing compounds have anticancer properties, the newly synthesized α-indolylacrylates 4a–s and 5a were screened for their *in vitro* growth inhibitory potential against the human cervical adenocarcinoma (HeLa) cell line using the 3-(4,5-dimethyldiazol-2-yl)-2,5-diphenyltetrazolium bromide (MTT) assay. The MTT cell proliferation assay has been widely accepted as a reliable way to measure the cell proliferation rate when metabolic events lead to apoptosis or necrosis. After incubation with the abovementioned compounds at different concentrations for 24 h, the cells were treated with MTT to measure their growth/viability using a spectrophotometer.^13^ The results showed that the synthesized compounds 4f, 4h, 4l, 4o, and 4p have maximum percentage cytotoxicity at 100 μg ml^−1^ (Fig. 3). These five compounds were subjected to further cytotoxicity studies against the HeLa cell line to determine the IC_50_ values. Among these derivatives, the compound 4I exhibited a potent anticancer activity with the IC_50_ value of 9.73 μg (Fig. 4). In future, several derivatives of 4l analogs will be subjected to further tests for cytotoxic activity against multiple human cancer cell lines and inhibitory activity against several enzymes to study a comprehensive SAR.

### *In silico* studies

#### Molinspiration calculations

The Lipinski's rule of five is a rule of thumb that is used to determine the drug likeness or investigate the properties that would make a chemical compound with a certain pharmacological or biological activity a likely orally active drug in humans.^14^ Similarly, the topological polar surface area (TPSA) and number of rotatable bonds (Nrotb) affect the extent of drug absorption.^15^ Molinspiration software^16^ is an important computational tool for the analysis of the Lipinski's rule parameters TPSA and Nrotb of new compounds. The results (Table 3) illustrated that all the investigated compounds are in agreement with the adequate criteria, *i.e.* adequate TPSA and Nrotb with zero violation of the Lipinski's rule (except for 5a with one violation), of Lipinski's rule, and these compounds are predicted to be well absorbed orally in humans.

**Table 3:** TPSA, Nrotb, and the calculated Lipinski's rule for the new compounds

Comp. no.	Molecular properties						
	TPSA^a^	Nrotb^b^	mi log *P*^c^	*n*OH–NH^d^	*n*O–N^e^	M. wt^f^	nVs^g^
4a	42.10	4	2.90	1	3	229.28	0
4b	31.24	4	3.51	0	3	308.18	0
4c	31.24	5	3.35	0	3	257.33	0
4d	42.10	4	3.04	1	3	247.27	0
4e	42.10	5	4.36	1	3	291.35	0
4f	31.24	5	4.42	0	3	305.38	0
4g	31.24	6	4.80	0	3	319.40	0
4h	42.10	4	3.56	1	3	263.72	0
4i	68.40	7	3.13	1	5	287.31	0
4j	77.64	8	3.16	1	6	317.34	0
4k	68.40	7	2.30	1	5	305.31	0
4l	32.86	2	2.21	1	2	199.25	0
4m	42.10	3	2.53	1	3	215.25	0
4n	32.86	3	3.81	1	2	261.32	0
4o	42.10	7	4.20	1	3	283.37	0
4p	42.10	6	4.45	1	3	271.36	0
4q	15.79	2	3.59	1	1	225.21	0
4r	65.50	7	4.35	0	5	341.41	0
4s	74.74	8	3.96	0	6	357.41	0
5a	62.48	7	5.45	0	6	458.56	1

a Topological polar surface area.

b Number of rotatable bonds.

c The parameter of lipophilicity.

d Number of hydrogen bond donor sites.

e Number of hydrogen bond acceptor sites.

f Molecular weight.

g Number of violations from the Lipinski's rule of five.

## Conclusions

Herein, using an ionic liquid as a catalyst and butyl acetate as a solvent, we successfully synthesized various α-indolylacrylate derivatives *via* the dehydrative alkenylation of indole derivatives with inexpensive, convenient, and simple ketones. Compared with the established method for the synthesis of *α*-indolylacrylates, the method proposed herein is a green approach involving a cost-effective reaction, high synthetic efficiency, recyclable catalyst, recyclable solvent, and easy product isolation. The newly synthesized compounds were evaluated for their *in vitro* anticancer activity against a human cervical adenocarcinoma (HeLa) cell line, and the results revealed that the compound 4l was the most active member with the IC_50_ value of 9.73 μM. Moreover, based on the results of the Molinspiration calculations, the compound 4l was predicted to have good oral absorption. Based on the abovementioned results, 4l is considered to be a promising lead for the future development of new potent antitumor agents.

## Experimental

### General remarks

1-(3-Aminopropyl)imidazole, divinyl sulfone, 1,3-propane sulfonate, trifluoromethanesulfonic acid, *n*-butylamine, 1-methylimidazole, 2-methyl-1*H*-indole, 1-methyl-1*H*-indole, 2-phenyl-1*H*-indole, 1-methyl-2-phenyl-1*H*-indole, 1-ethyl-2-phenyl-1*H*-indole, 4-bromo-1-methyl-1*H*-indole, ethyl 1-methyl-1*H*-indole-2-carboxylate, 1-ethyl-2-methyl-1*H*-indole, 6-fluoro-2-methyl-1*H*-indole, 5-methoxy-2-methyl-1*H*-indole, 2,5-dimethyl-1*H*-indole, ethyl 5-fluoro-1*H*-indole-2-carboxylate, ethyl 5-methoxy-1*H*-indole-2-carboxylate, and 5-chloro-2-methyl-1*H*-indole were purchased from Energy Chemical Company. Ethyl pyruvate, biacetyl, 1-phenylpropane-1,2-dione, methyl pyruvate, ethyl 2-oxohexanoate, (*E*)-hex-3-en-1-yl 2-oxopropanoate, and 1,1,1-trifluoropropan-2-one were purchased from Alfa Aesar Chemical Company. Butyl acetate, ethyl acetate, petroleum ether, sodium chloride, methanol, acetonitrile, acetone, 1,2-dichloroethane, anisole, 1,4-dioxane, and ethanol were purchased from Sinopharm Chemical Reagents Limited Company (SCRC). The ^1^H, ^13^C, and ^19^F NMR spectra were obtained using Bruker AV-400 (400 MHz ^1^H, 100 MHz ^13^C, and 375 MHz ^19^F) at room temperature. Fourier transform infrared (FTIR) spectra were acquired *via* FTIR Bruker (VERTEX 70) using the liquid film technology. A high-resolution mass spectrum (HRMS) was obtained using the Bruker micrOTOF-Q II instrument. Melting points of the products were determined by a microscopic melting point meter (Yu Hua Instrument, X-4).

### A typical procedure for the reaction of 2-methyl-1*H*-indole with ethyl pyruvate (the model reaction)

The reaction was carried out in a 10 ml V-type flask equipped with triangle magnetic stirring. In a typical reaction, 1a (19.11 mg, 0.03 mmol) was mixed with 2-methylindole 2a (39.354 mg, 0.3 mmol) and ethyl pyruvate 3a (34.857 mg, 0.3 mmol) in 0.5 ml of butyl acetate. The mixture was stirred for 30 min at 80 °C. After the completion of the reaction, the mixture was cooled down to room temperature. Butyl acetate was removed under reduced pressure and then extracted with EtOAc (3 × 0.5 ml). The product was obtained by isolation with preparative TLC (eluting solution: petroleum ether/ethyl acetate = 10/1 v/v). The desired product 4a was obtained in a 90–91% yield. At the end, the Brønsted acid ionic liquid catalyst was recycled several times; a sodium chloride solution (3 × 0.5 ml) was added to dissolve the ionic liquid; then, EtOAc (3 × 0.5 ml) was added to extract the remaining desired product. Tests for substrate scope were achieved according to the model reaction conditions.

### A typical procedure for the reaction of 2,5-dimethyl-1*H*-indole/5-methoxy-2-methyl-1*H*-indole with ethyl pyruvate

The reaction was carried out in a 10 ml V-type flask equipped with triangle magnetic stirring. In a typical reaction, 1a (19.11 mg, 0.03 mmol) was mixed with 2,5-dimethyl-1*H*-indole (43.561 mg, 0.3 mmol)/5-methoxy-2-methyl-1*H*-indole (48.361 mg, 0.3 mmol) and ethyl pyruvate 3a (34.857 mg, 0.3 mmol) in 0.5 ml of butyl acetate. The mixture was stirred for 2 h at 80 °C. After the completion of the reaction, the mixture was cooled down to room temperature. Butyl acetate was removed under reduced pressure and then extracted with EtOAc (3 × 0.5 ml). The product was obtained by isolation with preparative TLC (eluting solution: petroleum ether/ethyl acetate = 10/1 v/v). The desired products 5r and 5s were obtained in a 91% and 87% yield, respectively.

### A typical procedure for the reaction of 1-methyl-1*H*-indole with ethyl pyruvate

The reaction was carried out in a 10 ml V-type flask equipped with triangle magnetic stirring. In a typical reaction, 1a (19.11 mg, 0.03 mmol) was mixed with 1-methylindole (39.354 mg, 0.3 mmol) and ethyl pyruvate 3a (34.857 mg, 0.3 mmol) in 1 ml of butyl acetate. The mixture was stirred for 1 h at 80 °C. After the completion of the reaction, the mixture was cooled down to room temperature. Butyl acetate and the product were separated from the ionic liquid phase to reuse the ionic liquid, and then, butyl acetate was distilled for reuse; subsequently, the product remained in the ionic liquid phase, which was dissolved in EtOAc (1 ml). The product was obtained by isolation with preparative TLC (eluting solution: petroleum ether/ethyl acetate = 10/1 v/v). The desired product 5a was obtained in an 89–91% yield. At the end, the Brønsted acid ionic liquid catalyst was recycled several times; after this, a sodium chloride (3 × 0.5 ml) solution was added to dissolve the ionic liquid; then, EtOAc (3 × 0.5 ml) was added to extract the remaining desired product.

### Cell lines

The HeLa cells and A549 cells were purchased from Shanghai Zhong Qiao Xin Zhou Biotechnology Co., Ltd. The cells were cultured in the Dulbecco's modified Eagle medium (Hyclone, Thermo Scientific) and supplemented with 10% fetal bovine serum (Zhejiang Tianhang Biotechnology Co., Ltd). The cells were cultured under a 5% CO_2_ atmosphere at 37 °C.

### *In vitro* cytotoxicity study (MTT assay)

Cytotoxicity was estimated using an MTT assay. The cells were seeded in 96-well plates and cultured overnight to reach a ∼80% confluency. Fresh media containing the compounds were incubated with cells for 48 h. Then, 100 μl of 0.5 mg ml^−1^ thiazolyl blue tetrazolium bromide (MTT, in the DMEM medium) solution was added to each well, followed by a 4 h incubation at 37 °C. Then, the medium was removed, and 150 μl dimethyl sulfoxide (DMSO) was added. The optical density of the final solution was measured by a micro plate reader at the wavelength of 490 nm.

### Spectroscopic data of the obtained products

#### Ethyl 2-(2-methyl-1*H*-indol-3-yl)acrylate (4a)

1a (19.11 mg, 0.03 mmol) was mixed with 2-methyl indole (39.35 mg, 0.3 mmol) and ethyl pyruvate (34.58 mg, 0.3 mmol) to obtain 4a in a 91% yield (62.58 mg); a red oil: ^1^H NMR (400 MHz, CDCl_3_, 25 °C, TMS): *δ* = 7.95 (s, 1H), 7.33 (s, 1H), 6.99–6.98 (m, 3H), 6.44 (s, 1H), 5.69 (s, 1H), 4.19 (q, *J* = 7.1 Hz, 2H), 2.09 (s, 3H), 1.20 ppm (t, *J* = 7.1 Hz, 3H); ^13^C NMR (100 MHz, CDCl_3_) *δ* = 167.8, 135.1, 134.7, 133.6, 127.9, 127.2, 121.3, 119.8, 118.8, 110.6, 109.6, 77.1, 61.2, 14.3, 12.5 ppm. IR (cm^−1^): 3394, 3055, 2981, 293 351, 1708, 1614, 1550, 1459, 1370, 1281, 1177. HRMS-ESI (*m*/*z*) calcd for C_14_H_15_NO_2_, [M + Na]^+^ 229.1103, found 252.0997.

#### Ethyl 2-(4-bromo-1-methyl-1*H*-indol-3-yl)acrylate (4b)

1a (19.11 mg, 0.03 mmol) was mixed with 4-bromo-1-methyl-1*H*-indole (63.02 mg, 0.3 mmol) and ethyl pyruvate (34.58 mg, 0.3 mmol) to obtain 4b in a 90% yield (82.9 mg); a gum-like white oil: ^1^H NMR (400 MHz, DMSO, 25 °C, TMS): *δ* = 7.48 (d, *J* = 8.2 Hz, 1H), 7.43 (s, 1H), 7.24 (d, *J* = 7.5 Hz, 1H), 7.08 (t, *J* = 7.9 Hz, 1H), 6.28 (s, 1H), 5.78 (s, 1H), 4.15 (q, *J* = 7.1 Hz, 2H), 3.79 (s, 3H), 1.15 ppm (t, *J* = 7.1 Hz, 3H); ^13^C NMR (100 MHz, DMSO, 25 °C) *δ* = 167.4, 138.1, 135.9, 131.4, 127.1, 125.6, 123.7, 123.0, 113.8, 112.6, 110.2, 61.0, 40.0, 33.1, 14.5 ppm. IR (cm^−1^): 2929, 1721, 1606, 1456, 1347, 1165. HRMS-ESI (*m*/*z*) calcd for C_14_H_14_BrNO_2_, [M + Na]^+^ 330.0106, found 330.0101.

#### Ethyl 2-(1-ethyl-2-methyl-1*H*-indol-3-yl)acrylate (4c)

1a (19.11 mg, 0.03 mmol) was mixed with 1-ethyl-2-methyl-1*H*-indole (47.76 mg, 03 mmol) and ethyl pyruvate (34.58 mg, 0.3 mmol) to obtain 4c in a 96% yield (74.11 mg); a yellow oil: ^1^H NMR (400 MHz, CDCl_3,_ 25 °C, TMS): *δ* = 7.36 (d, *J* = 7.7 Hz, 1H), 7.18 (d, *J* = 8.1 Hz, 1H), 7.06 (t, *J* = 7.4 Hz, 1H), 6.99 (t, *J* = 7.4 Hz, 1H), 6.46 (s, 1H), 5.69 (s, 1H), 4.18 (q, *J* = 7.1 Hz, 2H), 4.03 (q, *J* = 7.1 Hz, 2H), 2.25 (s, 3H), 1.25 (t, *J* = 7.2 Hz, 3H), 1.20 ppm (t, *J* = 7.1 Hz, 3H); ^13^C NMR (100 MHz, CDCl_3,_ 25 °C) *δ* = 167.8, 135.5, 135.1, 134.2, 127.5, 127.3, 121.0, 119.6, 119.0, 109.4, 108.9, 61.1, 38.0, 15.3, 14.3, 11.3 ppm. IR (cm^−1^): 3050, 2979, 2934, 1717, 1616, 1466, 1371, 1349, 1194, 1142. HRMS-ESI (*m*/*z*) calcd for C_16_H_19_NO_2_, [M + Na]^+^ 280.1314, found 280.1309.

#### Ethyl 2-(6-fluoro-2-methyl-1*H*-indol-3-yl)acrylate (4d)

1a (19.11 mg, 0.03 mmol) was mixed with 6-fluoro-2-methyl-1*H*-indole (44.75 mg, 0.3 mmol) and ethyl pyruvate (34.58 mg, 0.3 mmol) to obtain 4d in a 93% yield (68.98 mg); a yellow oil: ^1^H NMR (400 MHz, CDCl_3_, 25 °C, TMS): *δ* = 8.20 (s, 1H), 7.06 (dd, *J* = 9.7, 1.5 Hz, 1H), 6.96 (dd, *J* = 8.7, 4.4 Hz, 1H), 6.80 (td, *J* = 9.1, 2.1 Hz, 1H), 6.53 (s, 1H), 5.77 (s, 1H), 4.29 (q, *J* = 7.1 Hz, 2H), 2.19 (s, 3H), 1.31 ppm (t, *J* = 7.1 Hz, 3H); ^13^C NMR (100 MHz, CDCl_3,_ 25 °C) *δ* = 167.6 (s), 159.3 (s), 157.0 (s), 135.5 (s), 134.3 (s), 131.5 (s), 128.4 (d, *J* = 9.9 Hz), 127.50 (s), 111.0 (d, *J* = 9.6 Hz), 109.8 (d, *J* = 4.1 Hz), 109.5 (s), 109.2 (s), 104.0 (s), 103.8 (s), 61.3 (s), 14.2 (s), 12.6 ppm (s). ^19^F NMR (375 MHz, CDCl_3_) *δ* = −124.39 to −124.58 (m) ppm. IR (cm^−1^): 3359, 2983, 2933, 1703, 1613, 1486, 1303, 1188, 1146. HRMS-ESI (*m*/*z*) calcd for C_14_H_14_FNO_2_, [M + Na]^+^ 270.0907, found 270.0902.

#### Ethyl 2-(2-phenyl-1*H*-indol-3-yl)acrylate (4e)

1a (19.11 mg, 0.03 mmol) was mixed with 2-phenyl-1*H*-indole (57.97 mg, 0.3 mmol) and ethyl pyruvate (34.58 mg, 0.3 mmol) to obtain 4e in an 88% yield (76.91 mg); a yellow pale oil: ^1^H NMR (400 MHz, CDCl_3_, 25 °C, TMS): *δ* = 8.78 (s, 1H), 7.60 (d, *J* = 7.6 Hz, 1H), 7.49 (d, *J* = 7.4 Hz, 2H), 7.36 (t, *J* = 7.7 Hz, 3H), 7.29 (t, *J* = 7.1 Hz, 1H), 7.25–7.13 (m, 2H), 6.59 (s, 1H), 6.00 (s, 1H), 3.86 (q, *J* = 7.1 Hz, 2H), 0.86 ppm (t, *J* = 7.1 Hz, 3H); ^13^C NMR (100 MHz, CDCl_3,_ 25 °C) *δ* = 167.6, 135.9, 135.9, 135.1, 133.2, 128.8, 128.6, 128.1, 127.8, 127.5, 122.5, 120.4, 119.3, 111.2, 109.8, 60.9, 13.7 ppm. IR (cm^−1^): 3330, 3056, 2980, 2802, 1701, 1618, 1455, 1276, 1184. HRMS-ESI (*m*/*z*) calcd for C_19_H_17_NO_2_, [M + Na]^+^ 314.1157, found 314.1151.

#### Ethyl 2-(1-methyl-2-phenyl-1*H*-indol-3-yl)acrylate (4f)

1a (19.11 mg, 0.03 mmol) was mixed with 1-methyl-2-phenyl-1*H*-indole (62.18 mg, 0.3 mmol) and ethyl pyruvate (34.58 mg, 0.3 mmol) to obtain 4f in a 91% yield (83.36 mg); a yellow pale oil: ^1^H NMR (400 MHz, CDCl_3_, 25 °C, TMS): *δ* = 7.52 (d, *J* = 7.9 Hz, 1H), 7.29 (dt, *J* = 25.5, 9.5 Hz, 6H), 7.16 (t, *J* = 7.3 Hz, 1H), 7.07 (t, *J* = 7.3 Hz, 1H), 6.29 (s, 1H), 5.73 (s, 1H), 3.72 (q, *J* = 7.1 Hz, 2H), 3.55 (s, 3H), 0.88 ppm (t, *J* = 7.1 Hz, 3H); ^13^C NMR (100 MHz, CDCl_3,_ 25 °C) *δ* = 167.8, 139.1, 137.3, 135.3, 132.00, 130.5, 128.5, 128.2, 127.2, 127.0, 122.2, 120.4, 119.5, 110.7, 109.8, 60.8, 31.1, 14.0 ppm. IR (cm^−1^): 3054, 2980, 2937, 1889, 1717, 1613, 1467, 1371, 1226, 1099. IR (cm^−1^): 3054, 2979, 2901, 1718, 1614, 1462, 1345, 1264, 1213, 1104. HRMS-ESI (*m*/*z*) calcd for C_20_H_19_NO_2_, [M + Na]^+^ 328.1314, found 328.1308.

#### Ethyl 2-(1-ethyl-2-phenyl-1*H*-indol-3-yl)acrylate (4g)

1a (19.11 mg, 0.03 mmol) was mixed with 1-ethyl-2-phenyl-1*H*-indole (66.39 mg, 0.3 mmol) and ethyl pyruvate (34.58 mg, 0.3 mmol) to obtain 4g in a 91% yield (86.23 mg); a yellow pale oil: ^1^H NMR (400 MHz, CDCl_3_, 25 °C, TMS): *δ* = 7.53 (d, *J* = 7.8 Hz, 1H), 7.35–7.25 (m, 6H), 7.15 (t, *J* = 7.3 Hz, 1H), 7.07 (t, *J* = 7.3 Hz, 1H), 6.25 (s, 1H), 5.71 (s, 1H), 4.02 (q, *J* = 7.1 Hz, 2H), 3.73 (q, *J* = 7.1 Hz, 2H), 1.17 (t, *J* = 7.1 Hz, 3H), 0.91 ppm (t, *J* = 7.1 Hz, 3H); ^13^C NMR (100 MHz, CDCl_3,_ 25 °C) *δ* = 167.8, 138.7, 136.1, 135.3, 132.3, 130.5, 128.5, 128.3, 127.5, 126.8, 122.1, 120.3, 119.7, 111.0, 110.0, 60.7, 38.9, 15.4, 14.0 ppm. IR (cm^−1^): 3054, 2979, 2935, 1718, 1614, 1462, 1345, 1213, 1142, 1104, 1026. HRMS-ESI (*m*/*z*) calcd for C_21_H_21_NO_2_, [M + Na]^+^ 342.1470, found 342.1464.

#### Ethyl 2-(5-chloro-2-methyl-1*H*-indol-3-yl)acrylate (4h)

1a (19.11 mg, 0.03 mmol) was mixed with 5-chloro-2-methyl-1*H*-indole (49.68 mg, 0.3 mmol) and ethyl pyruvate (34.58 mg, 0.3 mmol) to obtain 4h in a 92% yield (72.78 mg); a yellow oil: ^1^H NMR (400 MHz, CDCl_3_, 25 °C, TMS): *δ* = 8.12 (s, 1H), 7.38 (s, 1H), 7.06 (q, *J* = 8.5 Hz, 2H), 6.57 (s, 1H), 5.79 (s, 1H), 4.29 (q, *J* = 7.0 Hz, 2H), 2.27 (s, 3H), 1.32 ppm (t, *J* = 7.1 Hz, 3H); ^13^C NMR (100 MHz, CDCl_3,_ 25 °C) *δ* = 167.3, 134.9, 134.1, 133.4, 129.1, 128.0, 125.6, 121.5, 118.4, 111.3, 109.6, 61.2, 14.2, 12.6 ppm. IR (cm^−1^): 3349, 2982, 2928, 1701, 1617, 1472, 1302, 1175, 1083. HRMS-ESI (*m*/*z*) calcd for C_14_H_14_ClNO_2_, [M + Na]^+^ 286.06053, found 286.06084.

#### Ethyl 3-(3-ethoxy-3-oxoprop-1-en-2-yl)-1*H*-indole-2-carboxylate (4i)

1a (19.11 mg, 0.03 mmol) was mixed with ethyl 1*H*-indole-2-carboxylate (56.76 mg, 0.3 mmol) and ethyl pyruvate (34.58 mg, 0.3 mmol) to obtain 4i in a 93% yield (80.15 mg); a yellow pale oil: ^1^H NMR (400 MHz, CDCl_3_, 25 °C, TMS): *δ* = 9.47 (s, 1H), 7.63 (d, *J* = 8.1 Hz, 1H), 7.40 (d, *J* = 8.3 Hz, 1H), 7.31 (t, *J* = 7.6 Hz, 1H), 7.16 (t, *J* = 7.5 Hz, 1H), 6.66 (s, 1H), 5.92 (s, 1H), 4.32 (q, *J* = 7.1 Hz, 2H), 4.24 (q, *J* = 7.1 Hz, 2H), 1.34 (t, *J* = 7.1 Hz, 3H), 1.25 ppm (t, *J* = 7.1 Hz, 3H); ^13^C NMR (100 MHz, CDCl_3,_ 25 °C) *δ* = 167.1, 161.8, 135.9, 134.5, 128.1, 127.4, 125.6, 124.3, 121.0, 120.8, 118.4, 112.1, 61.2, 61.0, 14.2 ppm. IR (cm^−1^): 3332, 3062, 2983, 2904, 1707, 1538, 1461, 1246, 1023. HRMS-ESI (*m*/*z*) calcd for C_21_H_21_NO_2_, [M + Na]^+^ 319.1572, found 342.1464.

#### Ethyl 3-(3-ethoxy-3-oxoprop-1-en-2-yl)-5-methoxy-1*H*-indole-2-carboxylate (4j)

1a (19.11 mg, 0.03 mmol) was mixed with ethyl 5-methoxy-1*H*-indole-2-carboxylate (65.77 mg, 0.3 mmol) and ethyl pyruvate (34.58 mg, 0.3 mmol) to obtain 4j in an 80% yield (76.16 mg); a yellow pale oil: ^1^H NMR (400 MHz, CDCl_3_, 25 °C, TMS): *δ* = 9.03 (s, 4H), 7.32 (d, *J* = 8.6 Hz, 5H), 7.02 (s, 9H), 6.66 (s, 5H), 5.92 (s, 5H), 4.33 (q, *J* = 7.1 Hz, 10H), 4.23 (q, *J* = 7.1 Hz, 10H), 3.84 (s, 15H), 1.35 (t, *J* = 7.1 Hz, 16H), 1.26 ppm (t, *J* = 7.0 Hz, 18H); ^13^C NMR (100 MHz, CDCl_3,_ 25 °C) *δ* = 167.1, 161.2, 155.1, 134.6, 131.1, 127.9, 127.8, 124.8, 118.1, 117.4, 112.9, 100.8, 61.1, 61.0, 55.7, 14.2 ppm. IR (cm^−1^): 3330, 2983, 2935, 1707, 1625, 1468, 1215, 1028. HRMS-ESI (*m*/*z*) calcd for C_16_H_17_NO_4_, [M + Na]^+^ 310.1056, found 310.1050.

#### Ethyl 3-(3-ethoxy-3-oxoprop-1-en-2-yl)-5-fluoro-1*H*-indole-2-carboxylate (4k)

1a (19.11 mg, 0.03 mmol) was mixed with ethyl 5-fluoro-1*H*-indole-2-carboxylate (62.16 mg, 0.3 mmol) and ethyl pyruvate (34.58 mg, 0.3 mmol) to obtain 4k in a 70% yield (64.11 mg); a white solid: mp = 132–133 °C; ^1^H NMR (400 MHz, CDCl_3_, 25 °C, TMS): *δ* = 9.10 (s, 2H), 7.36 (dd, *J* = 8.9, 4.2 Hz, 2H), 7.25 (s, 2H), 7.11 (td, *J* = 8.9, 2.0 Hz, 2H), 6.65 (s, 2H), 5.90 (s, 2H), 4.34 (q, *J* = 7.1 Hz, 4H), 4.26 (s, 4H), 1.35 (t, *J* = 7.1 Hz, 7H), 1.27 ppm (d, *J* = 7.1 Hz, 7H); ^13^C NMR (100 MHz, CDCl_3,_ 25 °C) *δ* = 166.8 (s), 161.3 (s), 159.7 (s), 134.1 (s), 132.3 (s), 128.5–128.3 (m), 128.0 (d, *J* = 39.1 Hz), 125.9 (s), 118.4 (s), 115.0 (s), 114.8 (s), 113.0 (d, *J* = 9.4 Hz), 105.4 (s), 105.2 (s), 61.4 (s), 61.0 (s), 29.3 (s), 14.2 ppm (s). ^19^F NMR (375 MHz, CDCl_3_) *δ* = −121.96 to −122.02 (m) ppm. IR (cm^−1^): 3392, 3312, 2956, 2922, 2851, 1712, 1470, 1248, 1186. HRMS-ESI (*m*/*z*) calcd for C_16_H_16_FNO_4_, [M + Na]^+^ 328.0961, found 328.0955.

#### 3-(2-Methyl-1*H*-indol-3-yl)but-3-en-2-one (4l)

1a (19.11 mg, 0.03 mmol) was mixed with 2-methyl-1*H*-indole (39.35 mg, 0.3 mmol) and methyl pyruvate (25.82 mg, 0.3 mmol) to obtain 4l in an 87% yield (52.01 mg); a yellow oil: ^1^H NMR (400 MHz, CDCl_3_, 25 °C, TMS): *δ* = 8.08 (s, 3H), 7.35 (d, *J* = 7.6 Hz, 3H), 7.27–7.23 (m, 4H), 7.15–7.06 (m, 7H), 6.36 (s, 3H), 5.83 (s, 3H), 2.38 (s, 9H), 2.30 ppm (s, 9H); ^13^C NMR (100 MHz, CDCl_3,_ 25 °C) *δ* = 200.8, 142.9, 135.2, 133.1, 128.1, 126.2, 121.5, 120.0, 118.6, 110.4, 110.2, 27.4, 12.6 ppm. IR (cm^−1^): 3393, 3056, 2923, 2853, 1678, 1604, 1460, 1359. HRMS-ESI (*m*/*z*) calcd for C_13_H_13_NO, [M + Na]^+^ 222.08894, found 222.08925.

#### Methyl 2-(2-methyl-1*H*-indol-3-yl)acrylate (4m)

1a (19.11 mg, 0.03 mmol) was mixed with 2-methyl-1*H*-indole (39.35 mg, 0.3 mmol) and methyl 2-oxopropanoate (30.78 mg, 0.3 mmol) to obtain 4m in a 95% yield (61.34 mg); a yellow oil: ^1^H NMR (400 MHz, CDCl_3_, 25 °C, TMS): *δ* = 8.03 (s, 1H), 7.41 (d, *J* = 6.9 Hz, 1H), 7.17 (d, *J* = 7.6 Hz, 1H), 7.12–7.06 (m, 2H), 6.55 (s, 1H), 5.80 (s, 1H), 3.79 (s, 3H), 2.26 ppm (s, 3H); ^13^C NMR (100 MHz, CDCl_3_, 25 °C) *δ* = 168.2, 168.2, 135.0, 134.2, 133.4, 133.4, 127.9, 127.5, 121.3, 119.9, 118.6, 110.4, 109.5, 109.5, 77.3, 77.0, 76.7, 52.1, 12.4 ppm. IR (cm^−1^): 3393, 3056, 2950, 2922, 1707, 1621, 1460, 1437, 1285, 1168. HRMS-ESI (*m*/*z*) calcd for C_13_H_13_NO_2_, [M + Na]^+^ 238.08385, found 238.08415.

#### 2-(2-Methyl-1*H*-indol-3-yl)-1-phenylprop-2-en-1-one (4n)

1a (19.11 mg, 0.03 mmol) was mixed with 2-methyl-1*H*-indole (39.35 mg, 0.3 mmol) and 1-phenylpropane-1,2-dione (44.44 mg, 0.3 mmol) to obtain 4n in a 92% yield (72.12 mg); a yellow oil: ^1^H NMR (400 MHz, CDCl_3_, 25 °C, TMS) *δ* = 8.02 (brs, 1H), 7.90 (d, *J* = 7.6 Hz, 2H), 7.60–7.47 (m, 2H), 7.38 (t, *J* = 7.5 Hz, 2H), 7.24 (s, 1H), 7.11 (dd, *J* = 13.6, 6.9 Hz, 2H), 6.01 (s, 1H), 5.92 (s, 1H), 2.22 ppm (s, 3H); ^13^C NMR (100 MHz, DMSO, 25 °C) *δ* = 197.9, 143.0, 137.4, 135.7, 134.5, 133.5, 129.7, 129.3, 129.0, 128.3, 127.1, 121.4, 121.2, 119.9, 118.4, 111.3, 109.8, 13.0 ppm. IR (cm^−1^): 2960, 2930, 2871, 1720, 1459, 1281, 1062. HRMS-ESI (*m*/*z*) calcd for C_18_H_15_NO, [M + Na]^+^ 284.10459, found 248.10505.

#### (*E*)-Hex-3-en-1-yl 2-(2-methyl-1*H*-indol-3-yl)acrylate (4o)

1a (19.11 mg, 0.03 mmol) was mixed with 2-methyl-1*H*-indole (39.35 mg, 0.3 mmol) and (*E*)-hex-3-en-1-yl 2-oxopropanoate (51.06 mg, 0.3 mmol) to obtain 4o in a 94% yield (79.90 mg); a yellow oil: ^1^H NMR (400 MHz, CDCl_3_, 25 °C, TMS): *δ* = 8.01 (s, 1H), 7.42 (d, *J* = 6.9 Hz, 1H), 7.17 (d, *J* = 7.0 Hz, 1H), 7.12–7.04 (m, 2H), 6.54 (s, 1H), 5.80 (s, 1H), 5.48 (dd, *J* = 17.3, 7.3 Hz, 1H), 5.31 (dd, *J* = 16.9, 7.5 Hz, 1H), 4.22 (d, *J* = 7.0 Hz, 2H), 2.42 (dd, *J* = 13.6, 6.7 Hz, 2H), 2.27 (s, 3H), 2.06–1.97 (m, 2H), 0.93 ppm (t, *J* = 7.5 Hz, 3H); ^13^C NMR (100 MHz, CDCl_3,_ 25 °C) *δ* = 167.67, 135.06, 134.7, 134.6, 133.4, 133.4, 128.0, 127.4, 123.7, 121.4, 119.9, 118.9, 110.4, 109.7, 64.7, 26.8, 20.6, 14.2, 12.6 ppm. IR (cm^−1^): 3393, 3057, 2962, 2929, 1704, 1612, 1460, 1388, 1284, 1172, 1079. HRMS-ESI (*m*/*z*) calcd for C_18_H_21_NO_2_, [M + Na]^+^ 306.14645, found 306.14672.

#### Ethyl (*E*)-2-(2-methyl-1*H*-indol-3-yl)hex-2-enoate (4p)

1a (19.11 mg, 0.03 mmol) was mixed with 2-methyl-1*H*-indole (39.35 mg, 0.3 mmol) and ethyl 2-oxohexanoate (47.45 mg, 0.3 mmol) to obtain 4p in a 95% yield (81.40 mg); a red solid: mp = 85–86 °C; ^1^H NMR (400 MHz, CDCl_3_, 25 °C, TMS): *δ* = 7.90 (s, 1H), 7.44 (d, *J* = 7.2 Hz, 1H), 7.18 (d, *J* = 7.7 Hz, 1H), 7.09 (dd, *J* = 13.1, 6.6 Hz, 2H), 6.13 (t, *J* = 7.5 Hz, 1H), 4.24 (q, *J* = 6.9 Hz, 2H), 2.57 (q, *J* = 7.3 Hz, 2H), 2.28 (s, 3H), 1.63–1.50 (m, 2H), 1.25 (t, *J* = 7.0 Hz, 3H), 1.01 ppm (t, *J* = 7.2 Hz, 3H); ^13^C NMR (100 MHz, CDCl_3,_ 25 °C) *δ* = 168.7, 143.5, 135.0, 132.5, 128.2, 126.9, 121.2, 119.7, 118.7, 111.5, 110.4, 60.5, 32.0, 22.9, 14.3, 13.9, 12.4 ppm. IR (cm^−1^): 3396, 3057, 2960, 2930, 2870, 1699, 1623, 1556, 1460, 1212, 1172. HRMS-ESI (*m*/*z*) calcd for C_17_H_21_NO_2_, [M + Na]^+^ 294.14645, found 294.14674.

#### 2-Methyl-3-(3,3,3-trifluoroprop-1-en-2-yl)-1*H*-indole (4q)

1a (19.11 mg, 0.03 mmol) was mixed with 2-methyl-1*H*-indole (39.35 mg, 0.3 mmol) and 1,1,1-trifluoropropan-2-one (33.61 mg, 0.3 mmol) to obtain 4q in an 88% yield (59.45 mg); a reddish brown oil: ^1^H NMR (400 MHz, DMSO, 25 °C, TMS): *δ* = 11.34 (s, 1H), 7.33 (dd, *J* = 7.3, 4.2 Hz, 2H), 7.07 (t, *J* = 7.4 Hz, 1H), 7.00 (t, *J* = 7.4 Hz, 1H), 6.22 (s, 1H), 5.71 (s, 1H), 2.35 ppm (s, 3H); ^13^C NMR (100 MHz, DMSO, 25 °C) *δ* = 136.0–135.8 (m), 135.5 (d, *J* = 17.5 Hz), 132.9 (s), 132.6 (s), 132.3 (s), 132.0 (s), 127.9 (s), 125.5 (s), 124.6 (dd, *J* = 10.1, 5.0 Hz), 122.8 (s), 121.3 (s), 119.9 (s), 118.4 (s), 111.3 (s), 105.3 (s), 12.2 ppm (s). ^19^F NMR (375 MHz, DMSO) *δ* = −64.49 (s). IR (cm^−1^): 3478, 3403, 2927, 1663, 1460, 1289, 1168, 1125. HRMS-ESI (*m*/*z*) calcd for C_12_H_10_F_3_N, [M + H]^+^ 226.08381, found 226.08416.

#### Diethyl (*E*)-4-((*E*)-2,5-dimethyl-3*H*-indol-3-ylidene)-2-methylpent-2 enedioate (4r)

1a (19.11 mg, 0.03 mmol) was mixed with 2,5-dimethyl-1*H*-indole (43.56 mg, 0.3 mmol) and ethyl pyruvate (34.58 mg, 0.3 mmol) to obtain 4r in a 91% yield (84.46 mg); a gum-like brown oil: ^1^H NMR (400 MHz, CDCl_3_, 25 °C, TMS): *δ* = 7.81 (s, 1H), 6.90 (d, *J* = 8.2 Hz, 1H), 6.72 (d, *J* = 8.2 Hz, 1H), 6.00 (s, 1H), 4.27 (q, *J* = 7.1 Hz, 2H), 4.08 (dd, *J* = 13.3, 6.3 Hz, 2H), 2.32 (s, 3H), 2.22 (s, 3H), 1.57 (s, 3H), 1.30 (t, *J* = 7.1 Hz, 3H), 1.13 ppm (t, *J* = 7.1 Hz, 3H); ^13^C NMR (100 MHz, CDCl_3,_ 25 °C) *δ* = 173.5, 170.7, 134.7, 131.5, 131.4, 130.9, 126.0, 125.8, 124.0, 119.1, 110.8, 109.4, 61.4, 45.9, 27.2, 19.7, 14.1, 13.0 ppm. IR (cm^−1^): 3392, 2980, 2931, 1725, 1453, 1369, 1232, 1100. HRMS-ESI (*m*/*z*) calcd for C_20_H_23_NO_4_, [M + Na]^+^ 364.1525, found 364.1517.

#### Diethyl (*E*)-4-((*E*)-5-methoxy-2-methyl-3*H*-indol-3-ylidene)-2-methylpent-2-enedioate (4s)

1a (19.11 mg, 0.03 mmol) was mixed with 5-methoxy-2-methyl-1*H*-indole (48.36 mg, 0.3 mmol) and ethyl pyruvate (34.58 mg, 0.3 mmol) to obtain 4s in an 87% yield (94.62 mg); a gum-like reddish brown oil: ^1^H NMR (400 MHz, CDCl_3_, 25 °C, TMS): *δ* = 7.81 (s, 1H), 7.03 (d, *J* = 8.7 Hz, 1H), 6.68 (d, *J* = 8.7 Hz, 1H), 5.89 (s, 1H), 4.34 (q, *J* = 7.1 Hz, 2H), 4.15 (dd, *J* = 13.9, 6.9 Hz, 2H), 3.78 (s, 3H), 2.42 (s, 3H), 1.64 (s, 3H), 1.37 (t, *J* = 7.1 Hz, 3H), 1.22 ppm (t, *J* = 7.1 Hz, 3H); ^13^C NMR (100 MHz, CDCl_3,_ 25 °C) *δ* = 173.6, 170.3, 148.4, 133.0, 131.2, 129.1, 127.8, 126.3, 111.4, 109.4, 109.3, 109.2, 61.4, 61.1, 57.6, 46.1, 27.4, 14.3, 13.2 ppm. IR (cm^−1^): 3366, 2980, 2932, 2836, 1725, 1503, 1452, 1276, 1233. IR (cm^−1^): 3392, 2980, 2931, 2870, 1725, 1453, 1232, 1100. HRMS-ESI (*m*/*z*) calcd for C_20_H_23_NO_5_, [M + Na]^+^ 380.1474, found 380.1466.

#### Diethyl 1,4-dimethyl-3-(1-methyl-1*H*-indol-3-yl)-1,2,3,4-tetrahydrocyclopenta[*b*]indole-1,3-dicarboxylate (5a)

1a (19.11 mg, 0.03 mmol) was mixed with 1-methyl-1*H*-indole (39.35 mg, 0.3 mmol) and ethyl pyruvate (34.58 mg, 0.3 mmol) to obtain 5a in a 92% yield (126.55 mg); a yellow solid: mp = 130–131 °C; ^1^H NMR (400 MHz, DMSO, 25 °C, TMS): *δ* = 7.59 (d, *J* = 7.6 Hz, 1H), 7.42 (d, *J* = 8.2 Hz, 1H), 7.36 (d, *J* = 8.1 Hz, 1H), 7.20 (s, 1H), 7.12 (dt, *J* = 15.1, 7.3 Hz, 3H), 6.82 (t, *J* = 7.4 Hz, 1H), 6.65 (d, *J* = 7.9 Hz, 1H), 4.36–4.14 (m, 2H), 4.02 (dd, *J* = 18.8, 11.6 Hz, 3H), 3.41 (s, 3H), 3.30 (s, 3H), 2.67 (d, *J* = 13.6 Hz, 1H), 1.67 (s, 3H), 1.24 (t, *J* = 7.0 Hz, 3H), 1.13 ppm (t, *J* = 7.0 Hz, 3H); ^13^C NMR (100 MHz, DMSO, 25 °C) *δ* = 175.5, 172.5, 143.6, 142.0, 137.8, 127.4, 125.5, 122.5, 121.9, 121.4, 121.0, 119.8, 119.6, 119.1, 118.8, 114.3, 110.8, 110.6, 61.7, 60.8, 56.8, 53.9, 48.1, 32.8, 30.7, 25.3, 14.5, 14.4 ppm. IR (cm^−1^): 3053, 2978, 2931, 1728, 1469, 1246, 1107. HRMS-ESI (*m*/*z*) calcd for C_28_H_30_N_2_O_4_, [M + Na]^+^ 481.2104, found 481.2095.

## Conflicts of interest

The authors declare that they have no competing financial interest.

## Supplementary Material

RA-010-D0RA00990C-s001

RA-010-D0RA00990C-s002
