# Supplementary material for: Synthesis of α-indolylacrylates as potential anticancer agents using a Brønsted acid ionic liquid catalyst and the butyl acetate solvent
Source: RSC Adv. 2020 Apr 2;10(23):13507–16. doi: 10.1039/d0ra00990c (PMC9051412; doi:10.1039/d0ra00990c)
Supplement: RA-010-D0RA00990C-s001 [file RA-010-D0RA00990C-s001.pdf]

Electronic Supplementary Material (ESI) for RSC Advances.
This journal is © The Royal Society of Chemistry 2020

Synthesis of α-Indolylacrylates as potential anti-cancer agents by Brønsted acid ionic liquid catalyst and butyl acetate solvent†

Ahmed El-Harairy, *[a,b] Mennatallah Shaheen,[c] Jun Li,[b] Yuzhou Wu,[b] Minghao Li,[b] and Yanlong Gu [a, d]

[a] Environmental, Energy and Green Chemistry Laboratory, Faculty of Agriculture, Damietta University, 34511, Damietta, Egypt
[b] School of Chemistry and Chemical Engineering, Huazhong University of Science and Technology, 430074, Wuhan, China.
[c] Department of Pharmaceutical Chemistry, Faculty of Pharmacy, Horus University-Egypt, 34511, New Damietta, Damietta, Egypt.
[d] State Key Laboratory for Oxo Synthesis and Selective Oxidation, Lanzhou Institute of Chemical Physics, Lanzhou, 730000, China.

1. Synthesis of ionic liquid 1a[1]

Brønsted acid IL **1a** was synthesized through the following three steps: (**i**) synthesis of **2a**: in 100 ml of around bottomed flask equipped with mechanical stirring, equal amount of divinyl sulfone **2a** (5.0 g, 42.3 mmol) was mixed with 1-(3-aminopropanyl)imidazole (5.2 g, 42.3 mmol) in 75 ml of methanol; the mixture was stirred at 60 °C for 24 h; then, volatile methanol was removed by a rotary evaporator; a yellow-pale oil was obtained, which is **2a**, in nearly quantitative yield. (**ii**) synthesis of **3a** via quaternization: **2a** (10.0 g, 41.1 mmol), 1,3-propanesulfonate (10.0 g, 82.3 mmol), and acetonitrile (75 ml) were mixed in a 250 ml of around bottomed flask equipped with mechanical stirring; the mixture was stirred at 80 °C for 24 h; a yellow solid was generated; then the solvent was decanted out; the yellow solid was filtrated, and washed with acetone (5.0 ml × 3); **3a** can be obtained as white solid; then, the white solid was dried at 60 °C under vacuum (20 mmHg) for 4 h; and (**iii**) acidification: **3a** (10.0 g, 20.5 mmol) was mixed with triflic acid (3.1 g, 20.5 mmol) in 25 mL of around bottomed flask; to facilitate the reaction, a small amount of water (0.25 ml) was also added in this step. Then, the mixture was stirred at 100 °C for 24 h. The generated ionic liquid was washed with ethyl acetate (5.0 ml × 3) and diethyl ether (5.0 ml × 3); then, water and volatile solvents were removed under reduced pressure; finally, **1a** was obtained as a yellow-pale viscous liquid. Through all these three-step reactions, the ionic liquid **1a** can be synthesized in 88% yield.

2. Synthesis of Forbes's ionic liquid 1b[2]

1-Methylimidazole (8.2 g, 0.1 mol) is stirred under solvent-free conditions with 1,3-propanesultone (12.2 g, 0.1 mol) at 50 oC for 6 hours. After reaction, the obtained viscous liquid was washed with ethyl acetate (10 ml × 3) and then dried under vacuum (10 mmHg) for 2 hours. With this procedure, 3-(1-methyl-3-imidazolio)propanesulfonate was obtained in 97 % of yield (mp. = 215-216 oC). In a 100 ml of round bottomed flask equipped with mechanical stirring, 3-(1-methyl-3-imidazolio)propanesulfonate (10.2 g, 50 mmol) was mixed with trifloromethanesulfonic acid (7.5 g, 50 mmol). The mixture was then heated at 80 ˚C for 6 hours under stirring. Then, the generated viscous liquid washed with ethyl acetate (10 ml × 3). After 6 hours of drying at 60 °C under vacuum (20 mmHg), Forbes's IL **1b** was obtained as a brown viscous liquid (16.8 g, 95 %).

3. Synthesis of ionic liquid 1c[3]

In 250 ml of round bottomed flask equipped with mechanical stirring, divinyl sulfone **2c** (11.8 g, 0.10 mol) was mixed with n-butylamine (7.3 g, 0.10 mol) in methanol (150 ml). The mixture was then stirred at 60 °C for 5 hours. Methanol was removed by a rotary evaporator under vacuum, and **3c** was obtained as a color-less oil quantitatively. The obtained **3c** was then mixed with 1,3-propane sultone (13.4 g, 0.11 mol) in acetonitrile (150 ml). The solution was refluxed for 5 hours. Then, the generated white solid was filtrated, and washed with acetone (10 ml × 3). After 6 hours of drying at 60 °C under vacuum (20 mmHg), **4c** was obtained as a white powder (23.1 g, 74 %). In a 100 ml of round bottomed flask, **4c** (15.6 g, 50 mmol) was mixed with trifluoromethanesulfonic acid (7.5 g, 50 mmol). Then, the mixture was stirred at 80 °C for 8 hours. In order to facilitate the stirring, water (1.0 ml) was added into the system. The formed liquid was washed with ethyl acetate (15 ml × 3) and diethyl ether (15 ml × 3). After that, it was dried at 80 °C under vacuum (10 mmHg) for 4 hours. Finally, IL **1c** was obtained as a viscous brown oil (22.2g, 96 %).

4. Figure S1: X-ray Single Crystal Data for 5a

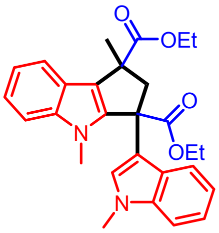

5a

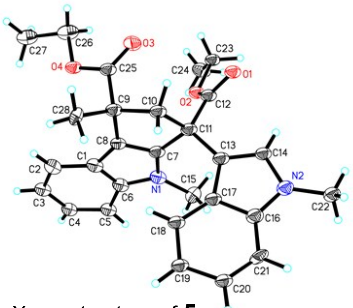

X-ray structure of **5a**

Table 1. Crystal data and structure refinement for 5a.

Identification code	exp_1776
Empirical formula	$C_{28}H_{30}N_2O_4$
Formula weight	458.54
Temperature/K	100.01(10)
Crystal system	monoclinic
Space group	$P2_1$
a/Å	16.179(2)
b/Å	8.8178(15)
c/Å	17.233(2)
α/°	90
β/°	103.819(13)
γ/°	90
Volume/Å^3	2387.3(6)
Z	2
ρ_{calc}g/cm^3	0.638
μ/mm^{-1}	0.043
F(000)	488.0
Crystal size/mm^3	0.13 × 0.12 × 0.11
Radiation	MoKα (λ = 0.71073)
2Θ range for data collection/°	4.868 to 49.98

Index ranges	-15 ≤ h ≤ 19, -8 ≤ k ≤ 10, -20 ≤ l ≤ 20
Reflections collected	11382
Independent reflections	7256 [R_{int} = 0.0473, R_{sigma} = 0.0860]
Data/restraints/parameters	7256/109/174
Goodness-of-fit on F^2	1.070
Final R indexes [I>=2σ (I)]	R_1 = 0.0732, wR_2 = 0.1990
Final R indexes [all data]	R_1 = 0.0787, wR_2 = 0.2059
Largest diff. peak/hole / e $Å^{-3}$	0.51/-0.47
Flack parameter	-0.2(9)

Crystal structure determination of 5a

Crystal Data for $C_{28}H_{30}N_2O_4$ (M =458.54 g/mol): monoclinic, space group $P2_1$ (no. 4), a = 16.179(2) Å, b = 8.8178(15) Å, c = 17.233(2) Å, β = 103.819(13)°, V = 2387.3(6) $Å^3$, Z = 2, T = 100.01(10) K, μ(MoKα) = 0.043 mm^{-1}, $Dcalc$ = 0.638 g/cm^3, 11382 reflections measured (4.868° ≤ 2Θ ≤ 49.98°), 7256 unique (R_{int} = 0.0473, R_{sigma} = 0.0860) which were used in all calculations. The final R_1 was 0.0732 (I > 2σ(I)) and wR_2 was 0.2059 (all data).

5. References

1. (a) A. El-Harairy, B. Lai, L. Vaccaro, M. Li, Y. Gu, *Advanced Synthesis & Catalysis*. 2019, **361**, 3342-3350; b) A. El-Harairy, M. Yue, W. Fan, F. Popowycz, Y. Queneau, M. Li, Y. Gu, *ChemCatChem*, 2019, **11**, 4403-4410.
2. Y. Gu, F. Shi, Y. Deng, *J. Mol. Catal. A: Chem*. 2004, **212**, 71–75.
3. A. Taheri, X. Pan, C. Liu, Y. Gu, *ChemSusChem* 2014, **7**, 2094–2100.

6. NMR Spectra

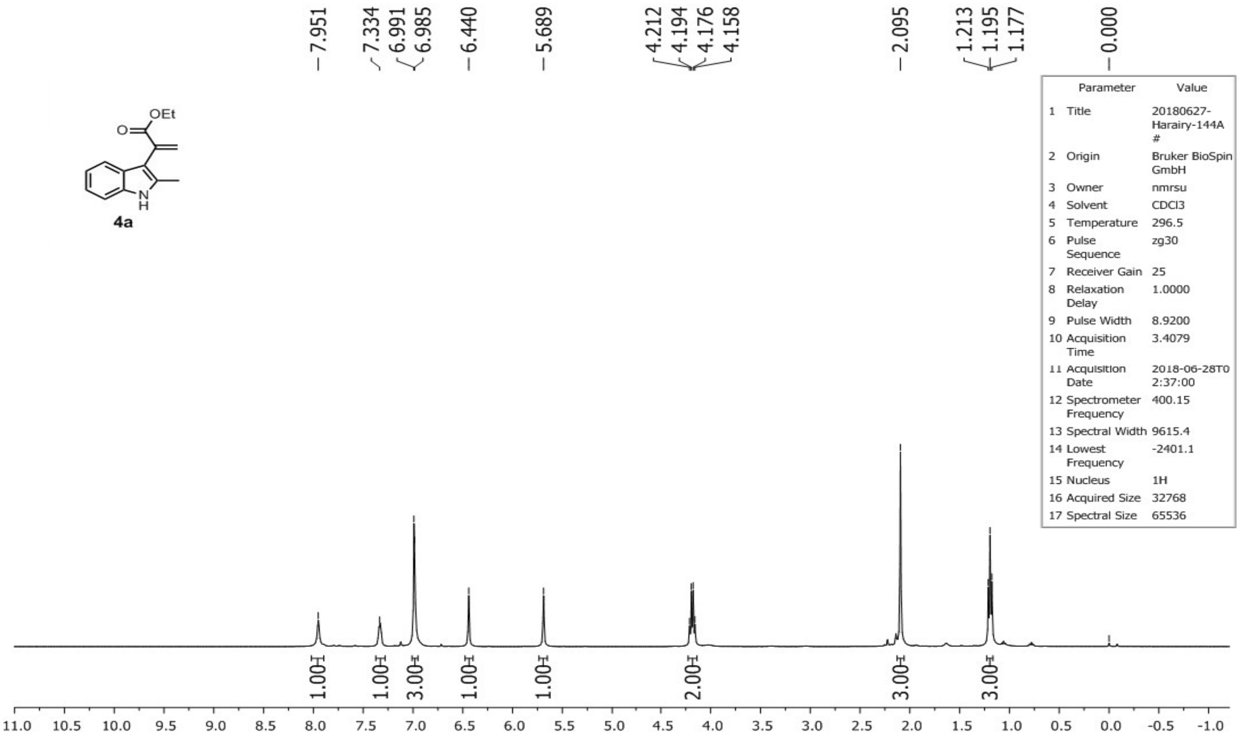

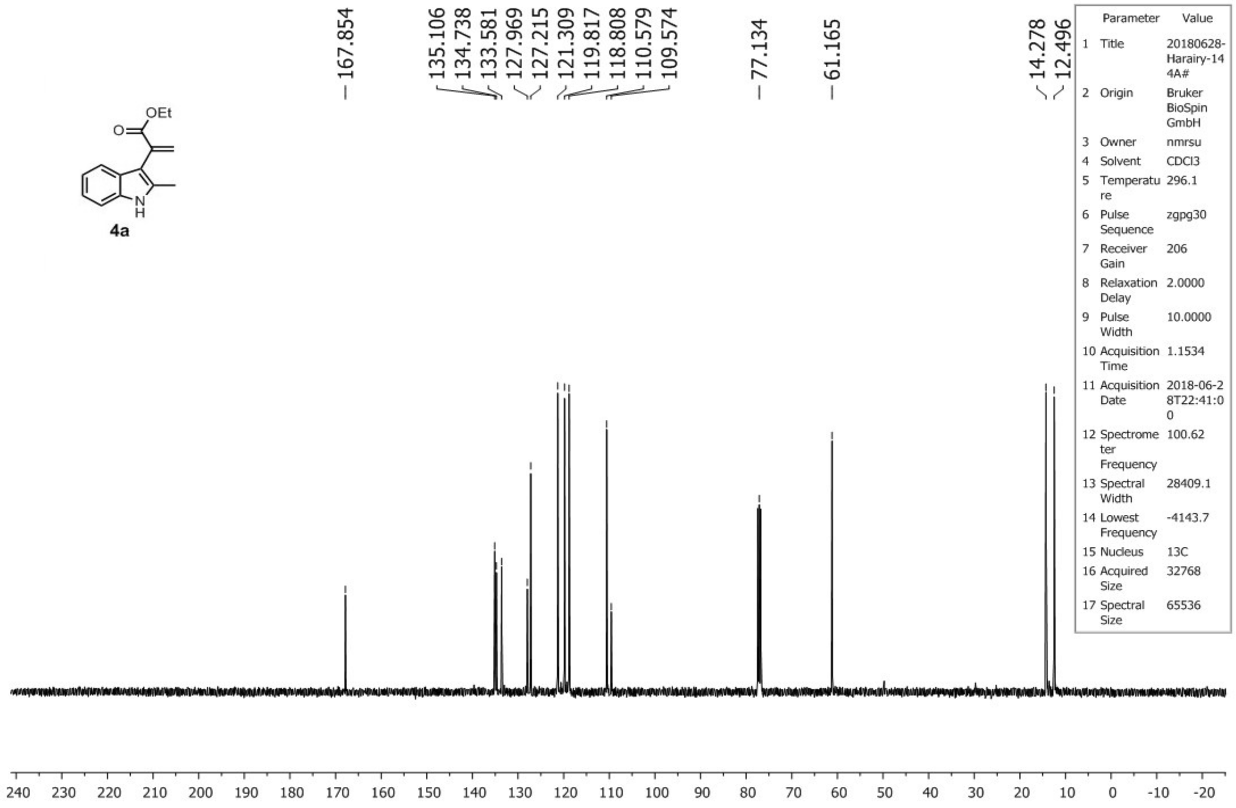

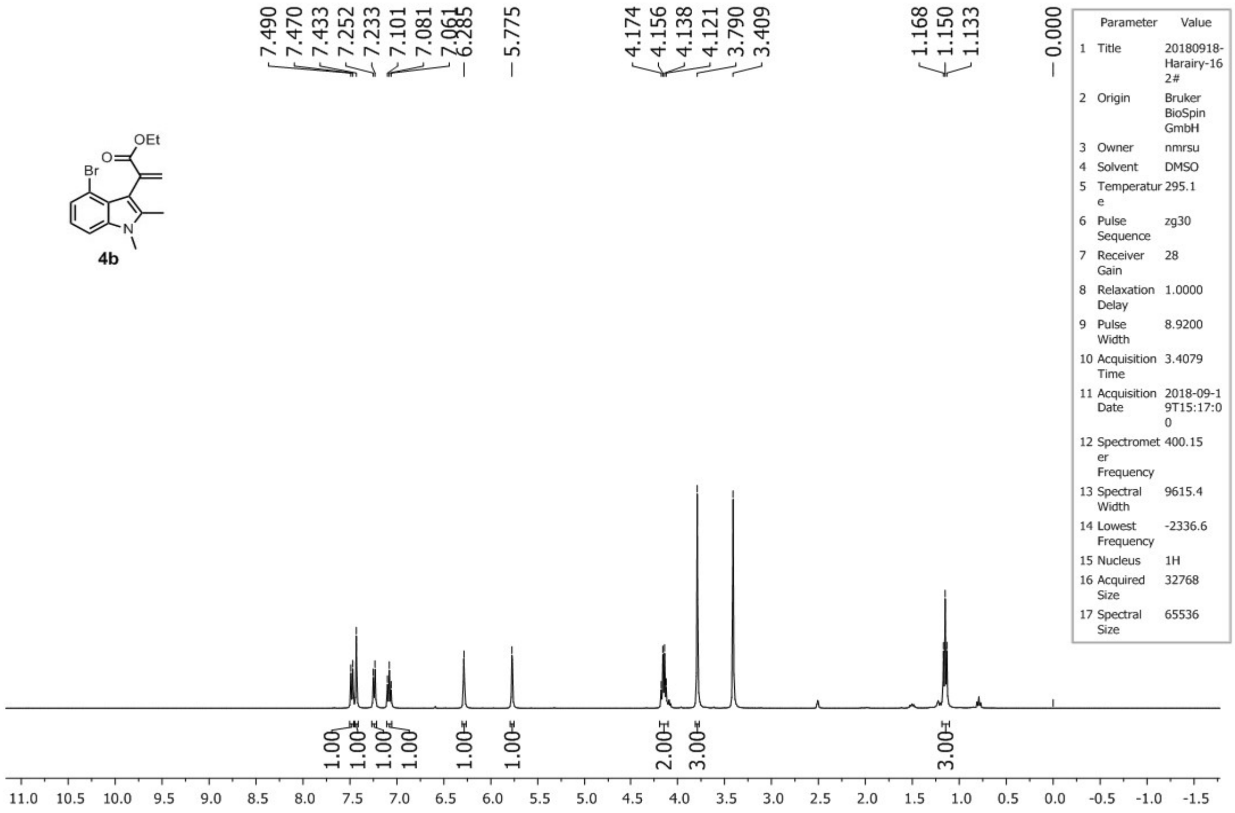

	Parameter	Value
1	Title	20180918-Harairy-162#
2	Origin	Bruker BioSpin GmbH
3	Owner	nmrsu
4	Solvent	DMSO
5	Temperature	295.1
6	Pulse Sequence	zg30
7	Receiver Gain	28
8	Relaxation Delay	1.0000
9	Pulse Width	8.9200
10	Acquisition Time	3.4079
11	Acquisition Date	2018-09-19T15:17:00
12	Spectrometer Frequency	400.15
13	Spectral Width	9615.4
14	Lowest Frequency	-2336.6
15	Nucleus	1H
16	Acquired Size	32768
17	Spectral Size	65536

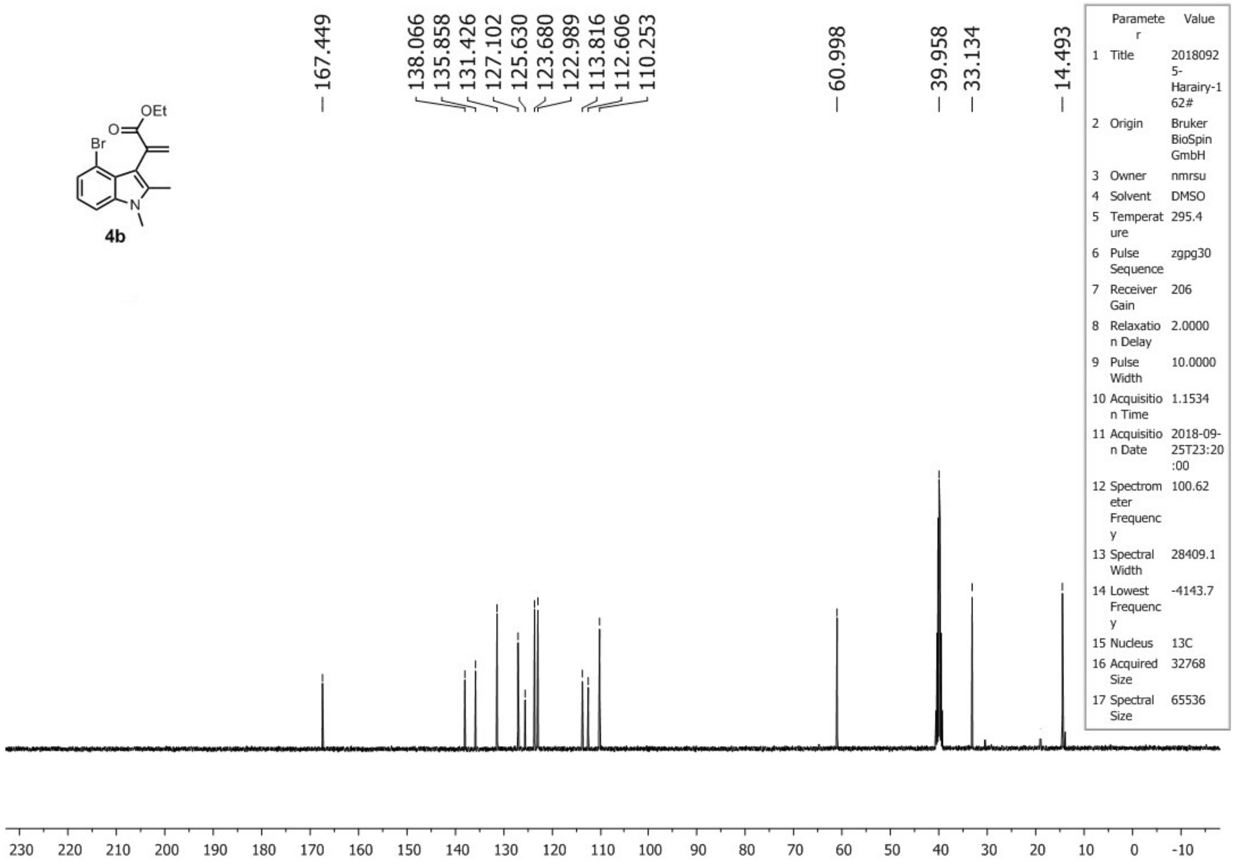

	Parameter	Value
1	Title	20180925-Harairy-162#
2	Origin	Bruker BioSpin GmbH
3	Owner	nmrsu
4	Solvent	DMSO
5	Temperature	295.4
6	Pulse Sequence	zgpg30
7	Receiver Gain	206
8	Relaxation Delay	2.0000
9	Pulse Width	10.0000
10	Acquisition Time	1.1534
11	Acquisition Date	2018-09-25T23:20:00
12	Spectrometer Frequency	100.62
13	Spectral Width	28409.1
14	Lowest Frequency	-4143.7
15	Nucleus	13C
16	Acquired Size	32768
17	Spectral Size	65536

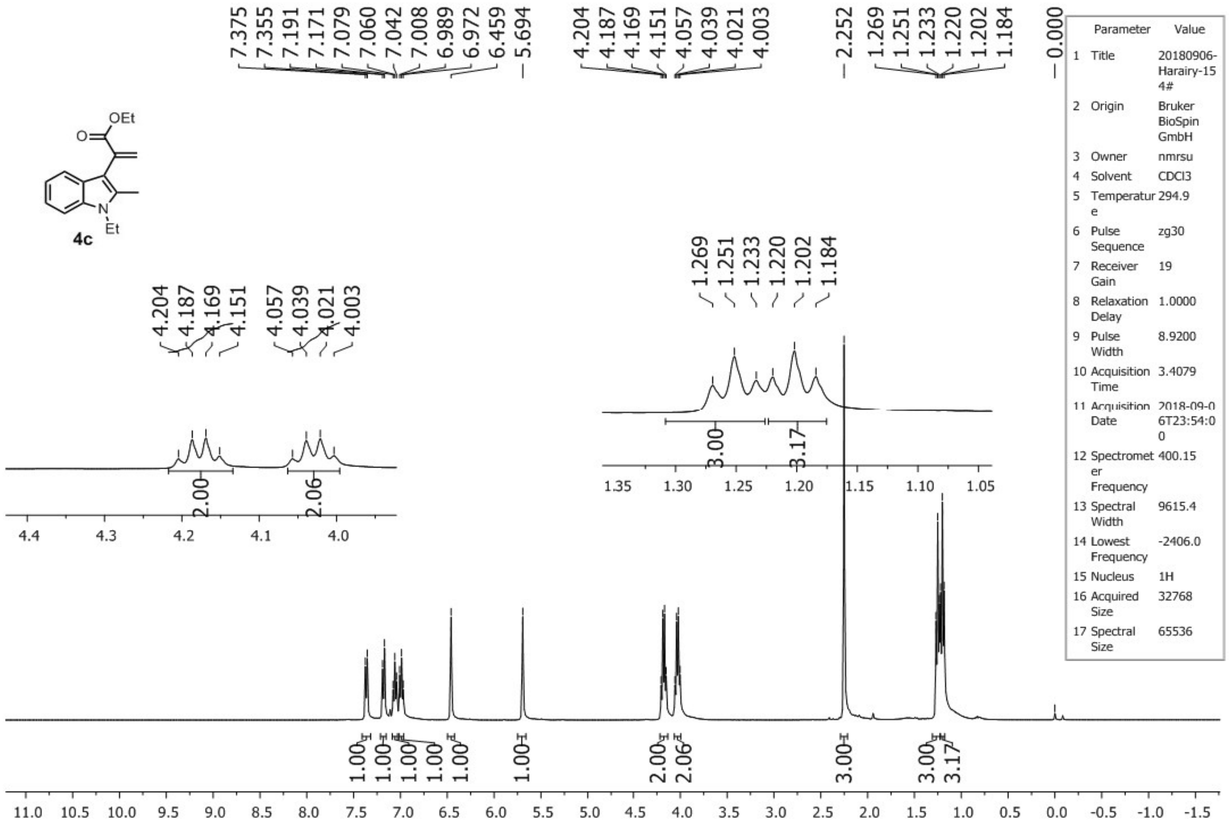
7.375
7.355
7.191
7.171
7.079
7.060
7.042
7.008
6.989
6.972
6.459
5.694
4.204
4.187
4.169
4.151
4.057
4.039
4.021
4.003
2.252
1.269
1.251
1.233
1.220
1.202
1.184
0.000
OEt
O
N
Et
4c
Parameter Value
1 Title 20180906-Harairy-154#
2 Origin Bruker BioSpin GmbH
3 Owner nmrsu
4 Solvent CDCl3
5 Temperature 294.9
6 Pulse Sequence zg30
7 Receiver Gain 19
8 Relaxation Delay 1.0000
9 Pulse Width 8.9200
10 Acquisition Time 3.4079
11 Acquisition Date 2018-09-06T23:54:00
12 Spectrometer Frequency 400.15
13 Spectral Width 9615.4
14 Lowest Frequency -2406.0
15 Nucleus 1H
16 Acquired Size 32768
17 Spectral Size 65536
1.00
1.00
1.00
1.00
1.00
1.00
2.00
2.06
3.00
3.00
3.17

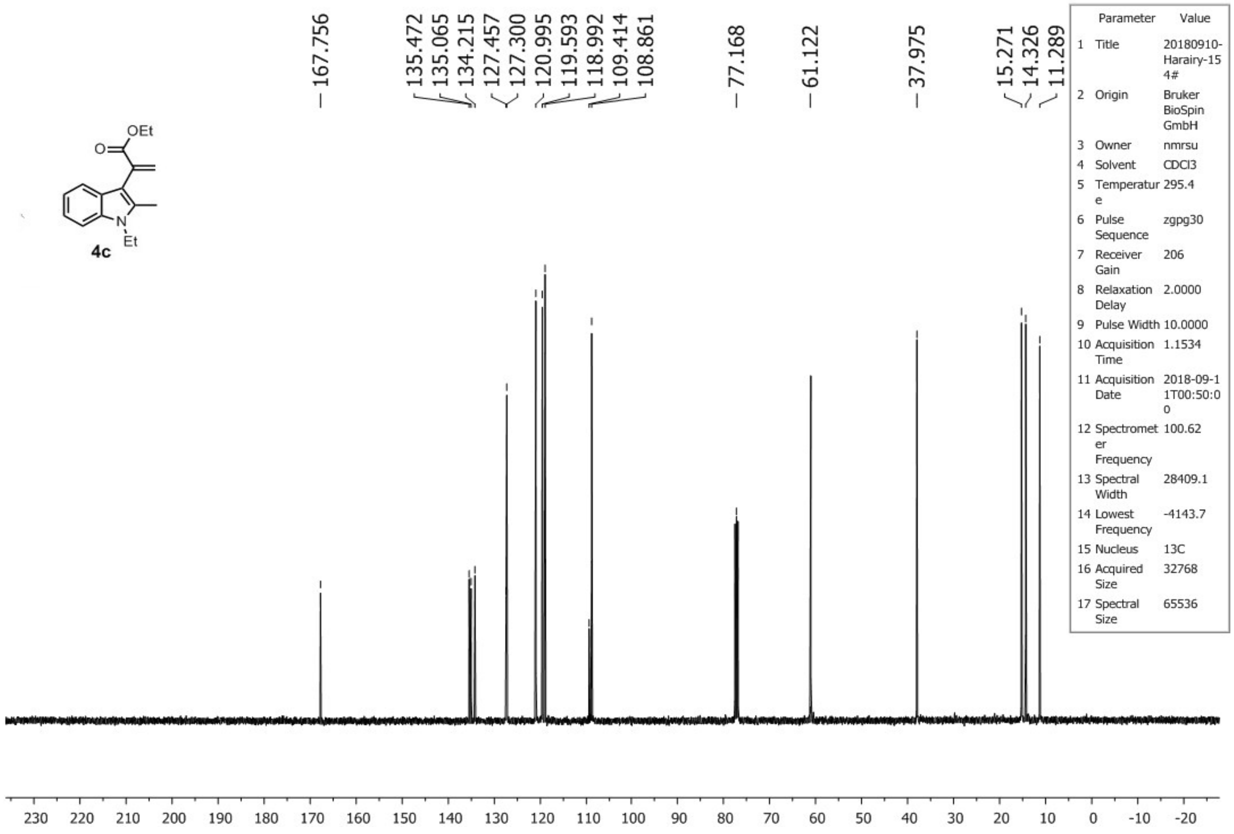
167.756
135.472
135.065
134.215
127.457
127.300
120.995
119.593
118.992
109.414
108.861
77.168
61.122
37.975
15.271
14.326
11.289
OEt
O
N
Et
4c
Parameter Value
1 Title 20180910-Harairy-154#
2 Origin Bruker BioSpin GmbH
3 Owner nmrsu
4 Solvent CDCl3
5 Temperature 295.4
6 Pulse Sequence zgpg30
7 Receiver Gain 206
8 Relaxation Delay 2.0000
9 Pulse Width 10.0000
10 Acquisition Time 1.1534
11 Acquisition Date 2018-09-11T00:50:00
12 Spectrometer Frequency 100.62
13 Spectral Width 28409.1
14 Lowest Frequency -4143.7
15 Nucleus 13C
16 Acquired Size 32768
17 Spectral Size 65536

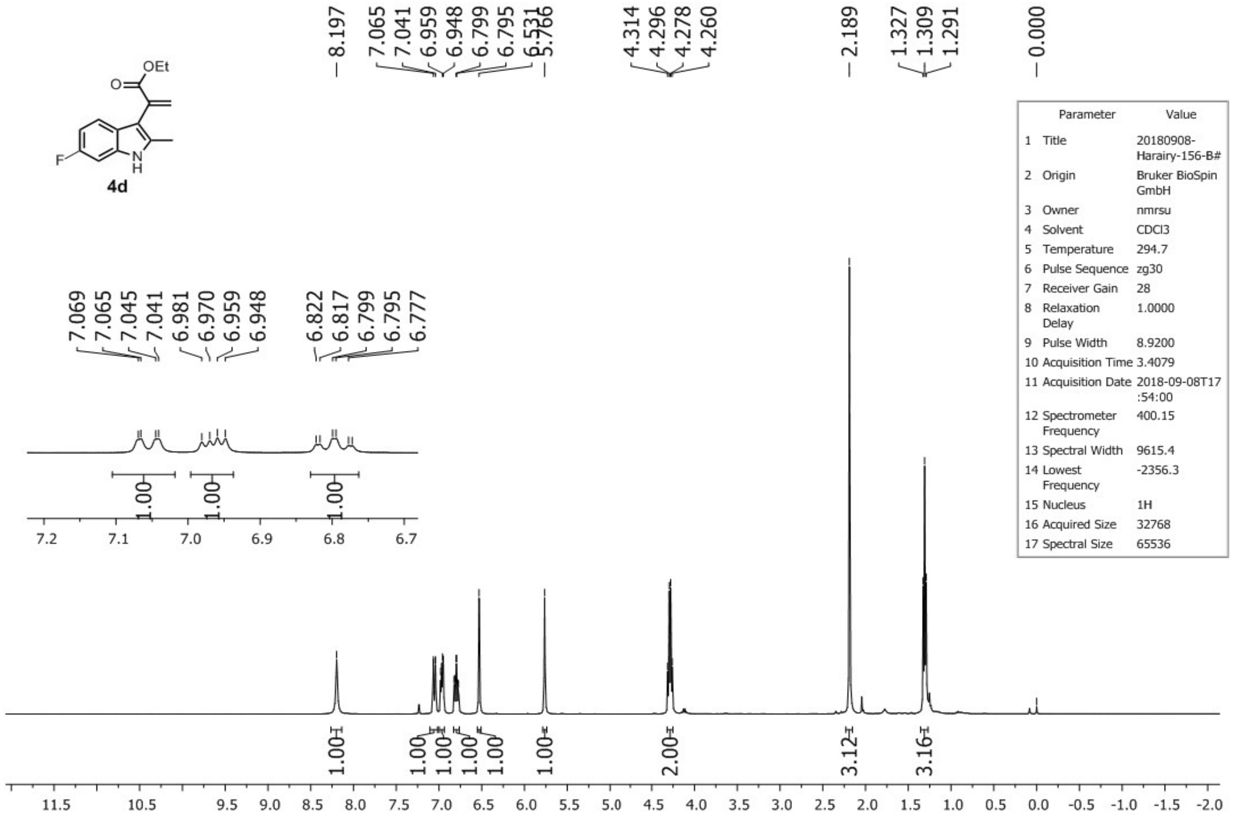
OEt
4d
8.197
7.065 7.041 6.959 6.948 6.799 6.795 6.531 5.766
4.314 4.296 4.278 4.260
2.189
1.327 1.309 1.291
0.000
7.069 7.065 7.045 7.041 6.981 6.970 6.959 6.948
6.822 6.817 6.799 6.795 6.777
Parameter Value
1 Title 20180908-Harairy-156-B#
2 Origin Bruker BioSpin GmbH
3 Owner nmrsu
4 Solvent CDCl3
5 Temperature 294.7
6 Pulse Sequence zg30
7 Receiver Gain 28
8 Relaxation Delay 1.0000
9 Pulse Width 8.9200
10 Acquisition Time 3.4079
11 Acquisition Date 2018-09-08T17:54:00
12 Spectrometer Frequency 400.15
13 Spectral Width 9615.4
14 Lowest Frequency -2356.3
15 Nucleus 1H
16 Acquired Size 32768
17 Spectral Size 65536
1.00 1.00 1.00 1.00 1.00 1.00 1.00 1.00 2.00 3.12 3.16

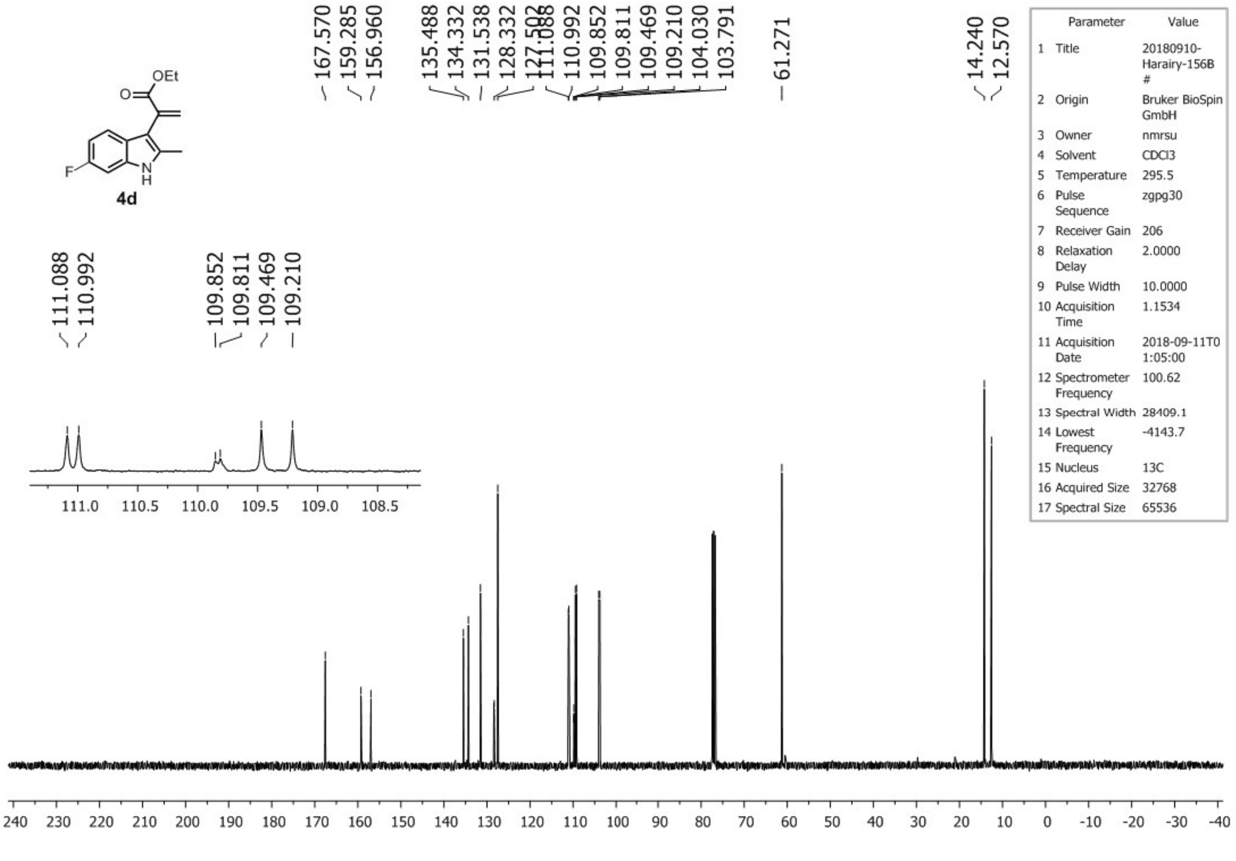
OEt
4d
167.570 159.285 156.960
135.488 134.332 131.538 128.332 127.502 111.088 110.992 109.852 109.811 109.469 109.210 104.030 103.791
61.271
14.240 12.570
111.088 110.992 109.852 109.811 109.469 109.210
Parameter Value
1 Title 20180910-Harairy-156B#
2 Origin Bruker BioSpin GmbH
3 Owner nmrsu
4 Solvent CDCl3
5 Temperature 295.5
6 Pulse Sequence zgpg30
7 Receiver Gain 206
8 Relaxation Delay 2.0000
9 Pulse Width 10.0000
10 Acquisition Time 1.1534
11 Acquisition Date 2018-09-11T01:05:00
12 Spectrometer Frequency 100.62
13 Spectral Width 28409.1
14 Lowest Frequency -4143.7
15 Nucleus 13C
16 Acquired Size 32768
17 Spectral Size 65536

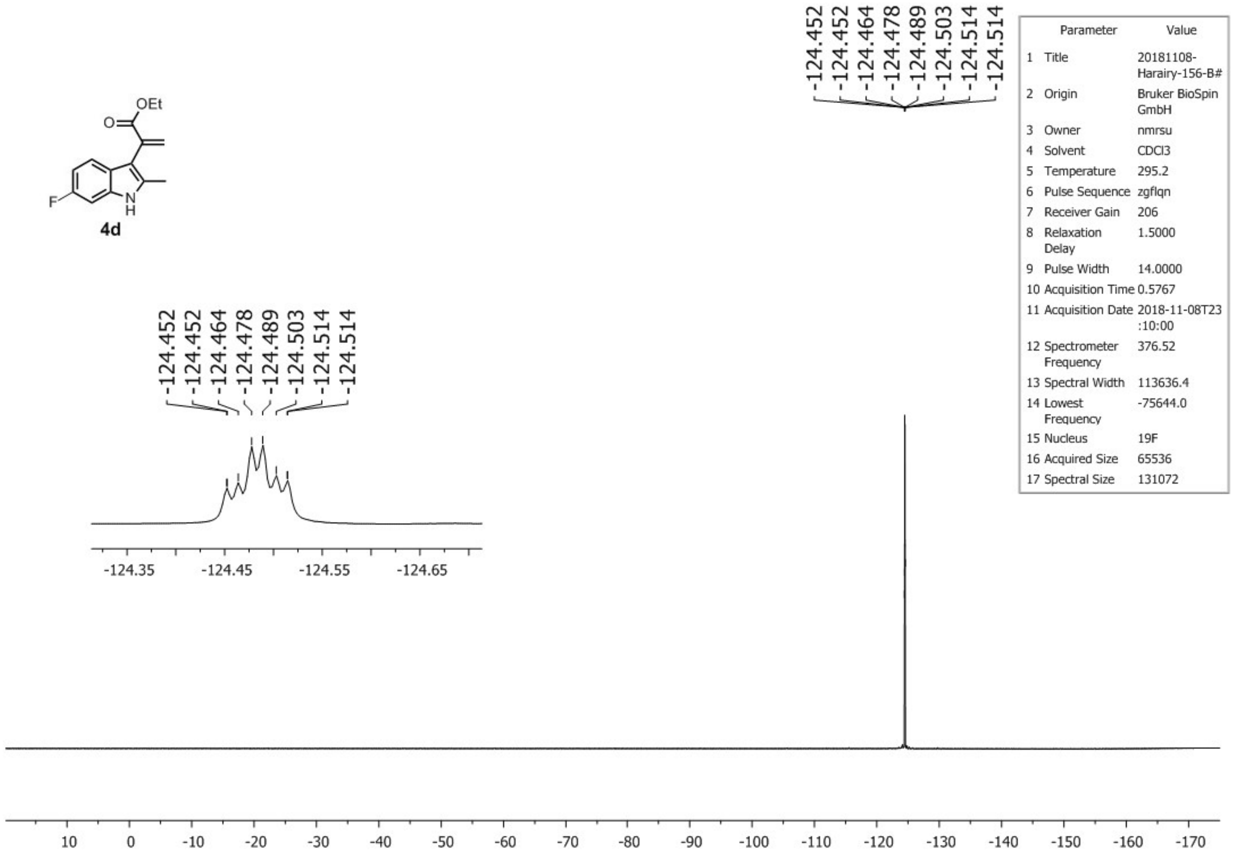
OEt
O
F
N
H
4d
-124.452
-124.452
-124.464
-124.478
-124.489
-124.503
-124.514
-124.514
-124.35
-124.45
-124.55
-124.65
Parameter Value
1 Title 20181108-Harairy-156-B#
2 Origin Bruker BioSpin GmbH
3 Owner nmrsu
4 Solvent CDCl3
5 Temperature 295.2
6 Pulse Sequence zgflqn
7 Receiver Gain 206
8 Relaxation Delay 1.5000
9 Pulse Width 14.0000
10 Acquisition Time 0.5767
11 Acquisition Date 2018-11-08T23:10:00
12 Spectrometer Frequency 376.52
13 Spectral Width 113636.4
14 Lowest Frequency -75644.0
15 Nucleus 19F
16 Acquired Size 65536
17 Spectral Size 131072
10 0 -10 -20 -30 -40 -50 -60 -70 -80 -90 -100 -110 -120 -130 -140 -150 -160 -170

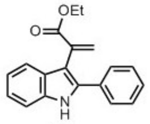

4e

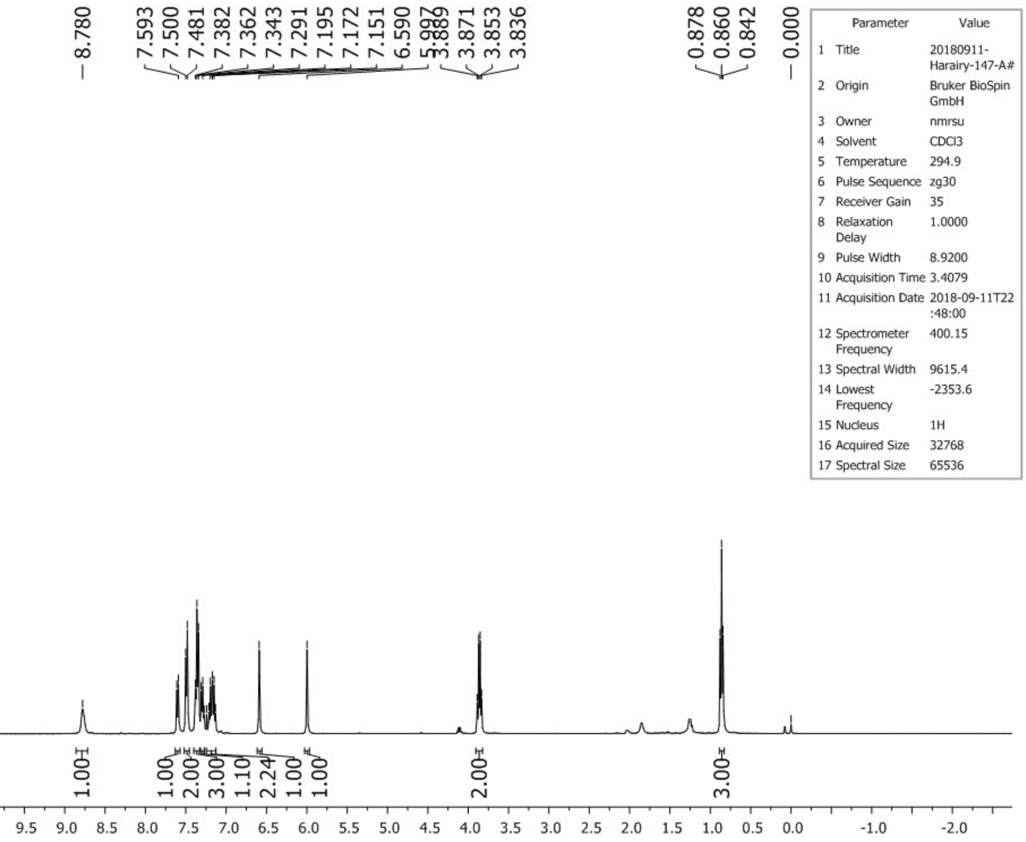

	Parameter	Value
1	Title	20180911-Harairy-147-A#
2	Origin	Bruker BioSpin GmbH
3	Owner	nmrsu
4	Solvent	CDCl3
5	Temperature	294.9
6	Pulse Sequence	zg30
7	Receiver Gain	35
8	Relaxation Delay	1.0000
9	Pulse Width	8.9200
10	Acquisition Time	3.4079
11	Acquisition Date	2018-09-11T22:48:00
12	Spectrometer Frequency	400.15
13	Spectral Width	9615.4
14	Lowest Frequency	-2353.6
15	Nucleus	1H
16	Acquired Size	32768
17	Spectral Size	65536

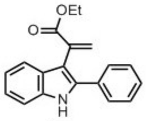

4e

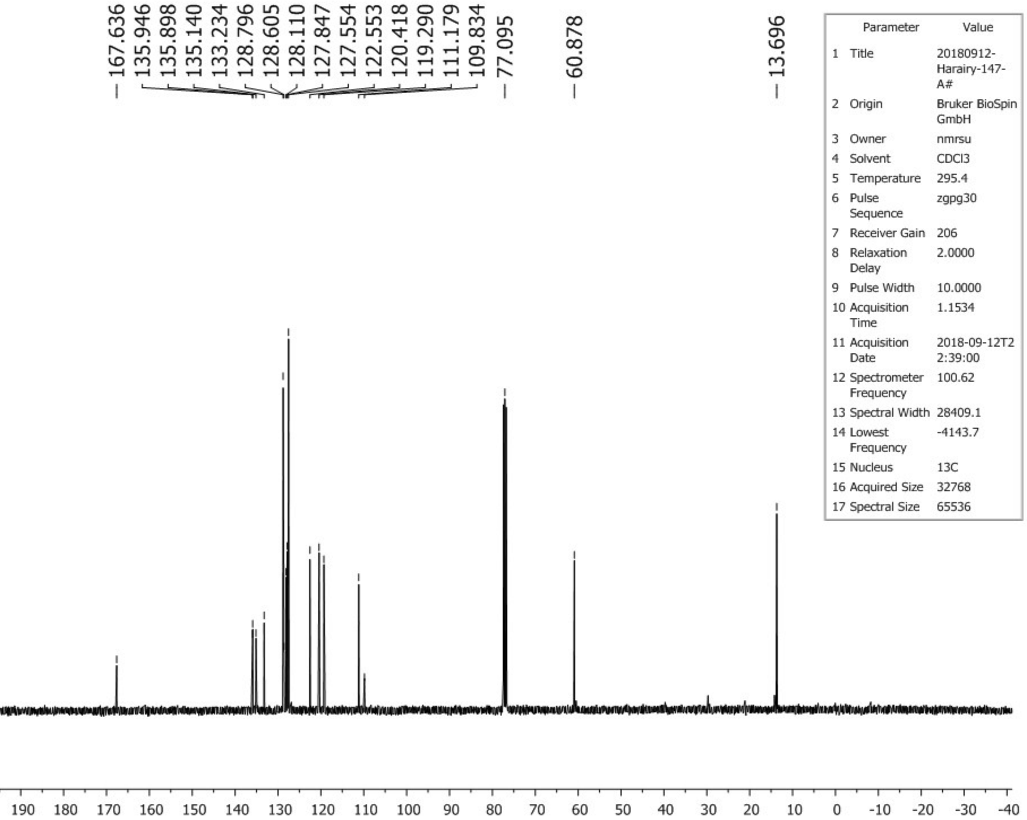

	Parameter	Value
1	Title	20180912-Harairy-147-A#
2	Origin	Bruker BioSpin GmbH
3	Owner	nmrsu
4	Solvent	CDCl3
5	Temperature	295.4
6	Pulse Sequence	zgpg30
7	Receiver Gain	206
8	Relaxation Delay	2.0000
9	Pulse Width	10.0000
10	Acquisition Time	1.1534
11	Acquisition Date	2018-09-12T22:39:00
12	Spectrometer Frequency	100.62
13	Spectral Width	28409.1
14	Lowest Frequency	-4143.7
15	Nucleus	13C
16	Acquired Size	32768
17	Spectral Size	65536

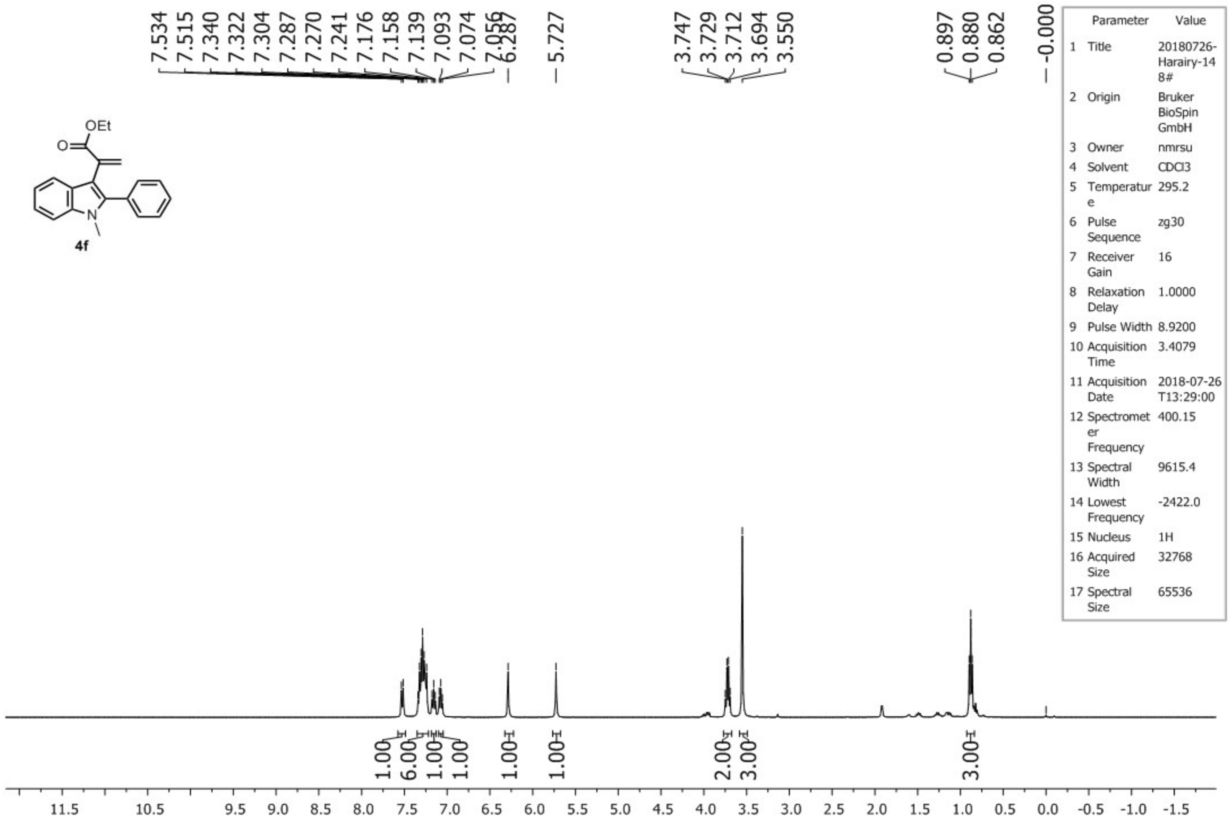

	Parameter	Value
1	Title	20180726-Harairy-148#
2	Origin	Bruker BioSpin GmbH
3	Owner	nmrsu
4	Solvent	CDCl3
5	Temperature	295.2
6	Pulse Sequence	zg30
7	Receiver Gain	16
8	Relaxation Delay	1.0000
9	Pulse Width	8.9200
10	Acquisition Time	3.4079
11	Acquisition Date	2018-07-26T13:29:00
12	Spectrometer Frequency	400.15
13	Spectral Width	9615.4
14	Lowest Frequency	-2422.0
15	Nucleus	1H
16	Acquired Size	32768
17	Spectral Size	65536

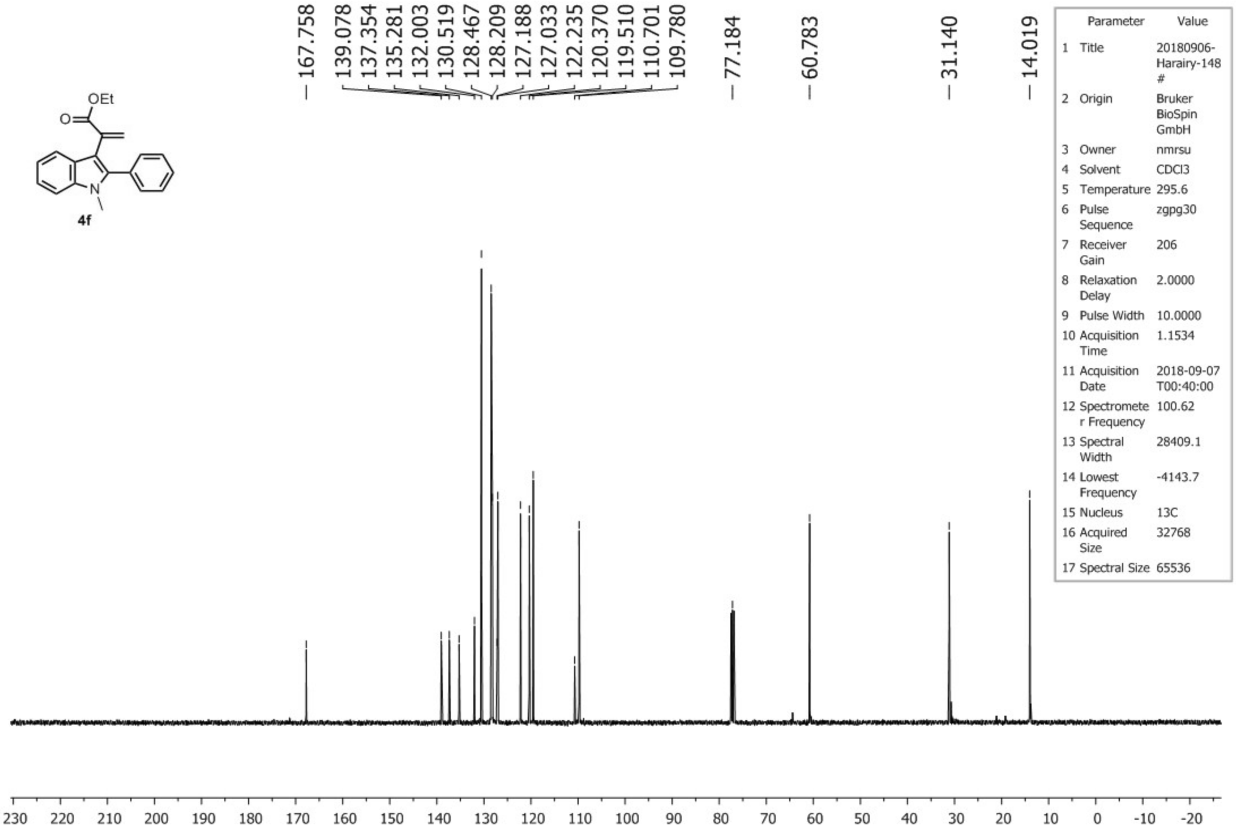

	Parameter	Value
1	Title	20180906-Harairy-148#
2	Origin	Bruker BioSpin GmbH
3	Owner	nmrsu
4	Solvent	CDCl3
5	Temperature	295.6
6	Pulse Sequence	zgpg30
7	Receiver Gain	206
8	Relaxation Delay	2.0000
9	Pulse Width	10.0000
10	Acquisition Time	1.1534
11	Acquisition Date	2018-09-07T00:40:00
12	Spectrometer Frequency	100.62
13	Spectral Width	28409.1
14	Lowest Frequency	-4143.7
15	Nucleus	13C
16	Acquired Size	32768
17	Spectral Size	65536

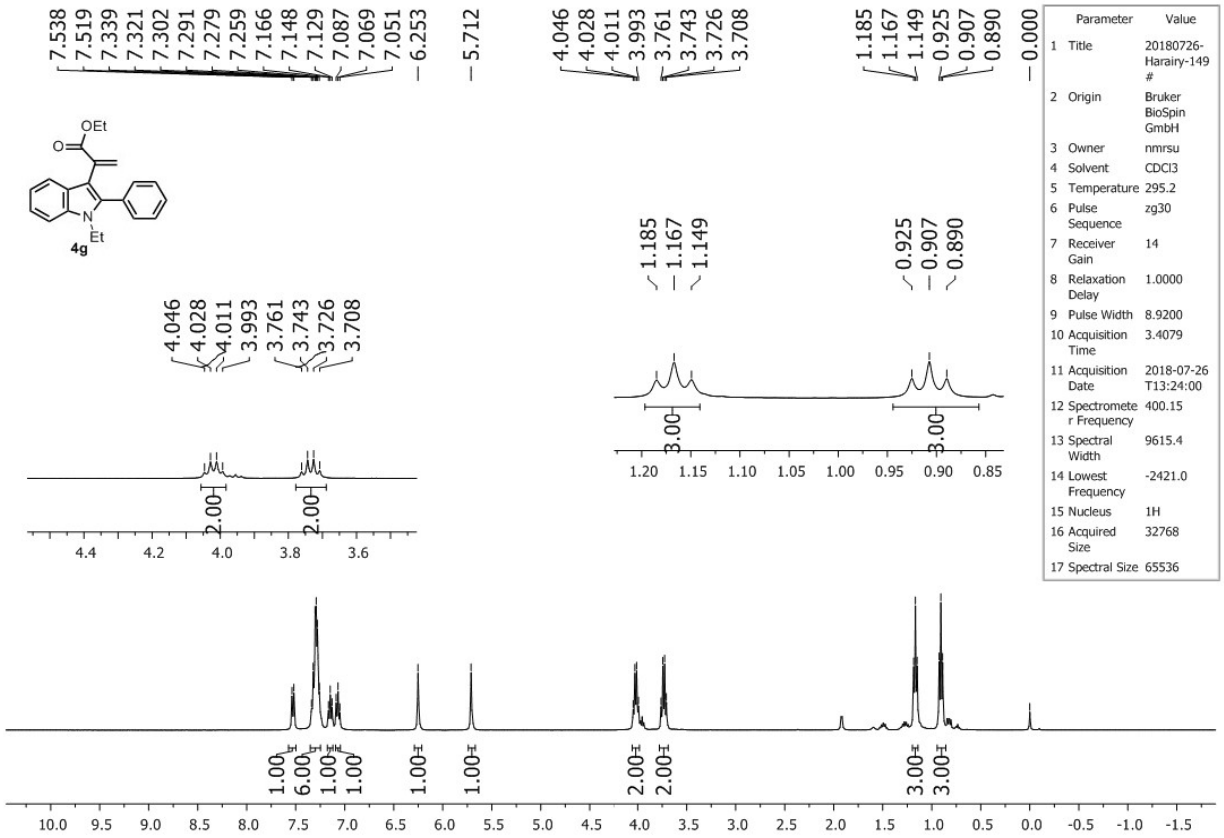
7.538 7.519 7.339 7.321 7.302 7.291 7.279 7.259 7.166 7.148 7.129 7.087 7.069 7.051 6.253 5.712 4.046 4.028 4.011 3.993 3.761 3.743 3.726 3.708 1.185 1.167 1.149 0.925 0.907 0.890 0.000
OEt
O
N
Et
4g
4.046 4.028 4.011 3.993 3.761 3.743 3.726 3.708
2.00 2.00
4.4 4.2 4.0 3.8 3.6
1.185 1.167 1.149 0.925 0.907 0.890
3.00 3.00
1.20 1.15 1.10 1.05 1.00 0.95 0.90 0.85
1.00 6.00 1.00 1.00 1.00 1.00 2.00 2.00 3.00 3.00
10.0 9.5 9.0 8.5 8.0 7.5 7.0 6.5 6.0 5.5 5.0 4.5 4.0 3.5 3.0 2.5 2.0 1.5 1.0 0.5 0.0 -0.5 -1.0 -1.5
Parameter | Value
1 Title | 20180726-Harairy-149 #
2 Origin | Bruker BioSpin GmbH
3 Owner | nmrsu
4 Solvent | CDCl3
5 Temperature | 295.2
6 Pulse Sequence | zg30
7 Receiver Gain | 14
8 Relaxation Delay | 1.0000
9 Pulse Width | 8.9200
10 Acquisition Time | 3.4079
11 Acquisition Date | 2018-07-26 T13:24:00
12 Spectrometer Frequency | 400.15
13 Spectral Width | 9615.4
14 Lowest Frequency | -2421.0
15 Nucleus | 1H
16 Acquired Size | 32768
17 Spectral Size | 65536

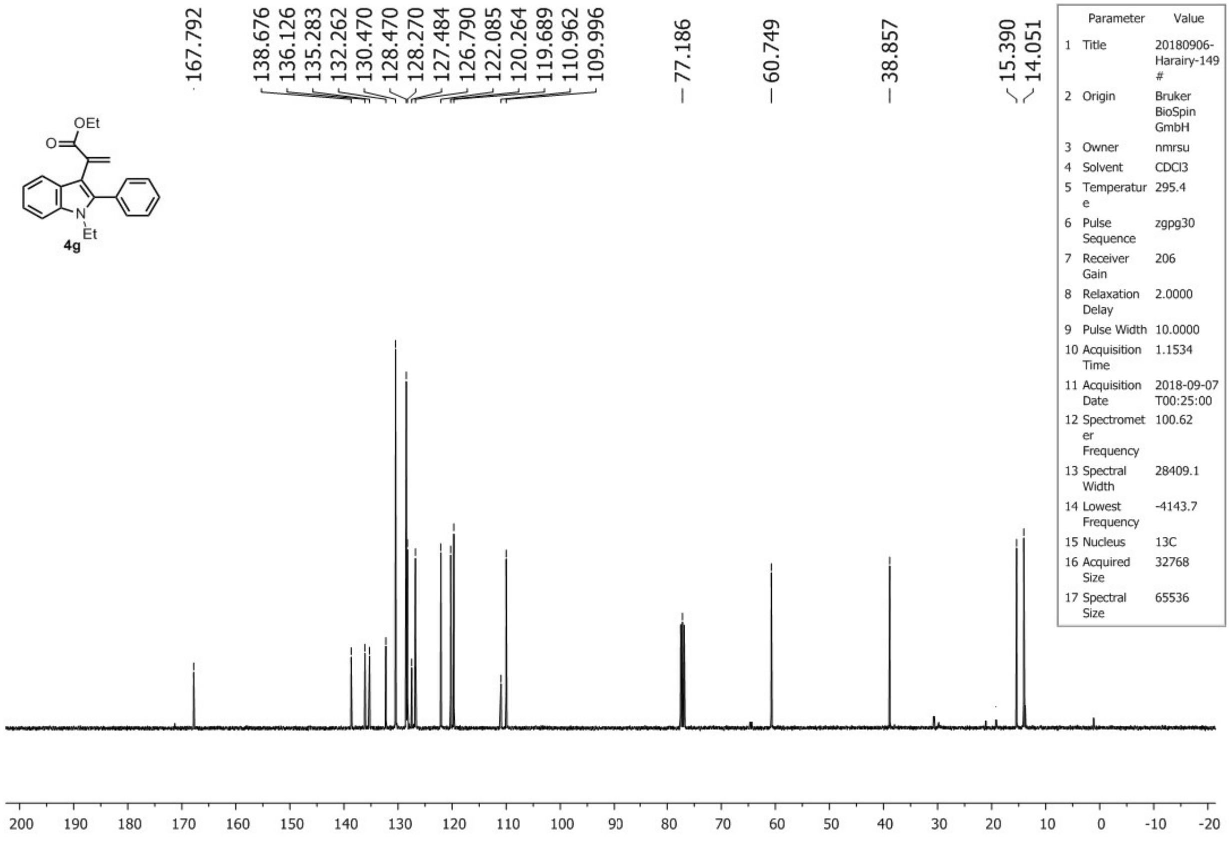
167.792 138.676 136.126 135.283 132.262 130.470 128.470 128.270 127.484 126.790 122.085 120.264 119.689 110.962 109.996 77.186 60.749 38.857 15.390 14.051
OEt
O
N
Et
4g
200 190 180 170 160 150 140 130 120 110 100 90 80 70 60 50 40 30 20 10 0 -10 -20
Parameter | Value
1 Title | 20180906-Harairy-149 #
2 Origin | Bruker BioSpin GmbH
3 Owner | nmrsu
4 Solvent | CDCl3
5 Temperature | 295.4
6 Pulse Sequence | zgpg30
7 Receiver Gain | 206
8 Relaxation Delay | 2.0000
9 Pulse Width | 10.0000
10 Acquisition Time | 1.1534
11 Acquisition Date | 2018-09-07 T00:25:00
12 Spectrometer Frequency | 100.62
13 Spectral Width | 28409.1
14 Lowest Frequency | -4143.7
15 Nucleus | 13C
16 Acquired Size | 32768
17 Spectral Size | 65536

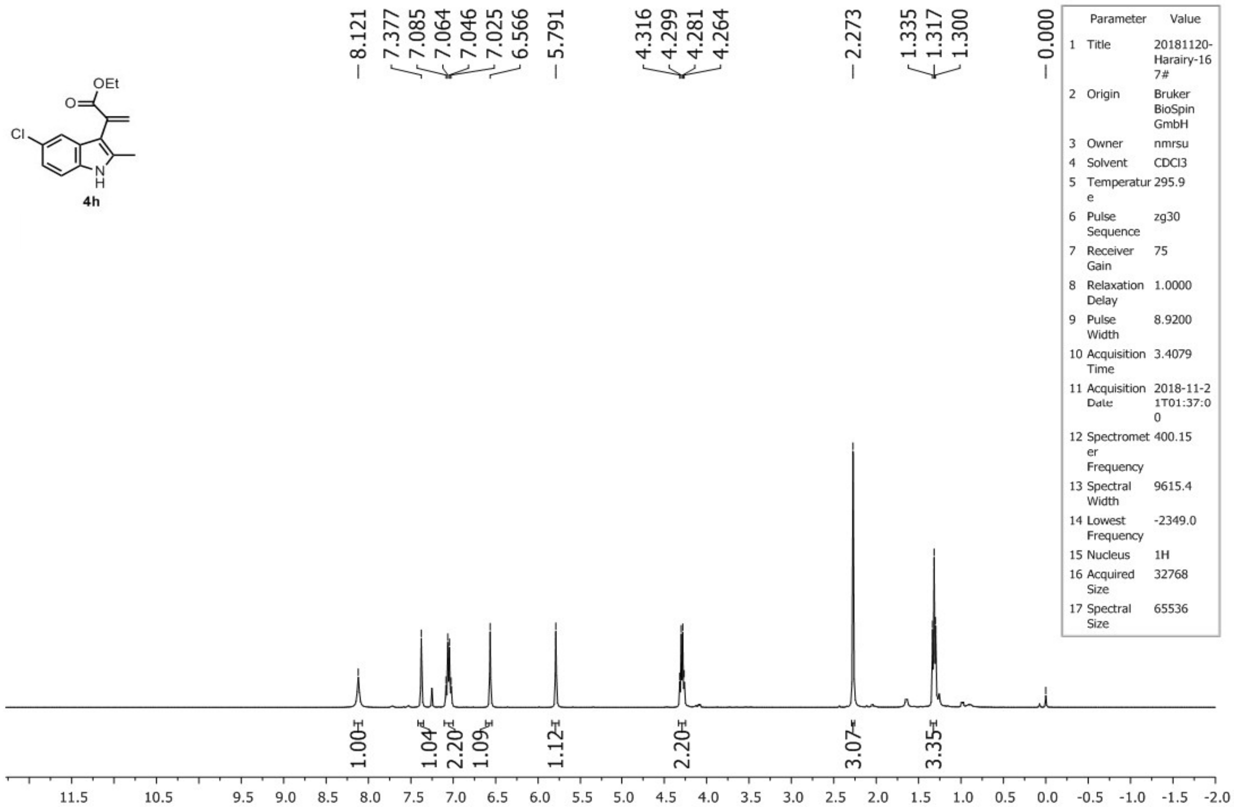

	Parameter	Value
1	Title	20181120-Harairy-167#
2	Origin	Bruker BioSpin GmbH
3	Owner	nmrsu
4	Solvent	CDCl3
5	Temperature	295.9
6	Pulse Sequence	zg30
7	Receiver Gain	75
8	Relaxation Delay	1.0000
9	Pulse Width	8.9200
10	Acquisition Time	3.4079
11	Acquisition Date	2018-11-21T01:37:00
12	Spectrometer Frequency	400.15
13	Spectral Width	9615.4
14	Lowest Frequency	-2349.0
15	Nucleus	1H
16	Acquired Size	32768
17	Spectral Size	65536

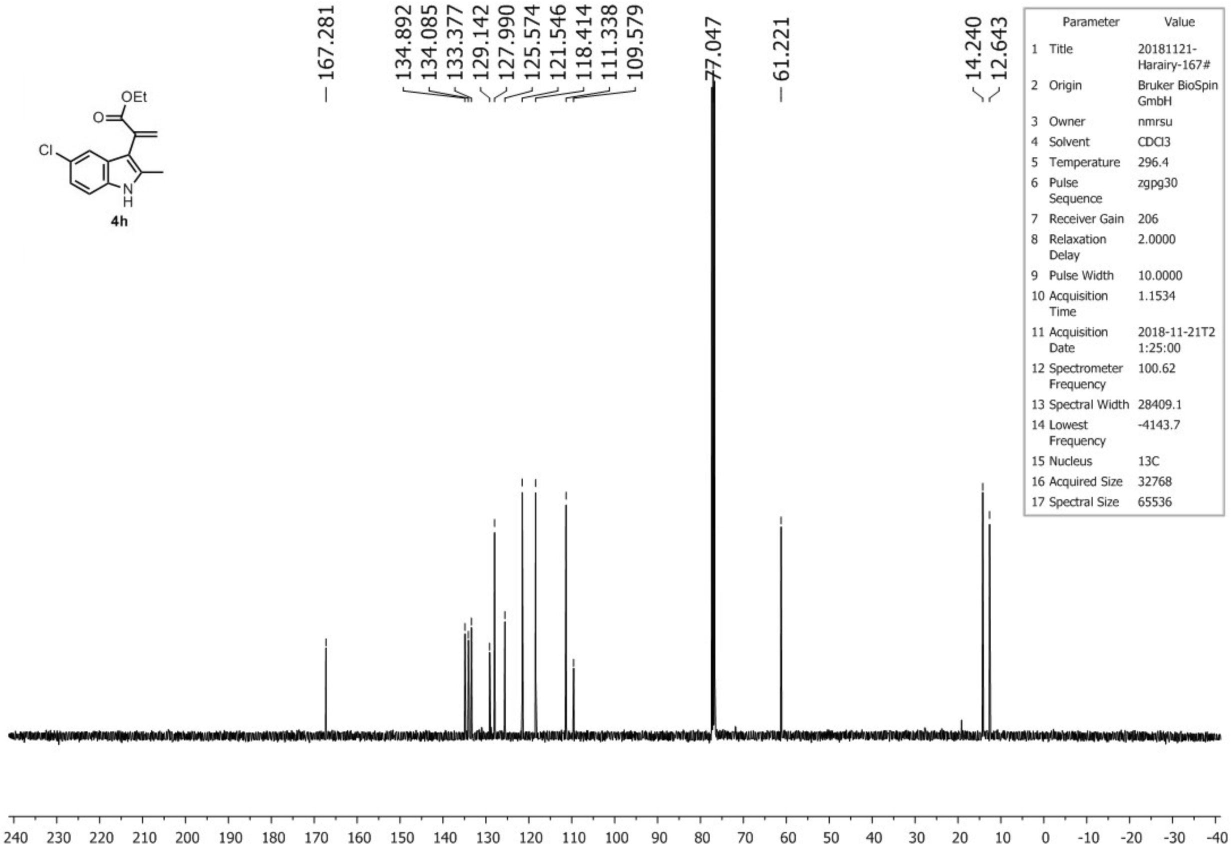

	Parameter	Value
1	Title	20181121-Harairy-167#
2	Origin	Bruker BioSpin GmbH
3	Owner	nmrsu
4	Solvent	CDCl3
5	Temperature	296.4
6	Pulse Sequence	zgpg30
7	Receiver Gain	206
8	Relaxation Delay	2.0000
9	Pulse Width	10.0000
10	Acquisition Time	1.1534
11	Acquisition Date	2018-11-21T21:25:00
12	Spectrometer Frequency	100.62
13	Spectral Width	28409.1
14	Lowest Frequency	-4143.7
15	Nucleus	13C
16	Acquired Size	32768
17	Spectral Size	65536

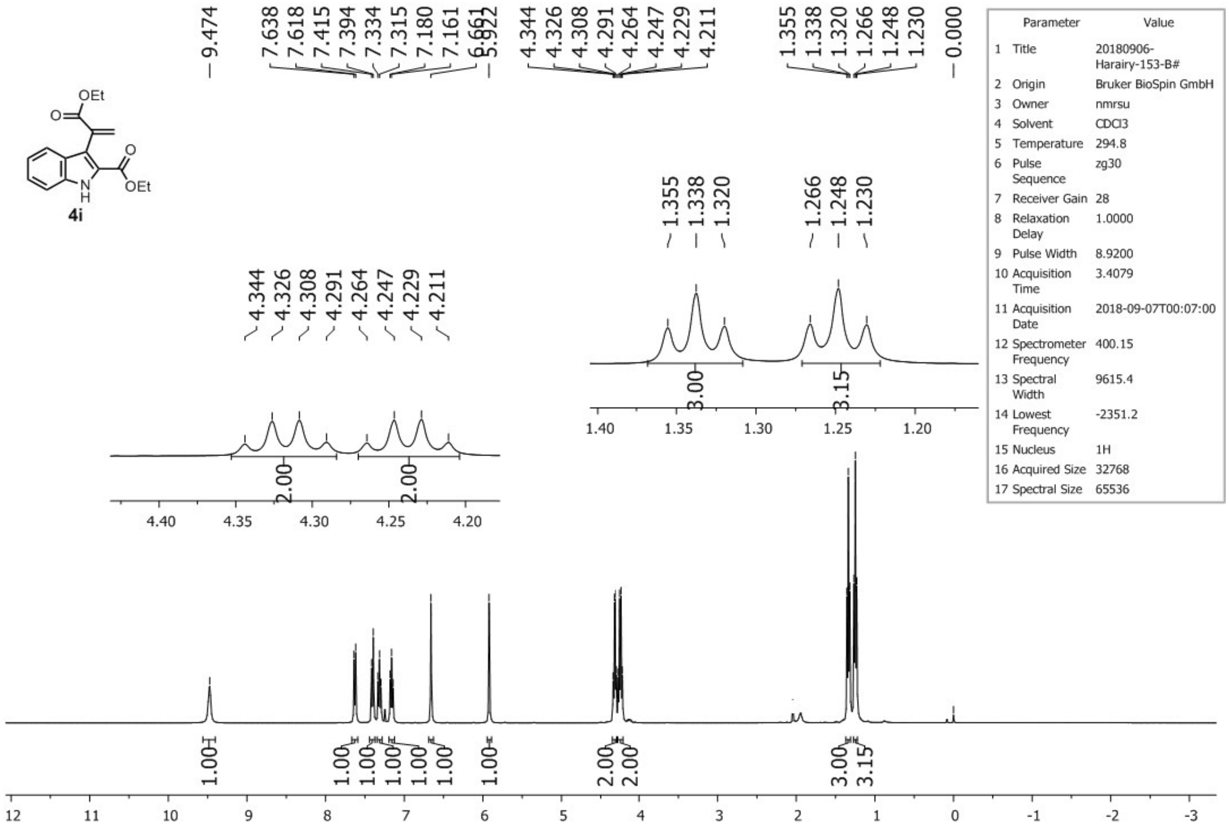

	Parameter	Value
1	Title	20180906-Harairy-153-B#
2	Origin	Bruker BioSpin GmbH
3	Owner	nmrsu
4	Solvent	CDCl3
5	Temperature	294.8
6	Pulse Sequence	zg30
7	Receiver Gain	28
8	Relaxation Delay	1.0000
9	Pulse Width	8.9200
10	Acquisition Time	3.4079
11	Acquisition Date	2018-09-07T00:07:00
12	Spectrometer Frequency	400.15
13	Spectral Width	9615.4
14	Lowest Frequency	-2351.2
15	Nucleus	1H
16	Acquired Size	32768
17	Spectral Size	65536

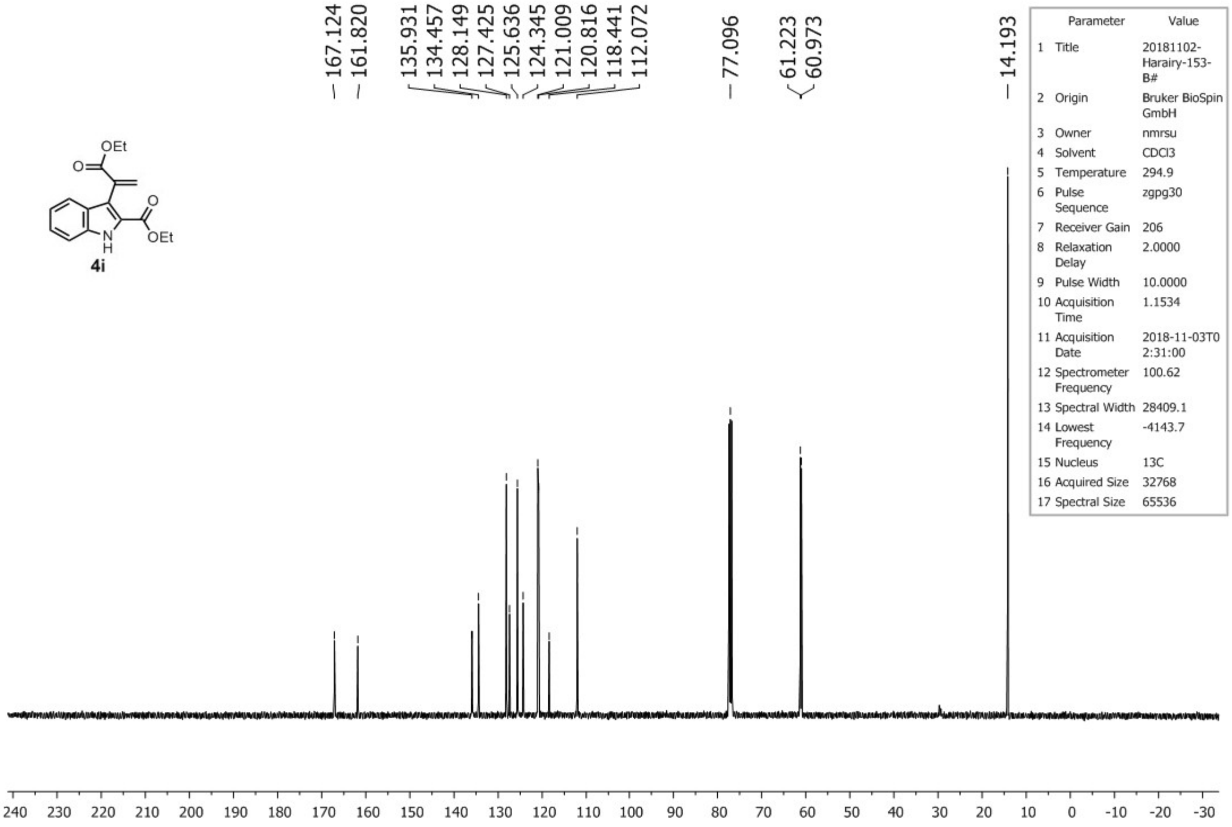

	Parameter	Value
1	Title	20181102-Harairy-153-B#
2	Origin	Bruker BioSpin GmbH
3	Owner	nmrsu
4	Solvent	CDCl3
5	Temperature	294.9
6	Pulse Sequence	zgpg30
7	Receiver Gain	206
8	Relaxation Delay	2.0000
9	Pulse Width	10.0000
10	Acquisition Time	1.1534
11	Acquisition Date	2018-11-03T02:31:00
12	Spectrometer Frequency	100.62
13	Spectral Width	28409.1
14	Lowest Frequency	-4143.7
15	Nucleus	13C
16	Acquired Size	32768
17	Spectral Size	65536

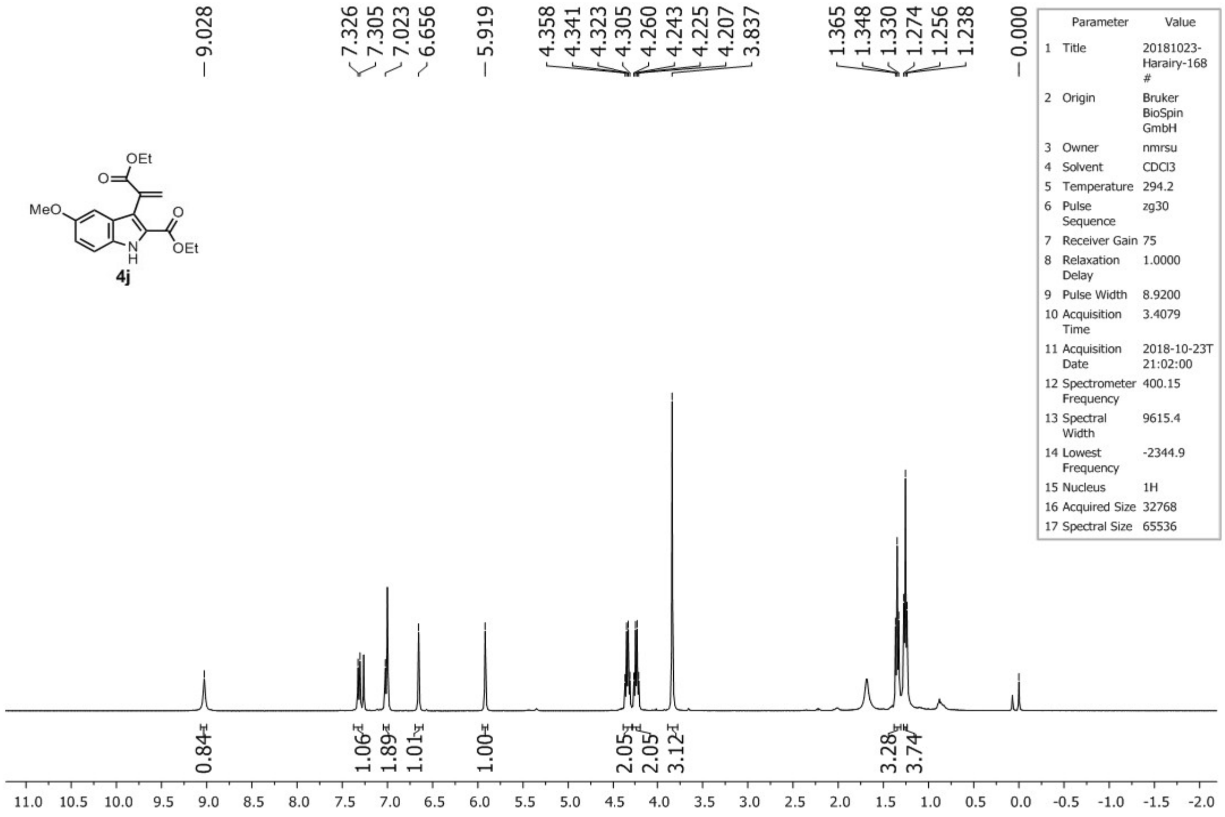
9.028
7.326
7.305
7.023
6.656
5.919
4.358
4.341
4.323
4.305
4.260
4.243
4.225
4.207
3.837
1.365
1.348
1.330
1.274
1.256
1.238
0.000
OEt
O
MeO
O
N
H
OEt
4j
0.84
1.06
1.89
1.01
1.00
2.05
2.05
3.12
3.28
3.74
11.0 10.5 10.0 9.5 9.0 8.5 8.0 7.5 7.0 6.5 6.0 5.5 5.0 4.5 4.0 3.5 3.0 2.5 2.0 1.5 1.0 0.5 0.0 -0.5 -1.0 -1.5 -2.0
Parameter Value
1 Title 20181023-Harairy-168#
2 Origin Bruker BioSpin GmbH
3 Owner nmrsu
4 Solvent CDCl3
5 Temperature 294.2
6 Pulse Sequence zg30
7 Receiver Gain 75
8 Relaxation Delay 1.0000
9 Pulse Width 8.9200
10 Acquisition Time 3.4079
11 Acquisition Date 2018-10-23T21:02:00
12 Spectrometer Frequency 400.15
13 Spectral Width 9615.4
14 Lowest Frequency -2344.9
15 Nucleus 1H
16 Acquired Size 32768
17 Spectral Size 65536

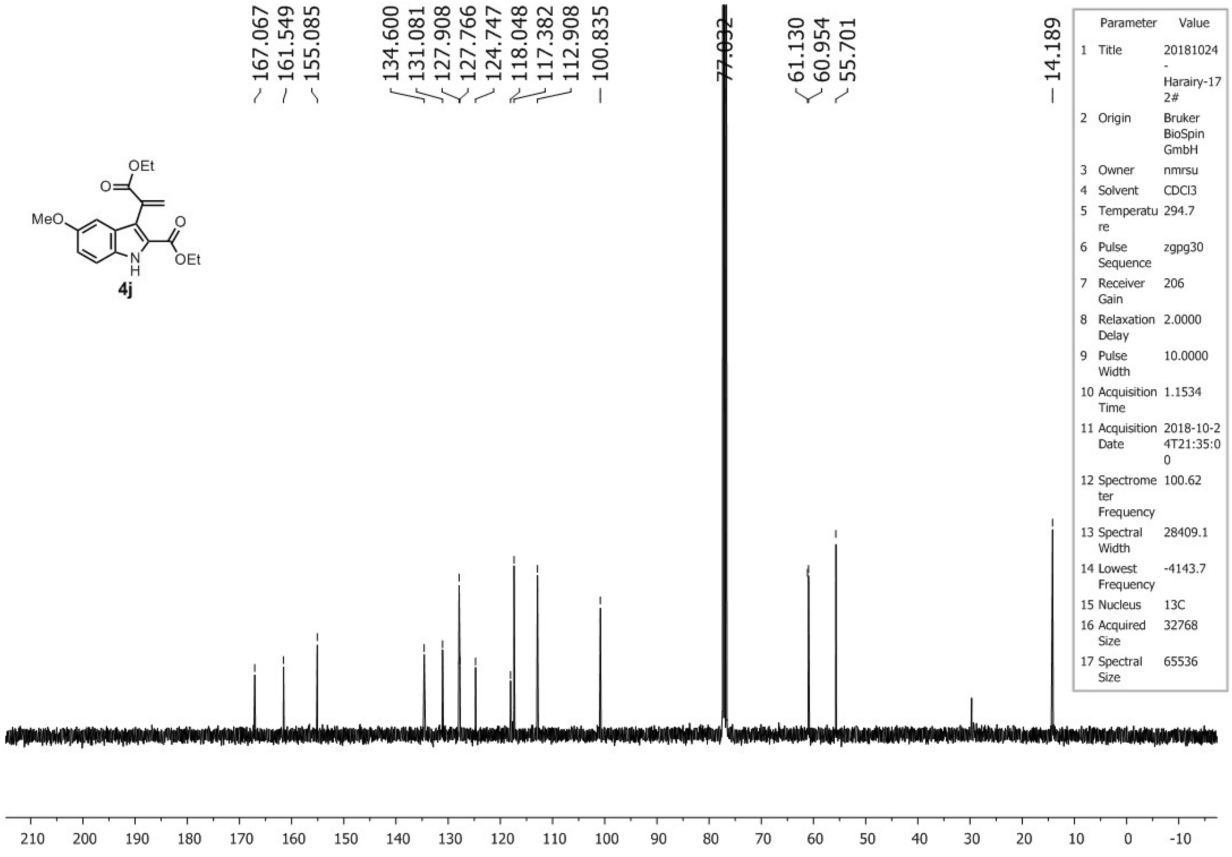
167.067
161.549
155.085
134.600
131.081
127.908
127.766
124.747
118.048
117.382
112.908
100.835
77.032
61.130
60.954
55.701
14.189
OEt
O
MeO
O
N
H
OEt
4j
210 200 190 180 170 160 150 140 130 120 110 100 90 80 70 60 50 40 30 20 10 0 -10
Parameter Value
1 Title 20181024-Harairy-172#
2 Origin Bruker BioSpin GmbH
3 Owner nmrsu
4 Solvent CDCl3
5 Temperature 294.7
6 Pulse Sequence zgpg30
7 Receiver Gain 206
8 Relaxation Delay 2.0000
9 Pulse Width 10.0000
10 Acquisition Time 1.1534
11 Acquisition Date 2018-10-24T21:35:00
12 Spectrometer Frequency 100.62
13 Spectral Width 28409.1
14 Lowest Frequency -4143.7
15 Nucleus 13C
16 Acquired Size 32768
17 Spectral Size 65536

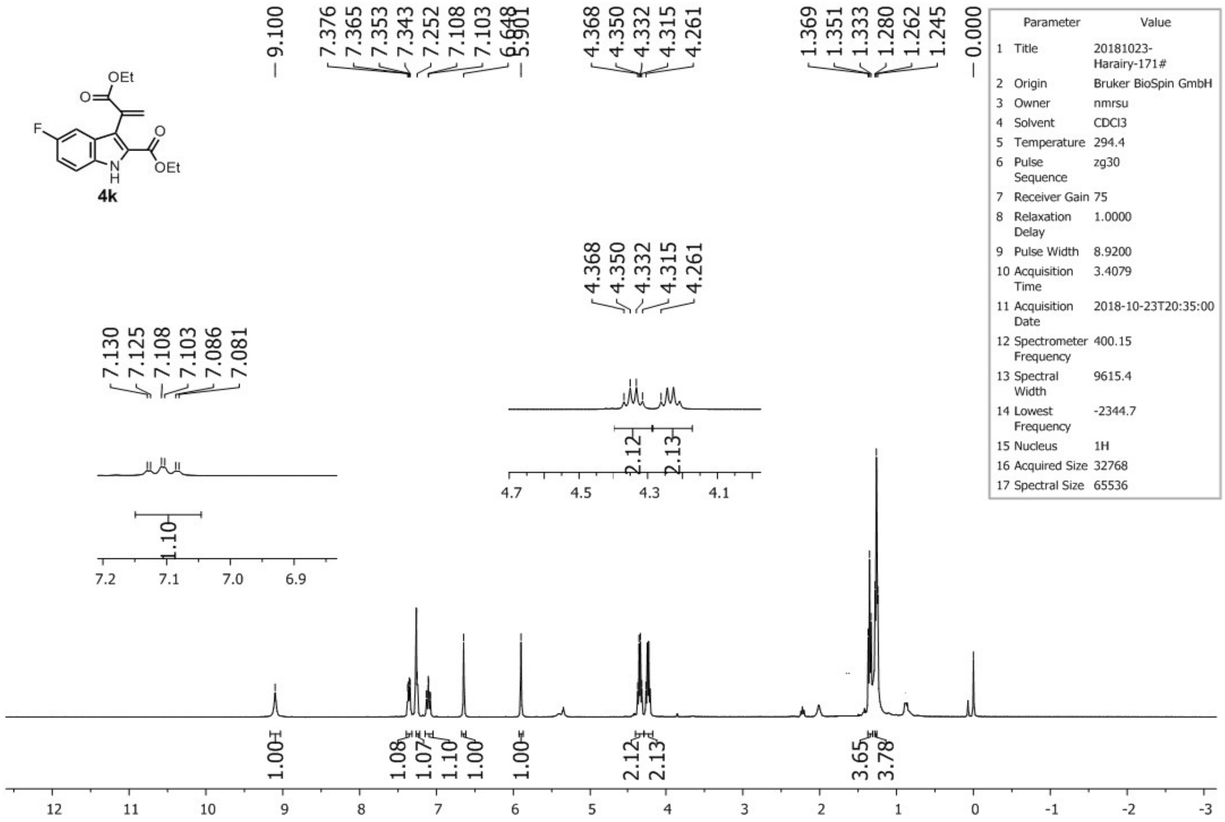

9.100
7.376 7.365 7.353 7.343 7.252 7.108 7.103 6.648 5.901
4.368 4.350 4.332 4.315 4.261
1.369 1.351 1.333 1.280 1.262 1.245
0.000
4k
7.130 7.125 7.108 7.103 7.086 7.081
1.10
2.12 2.13
1.00 1.08 1.07 1.10 1.00 1.00 2.12 2.13 3.65 3.78
Parameter / Value
1 Title 20181023-Harairy-171#
2 Origin Bruker BioSpin GmbH
3 Owner nmrsu
4 Solvent CDCl3
5 Temperature 294.4
6 Pulse Sequence zg30
7 Receiver Gain 75
8 Relaxation Delay 1.0000
9 Pulse Width 8.9200
10 Acquisition Time 3.4079
11 Acquisition Date 2018-10-23T20:35:00
12 Spectrometer Frequency 400.15
13 Spectral Width 9615.4
14 Lowest Frequency -2344.7
15 Nucleus 1H
16 Acquired Size 32768
17 Spectral Size 65536

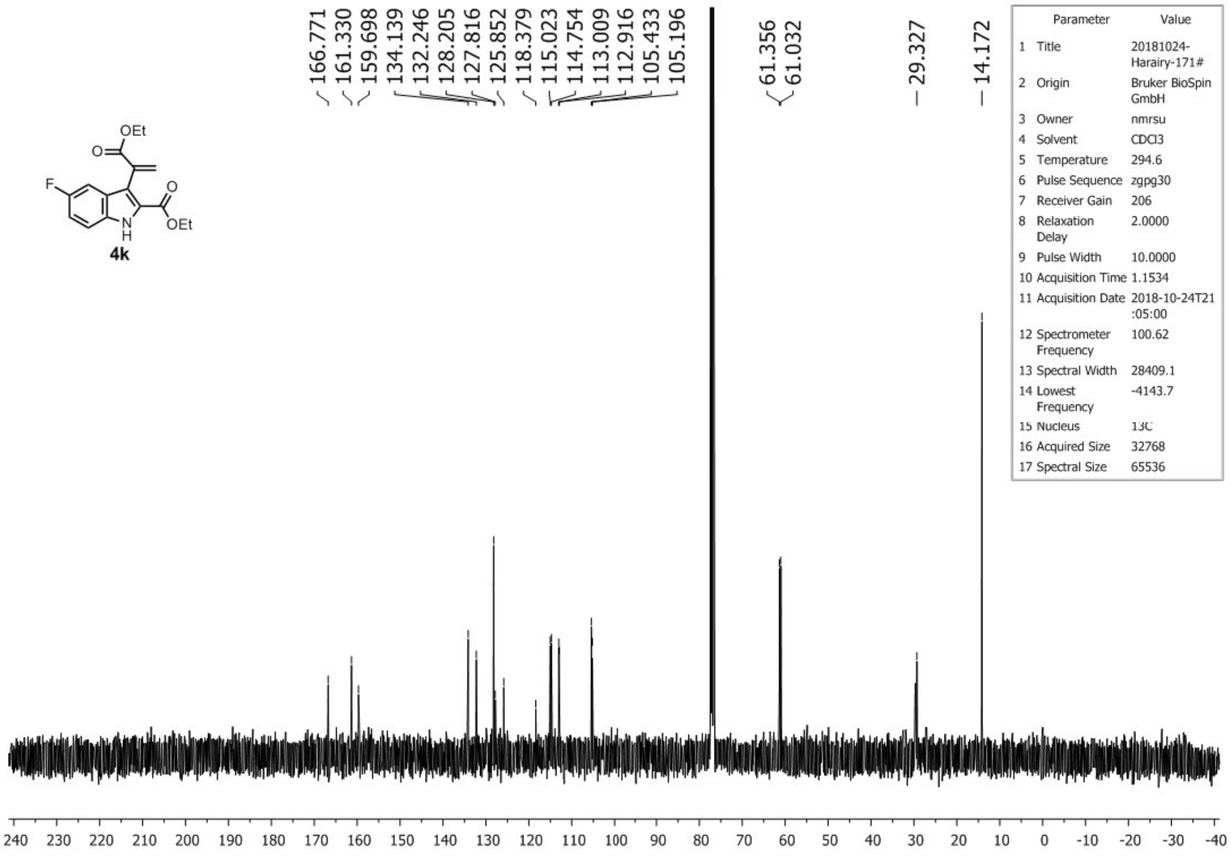

166.771 161.330 159.698 134.139 132.246 128.205 127.816 125.852 118.379 115.023 114.754 113.009 112.916 105.433 105.196
61.356 61.032
29.327
14.172
4k
Parameter / Value
1 Title 20181024-Harairy-171#
2 Origin Bruker BioSpin GmbH
3 Owner nmrsu
4 Solvent CDCl3
5 Temperature 294.6
6 Pulse Sequence zgpg30
7 Receiver Gain 206
8 Relaxation Delay 2.0000
9 Pulse Width 10.0000
10 Acquisition Time 1.1534
11 Acquisition Date 2018-10-24T21:05:00
12 Spectrometer Frequency 100.62
13 Spectral Width 28409.1
14 Lowest Frequency -4143.7
15 Nucleus 13C
16 Acquired Size 32768
17 Spectral Size 65536

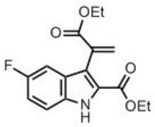

4k

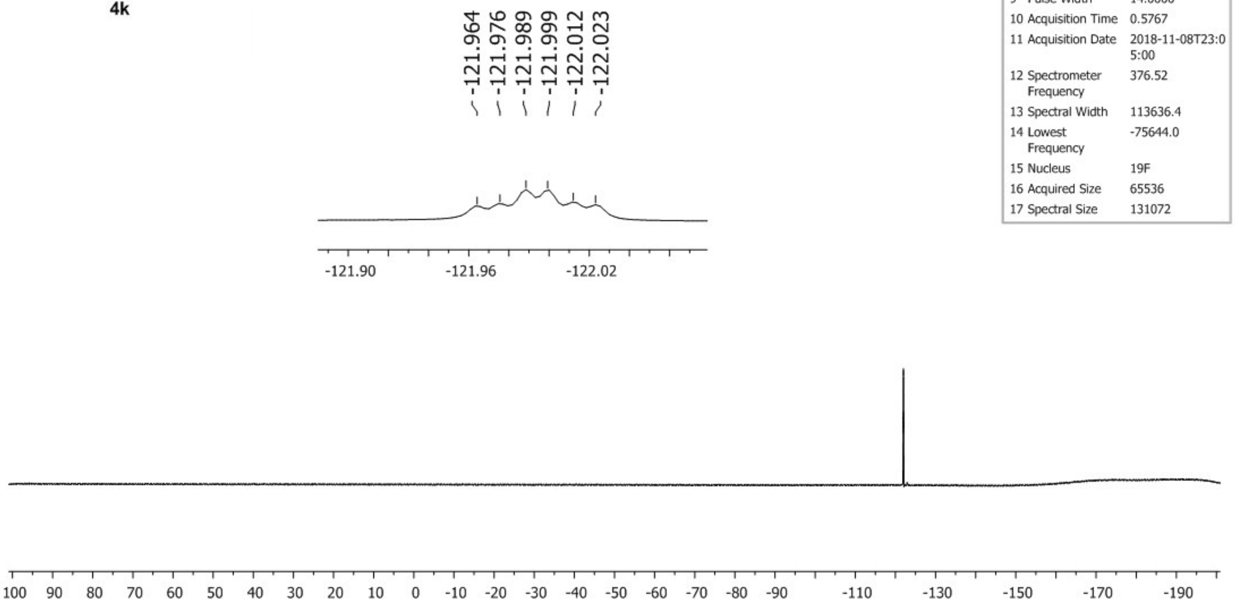

	Parameter	Value
1	Title	20181108-Harairy-171#
2	Origin	Bruker BioSpin GmbH
3	Owner	nmrsu
4	Solvent	CDCl3
5	Temperature	295.3
6	Pulse Sequence	zgflqn
7	Receiver Gain	206
8	Relaxation Delay	1.5000
9	Pulse Width	14.0000
10	Acquisition Time	0.5767
11	Acquisition Date	2018-11-08T23:05:00
12	Spectrometer Frequency	376.52
13	Spectral Width	113636.4
14	Lowest Frequency	-75644.0
15	Nucleus	19F
16	Acquired Size	65536
17	Spectral Size	131072

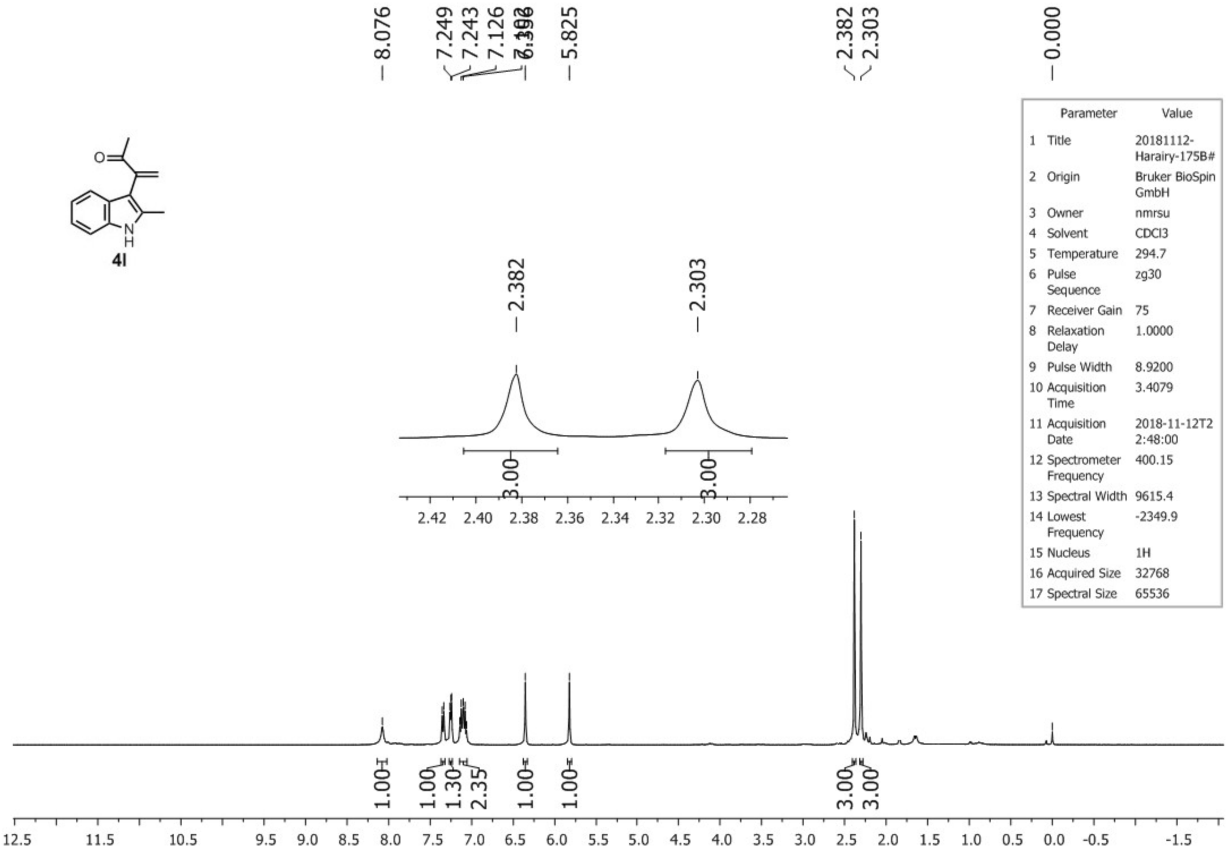
8.076
7.249
7.243
7.126
5.825
2.382
2.303
0.000
4l
1.00
1.00
1.30
2.35
1.00
1.00
3.00
3.00
Parameter Value
1 Title 20181112-Harairy-175B#
2 Origin Bruker BioSpin GmbH
3 Owner nmrsu
4 Solvent CDCl3
5 Temperature 294.7
6 Pulse Sequence zg30
7 Receiver Gain 75
8 Relaxation Delay 1.0000
9 Pulse Width 8.9200
10 Acquisition Time 3.4079
11 Acquisition Date 2018-11-12T2 2:48:00
12 Spectrometer Frequency 400.15
13 Spectral Width 9615.4
14 Lowest Frequency -2349.9
15 Nucleus 1H
16 Acquired Size 32768
17 Spectral Size 65536

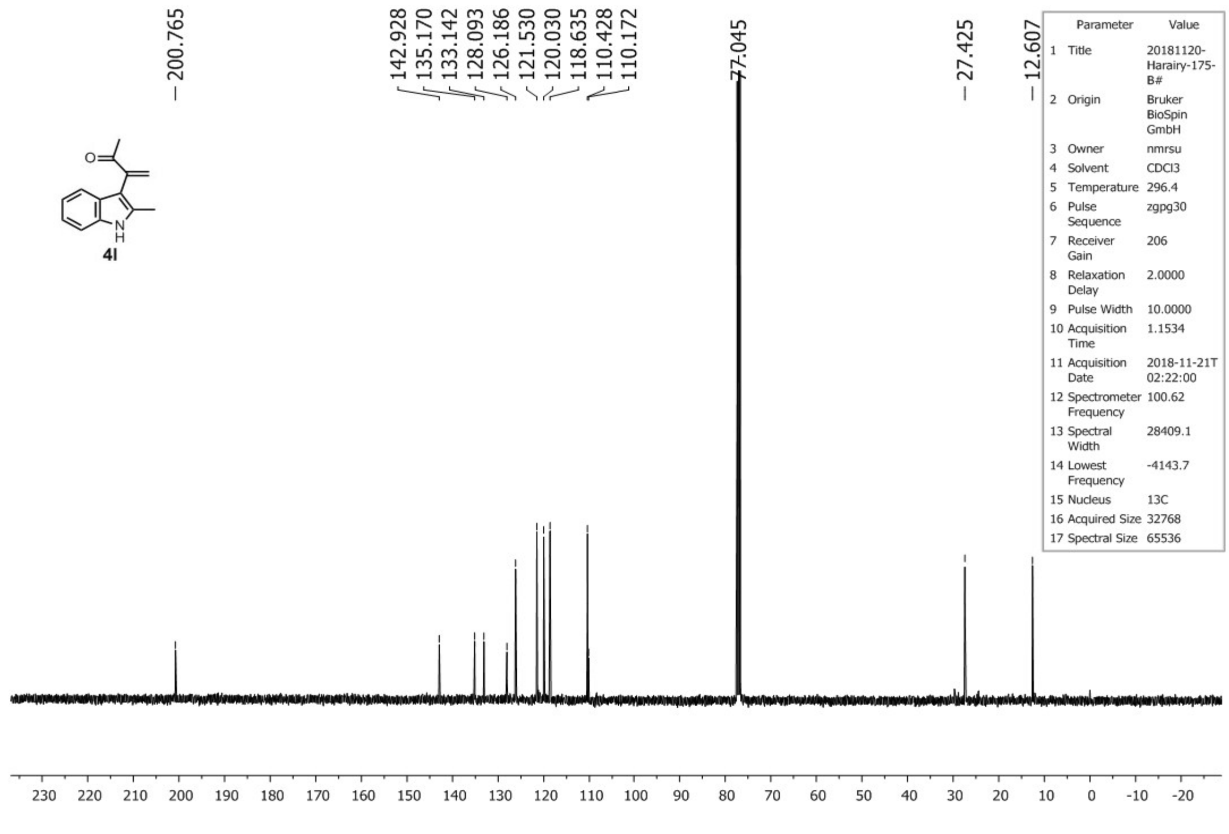
200.765
142.928
135.170
133.142
128.093
126.186
121.530
120.030
118.635
110.428
110.172
77.045
27.425
12.607
4l
Parameter Value
1 Title 20181120-Harairy-175-B#
2 Origin Bruker BioSpin GmbH
3 Owner nmrsu
4 Solvent CDCl3
5 Temperature 296.4
6 Pulse Sequence zgpg30
7 Receiver Gain 206
8 Relaxation Delay 2.0000
9 Pulse Width 10.0000
10 Acquisition Time 1.1534
11 Acquisition Date 2018-11-21T 02:22:00
12 Spectrometer Frequency 100.62
13 Spectral Width 28409.1
14 Lowest Frequency -4143.7
15 Nucleus 13C
16 Acquired Size 32768
17 Spectral Size 65536

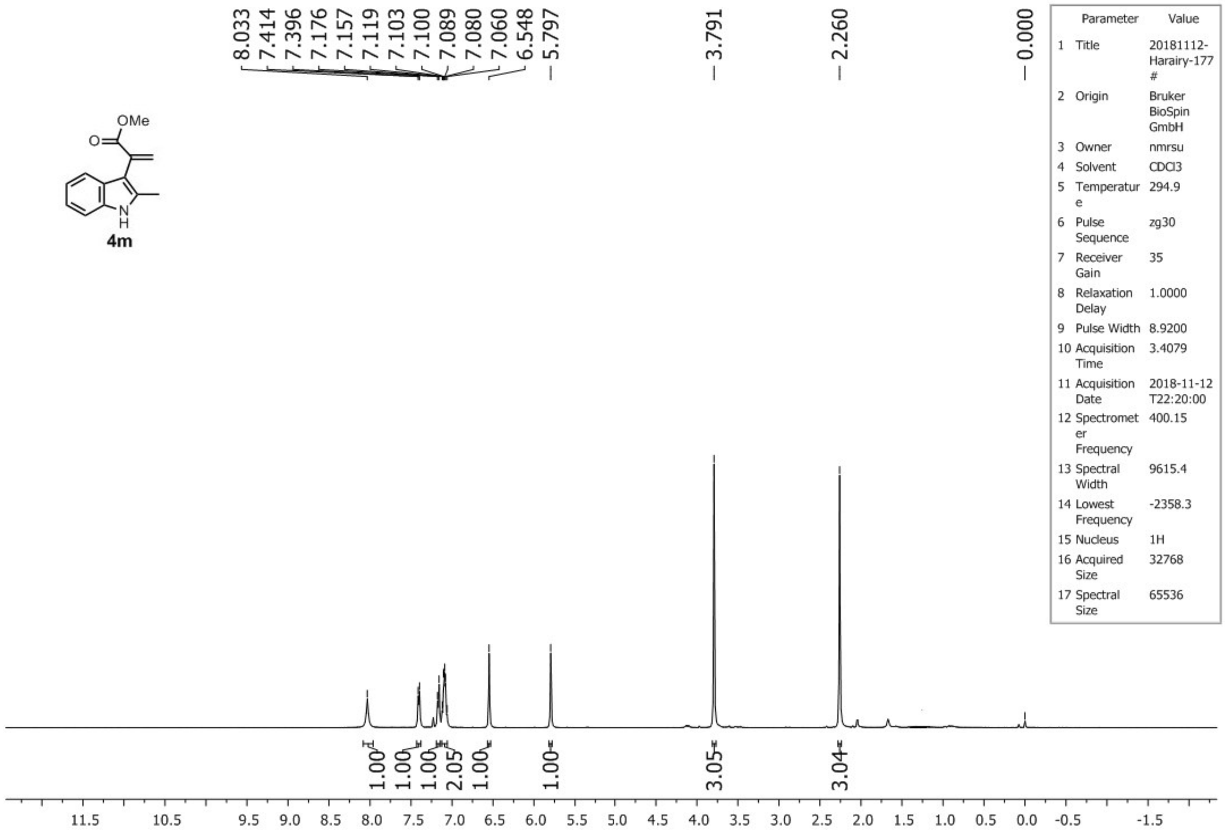
8.033
7.414
7.396
7.176
7.157
7.119
7.103
7.100
7.089
7.080
7.060
6.548
5.797
3.791
2.260
0.000
OMe
O
N
H
4m
Parameter Value
1 Title 20181112-Harairy-177#
2 Origin Bruker BioSpin GmbH
3 Owner nmrsu
4 Solvent CDCl3
5 Temperature 294.9
6 Pulse Sequence zg30
7 Receiver Gain 35
8 Relaxation Delay 1.0000
9 Pulse Width 8.9200
10 Acquisition Time 3.4079
11 Acquisition Date 2018-11-12T22:20:00
12 Spectrometer Frequency 400.15
13 Spectral Width 9615.4
14 Lowest Frequency -2358.3
15 Nucleus 1H
16 Acquired Size 32768
17 Spectral Size 65536
1.00
1.00
1.00
2.05
1.00
1.00
3.05
3.04
11.5 10.5 9.5 9.0 8.5 8.0 7.5 7.0 6.5 6.0 5.5 5.0 4.5 4.0 3.5 3.0 2.5 2.0 1.5 1.0 0.5 0.0 -0.5 -1.5

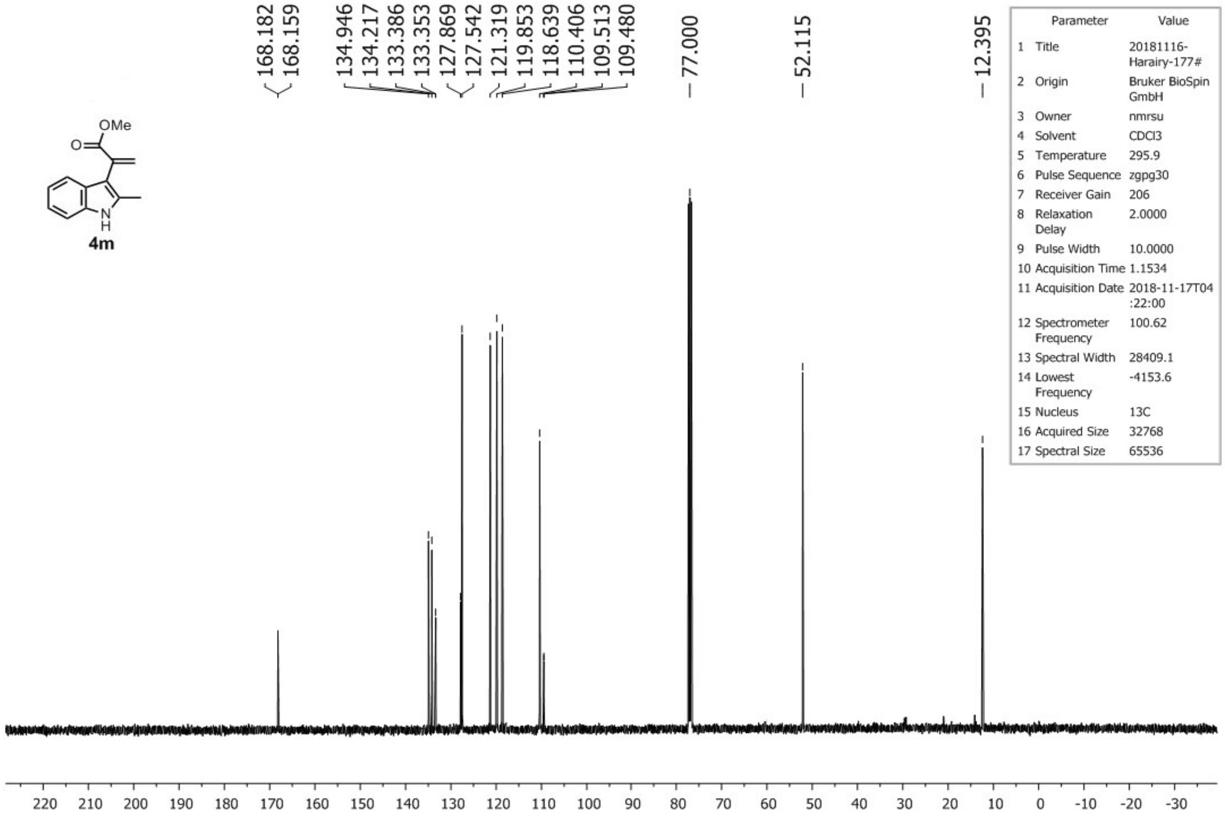
168.182
168.159
134.946
134.217
133.386
133.353
127.869
127.542
121.319
119.853
118.639
110.406
109.513
109.480
77.000
52.115
12.395
OMe
O
N
H
4m
Parameter Value
1 Title 20181116-Harairy-177#
2 Origin Bruker BioSpin GmbH
3 Owner nmrsu
4 Solvent CDCl3
5 Temperature 295.9
6 Pulse Sequence zgpg30
7 Receiver Gain 206
8 Relaxation Delay 2.0000
9 Pulse Width 10.0000
10 Acquisition Time 1.1534
11 Acquisition Date 2018-11-17T04:22:00
12 Spectrometer Frequency 100.62
13 Spectral Width 28409.1
14 Lowest Frequency -4153.6
15 Nucleus 13C
16 Acquired Size 32768
17 Spectral Size 65536
220 210 200 190 180 170 160 150 140 130 120 110 100 90 80 70 60 50 40 30 20 10 0 -10 -20 -30

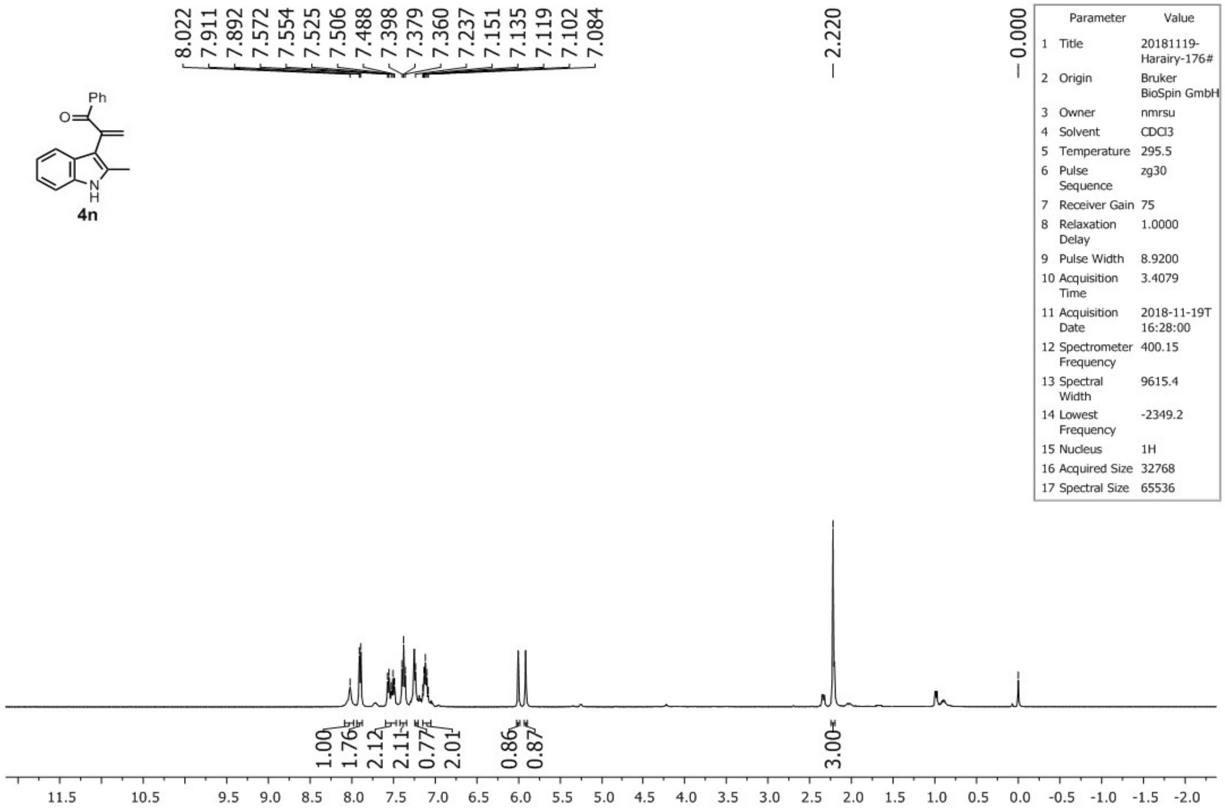

	Parameter	Value
1	Title	20181119-Harairy-176#
2	Origin	Bruker BioSpin GmbH
3	Owner	nmrsu
4	Solvent	CDCl3
5	Temperature	295.5
6	Pulse Sequence	zg30
7	Receiver Gain	75
8	Relaxation Delay	1.0000
9	Pulse Width	8.9200
10	Acquisition Time	3.4079
11	Acquisition Date	2018-11-19T 16:28:00
12	Spectrometer Frequency	400.15
13	Spectral Width	9615.4
14	Lowest Frequency	-2349.2
15	Nucleus	1H
16	Acquired Size	32768
17	Spectral Size	65536

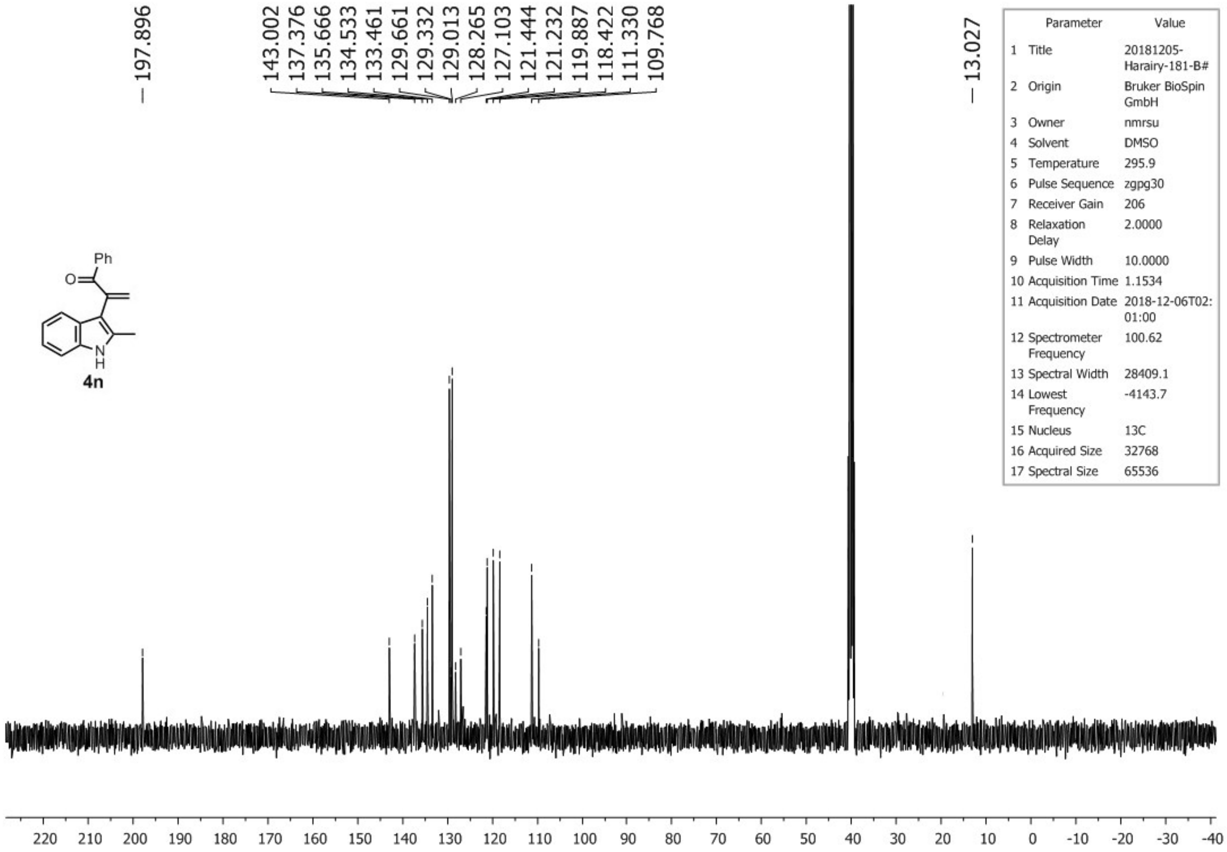

	Parameter	Value
1	Title	20181205-Harairy-181-B#
2	Origin	Bruker BioSpin GmbH
3	Owner	nmrsu
4	Solvent	DMSO
5	Temperature	295.9
6	Pulse Sequence	zgpg30
7	Receiver Gain	206
8	Relaxation Delay	2.0000
9	Pulse Width	10.0000
10	Acquisition Time	1.1534
11	Acquisition Date	2018-12-06T02:01:00
12	Spectrometer Frequency	100.62
13	Spectral Width	28409.1
14	Lowest Frequency	-4143.7
15	Nucleus	13C
16	Acquired Size	32768
17	Spectral Size	65536

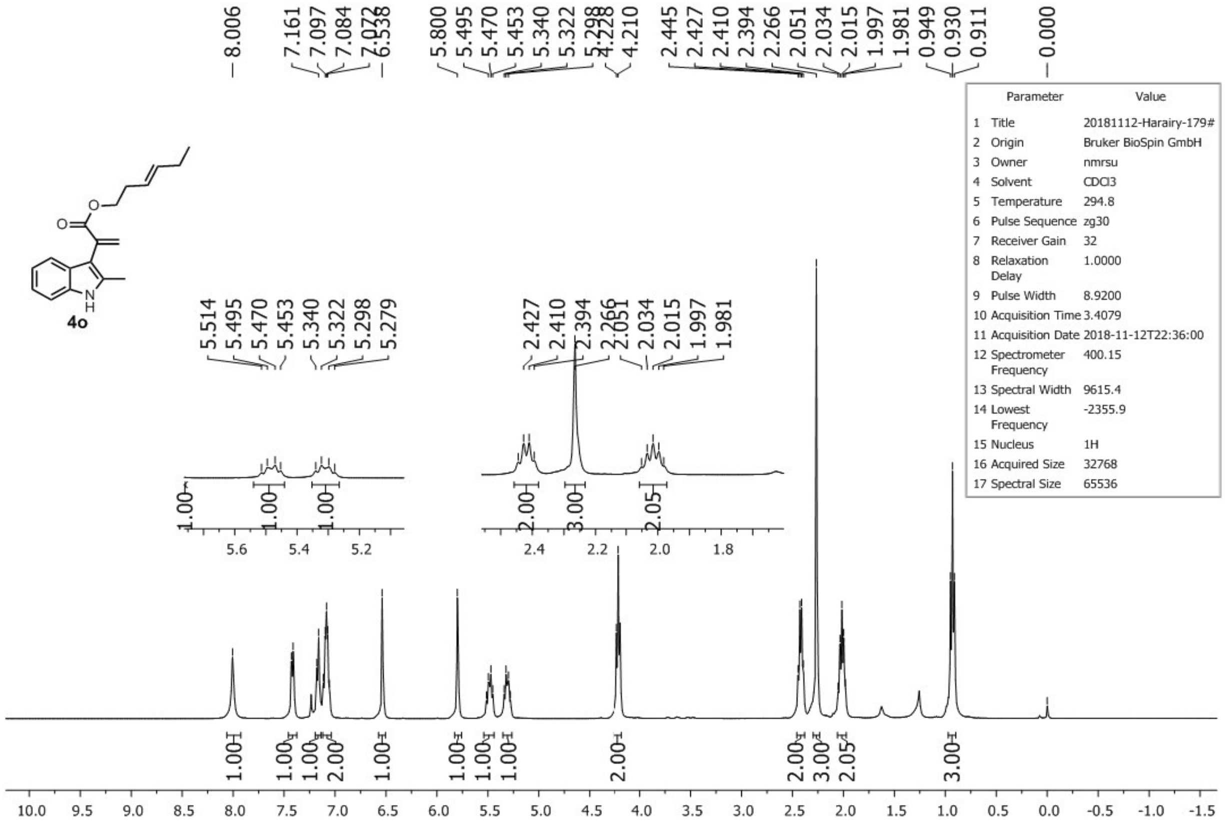
8.006
7.161
7.097
7.084
7.072
6.538
5.800
5.495
5.470
5.453
5.340
5.322
5.298
4.228
4.210
2.445
2.427
2.410
2.394
2.266
2.051
2.034
2.015
1.997
1.981
0.949
0.930
0.911
0.000
4o
Parameter Value
1 Title 20181112-Harairy-179#
2 Origin Bruker BioSpin GmbH
3 Owner nmrsu
4 Solvent CDCl3
5 Temperature 294.8
6 Pulse Sequence zg30
7 Receiver Gain 32
8 Relaxation Delay 1.0000
9 Pulse Width 8.9200
10 Acquisition Time 3.4079
11 Acquisition Date 2018-11-12T22:36:00
12 Spectrometer Frequency 400.15
13 Spectral Width 9615.4
14 Lowest Frequency -2355.9
15 Nucleus 1H
16 Acquired Size 32768
17 Spectral Size 65536
5.514
5.495
5.470
5.453
5.340
5.322
5.298
5.279
2.427
2.410
2.394
2.266
2.051
2.034
2.015
1.997
1.981
1.00
1.00
1.00
2.00
3.00
2.05
5.6 5.4 5.2
2.4 2.2 2.0 1.8
1.00 1.00 1.00 2.00 1.00 1.00 1.00 1.00 2.00 2.00 3.00 2.05 3.00
10.0 9.5 9.0 8.5 8.0 7.5 7.0 6.5 6.0 5.5 5.0 4.5 4.0 3.5 3.0 2.5 2.0 1.5 1.0 0.5 0.0 -0.5 -1.0 -1.5

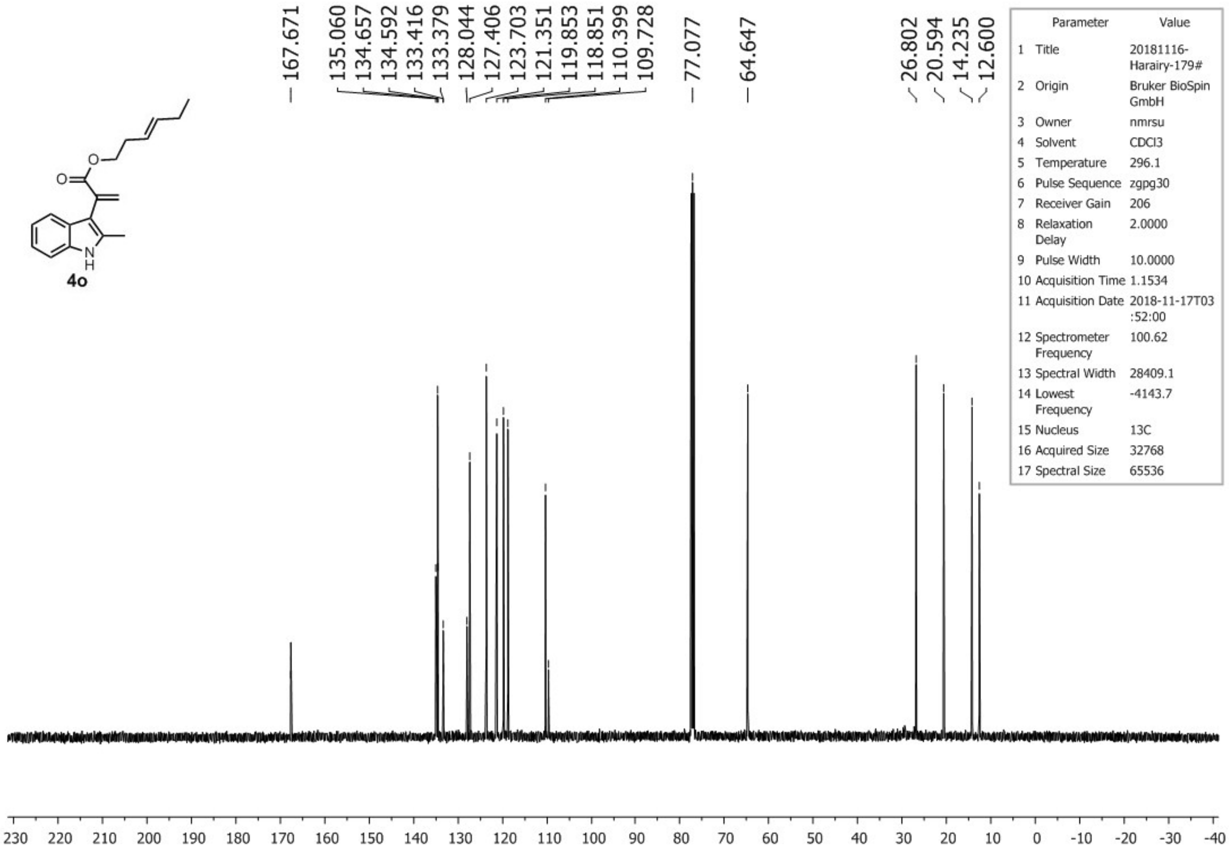
167.671
135.060
134.657
134.592
133.416
133.379
128.044
127.406
123.703
121.351
119.853
118.851
110.399
109.728
77.077
64.647
26.802
20.594
14.235
12.600
4o
Parameter Value
1 Title 20181116-Harairy-179#
2 Origin Bruker BioSpin GmbH
3 Owner nmrsu
4 Solvent CDCl3
5 Temperature 296.1
6 Pulse Sequence zgpg30
7 Receiver Gain 206
8 Relaxation Delay 2.0000
9 Pulse Width 10.0000
10 Acquisition Time 1.1534
11 Acquisition Date 2018-11-17T03:52:00
12 Spectrometer Frequency 100.62
13 Spectral Width 28409.1
14 Lowest Frequency -4143.7
15 Nucleus 13C
16 Acquired Size 32768
17 Spectral Size 65536
230 220 210 200 190 180 170 160 150 140 130 120 110 100 90 80 70 60 50 40 30 20 10 0 -10 -20 -30 -40

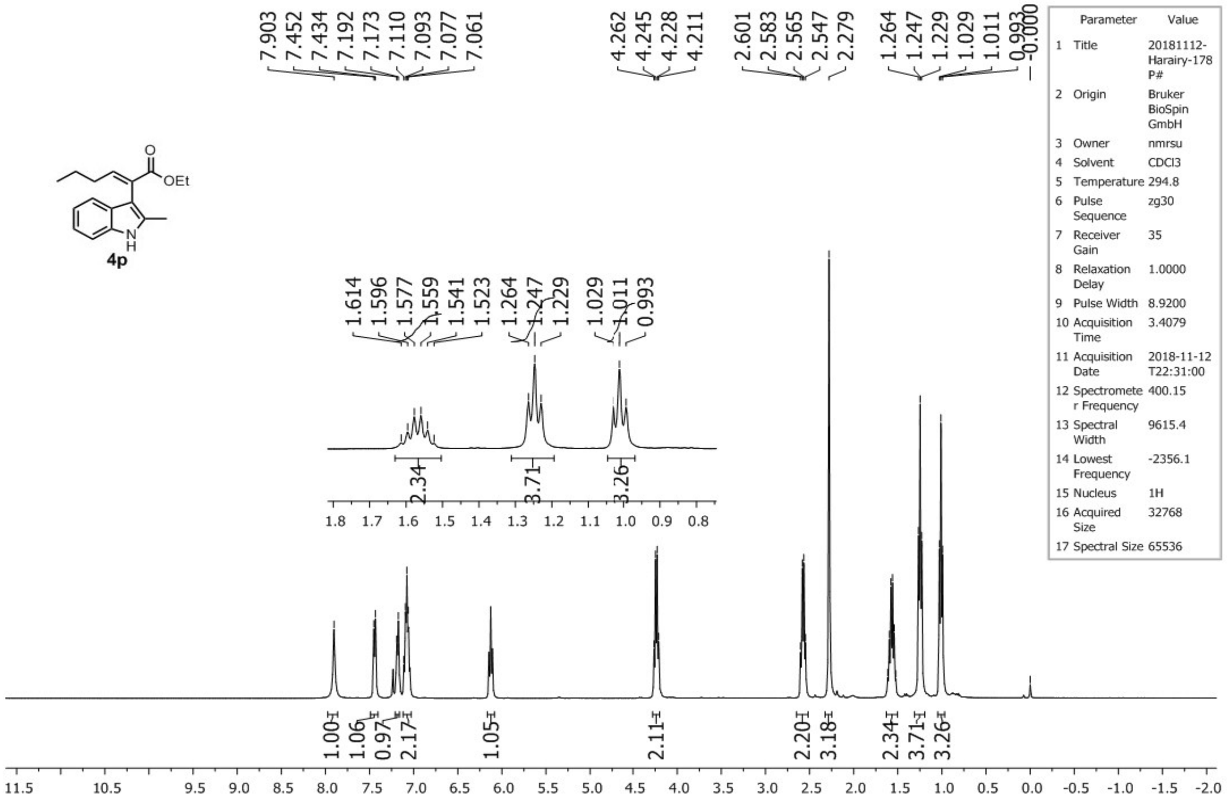
7.903
7.452
7.434
7.192
7.173
7.110
7.093
7.077
7.061
4.262
4.245
4.228
4.211
2.601
2.583
2.565
2.547
2.279
1.264
1.247
1.229
1.029
1.011
0.993
0.000
OEt
O
N
H
4p
1.614
1.596
1.577
1.559
1.541
1.523
1.264
1.247
1.229
1.029
1.011
0.993
2.34
3.71
3.26
1.8 1.7 1.6 1.5 1.4 1.3 1.2 1.1 1.0 0.9 0.8
1.00
1.06
0.97
2.17
1.05
2.11
2.20
3.18
2.34
3.71
3.26
11.5 10.5 9.5 9.0 8.5 8.0 7.5 7.0 6.5 6.0 5.5 5.0 4.5 4.0 3.5 3.0 2.5 2.0 1.5 1.0 0.5 0.0 -0.5 -1.0 -1.5 -2.0
Parameter Value
1 Title 20181112-Harairy-178 P#
2 Origin Bruker BioSpin GmbH
3 Owner nmrsu
4 Solvent CDCl3
5 Temperature 294.8
6 Pulse Sequence zg30
7 Receiver Gain 35
8 Relaxation Delay 1.0000
9 Pulse Width 8.9200
10 Acquisition Time 3.4079
11 Acquisition Date 2018-11-12 T22:31:00
12 Spectrometer Frequency 400.15
13 Spectral Width 9615.4
14 Lowest Frequency -2356.1
15 Nucleus 1H
16 Acquired Size 32768
17 Spectral Size 65536

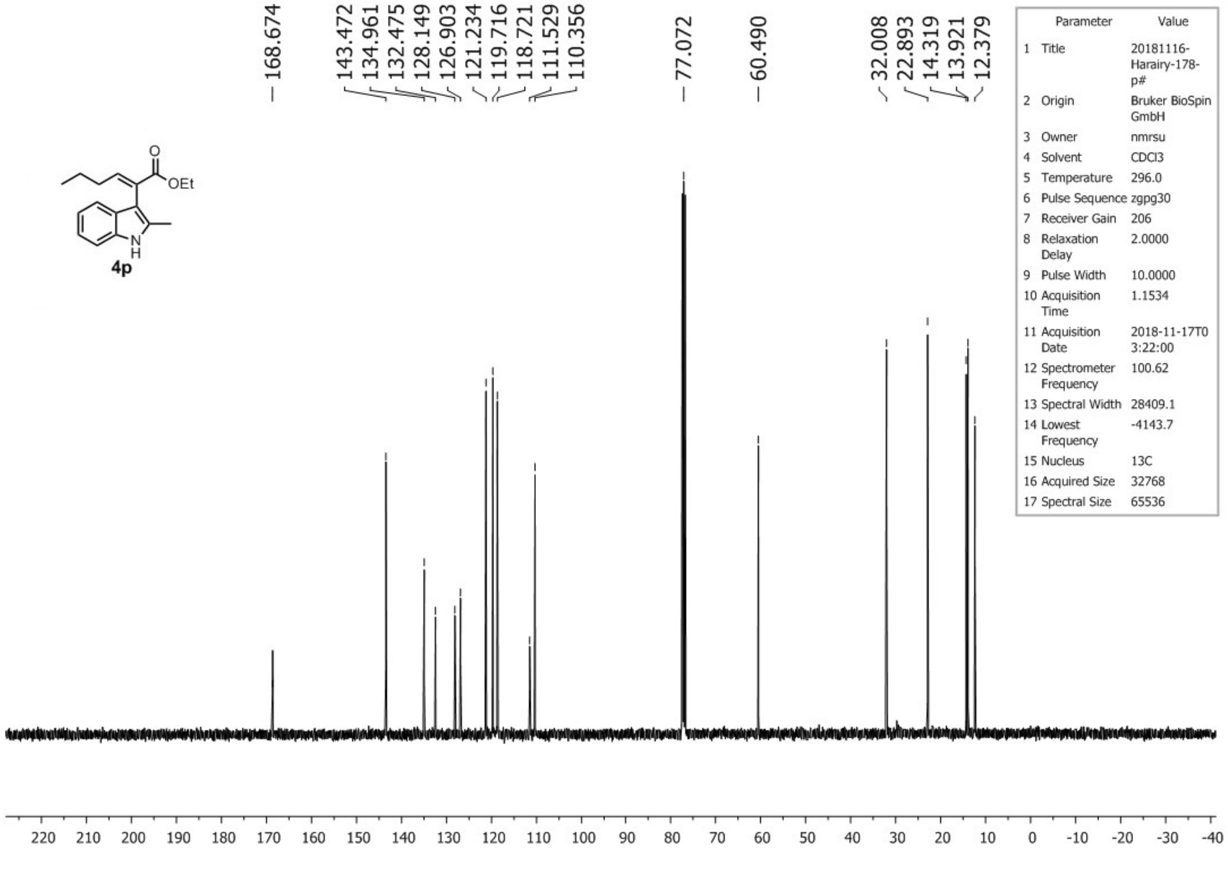
168.674
143.472
134.961
132.475
128.149
126.903
121.234
119.716
118.721
111.529
110.356
77.072
60.490
32.008
22.893
14.319
13.921
12.379
OEt
O
N
H
4p
220 210 200 190 180 170 160 150 140 130 120 110 100 90 80 70 60 50 40 30 20 10 0 -10 -20 -30 -40
Parameter Value
1 Title 20181116-Harairy-178-p#
2 Origin Bruker BioSpin GmbH
3 Owner nmrsu
4 Solvent CDCl3
5 Temperature 296.0
6 Pulse Sequence zgpg30
7 Receiver Gain 206
8 Relaxation Delay 2.0000
9 Pulse Width 10.0000
10 Acquisition Time 1.1534
11 Acquisition Date 2018-11-17T03:22:00
12 Spectrometer Frequency 100.62
13 Spectral Width 28409.1
14 Lowest Frequency -4143.7
15 Nucleus 13C
16 Acquired Size 32768
17 Spectral Size 65536

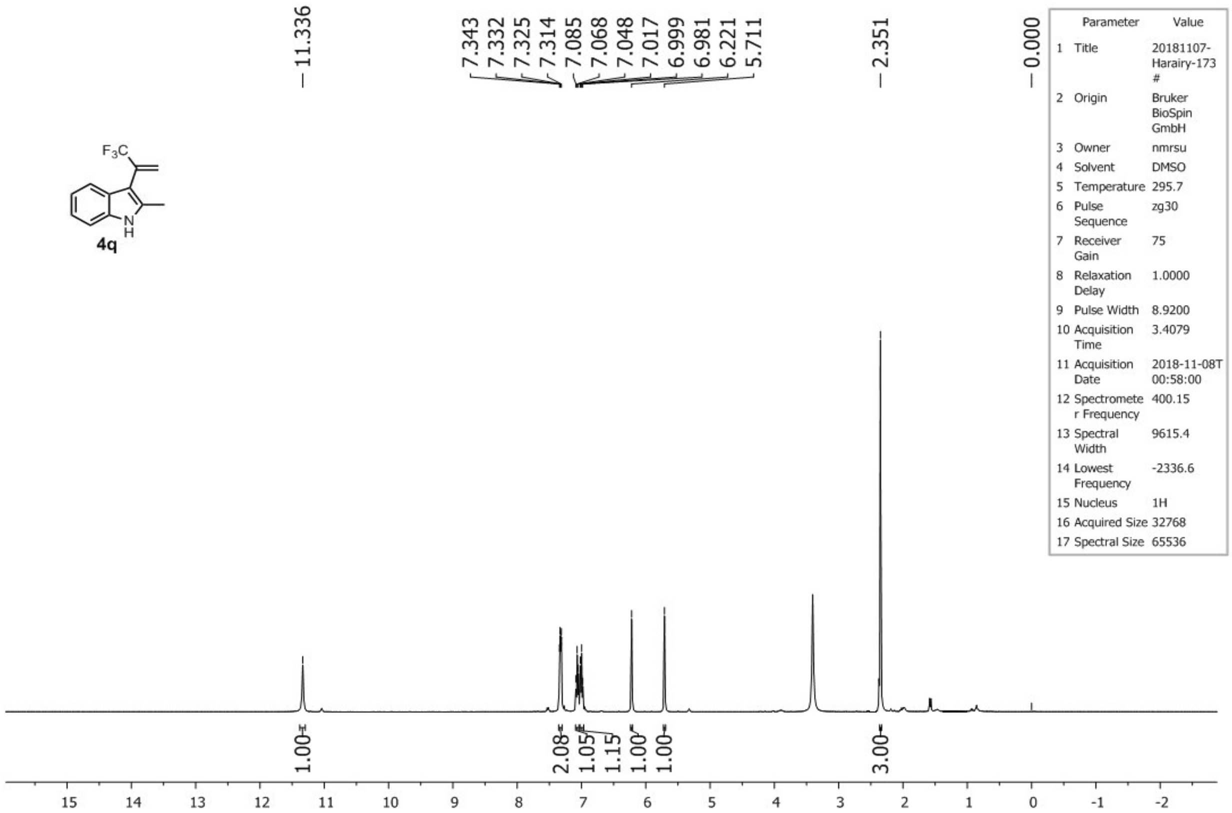

	Parameter	Value
1	Title	20181107-Harairy-173 #
2	Origin	Bruker BioSpin GmbH
3	Owner	nmrsu
4	Solvent	DMSO
5	Temperature	295.7
6	Pulse Sequence	zg30
7	Receiver Gain	75
8	Relaxation Delay	1.0000
9	Pulse Width	8.9200
10	Acquisition Time	3.4079
11	Acquisition Date	2018-11-08T 00:58:00
12	Spectrometer Frequency	400.15
13	Spectral Width	9615.4
14	Lowest Frequency	-2336.6
15	Nucleus	1H
16	Acquired Size	32768
17	Spectral Size	65536

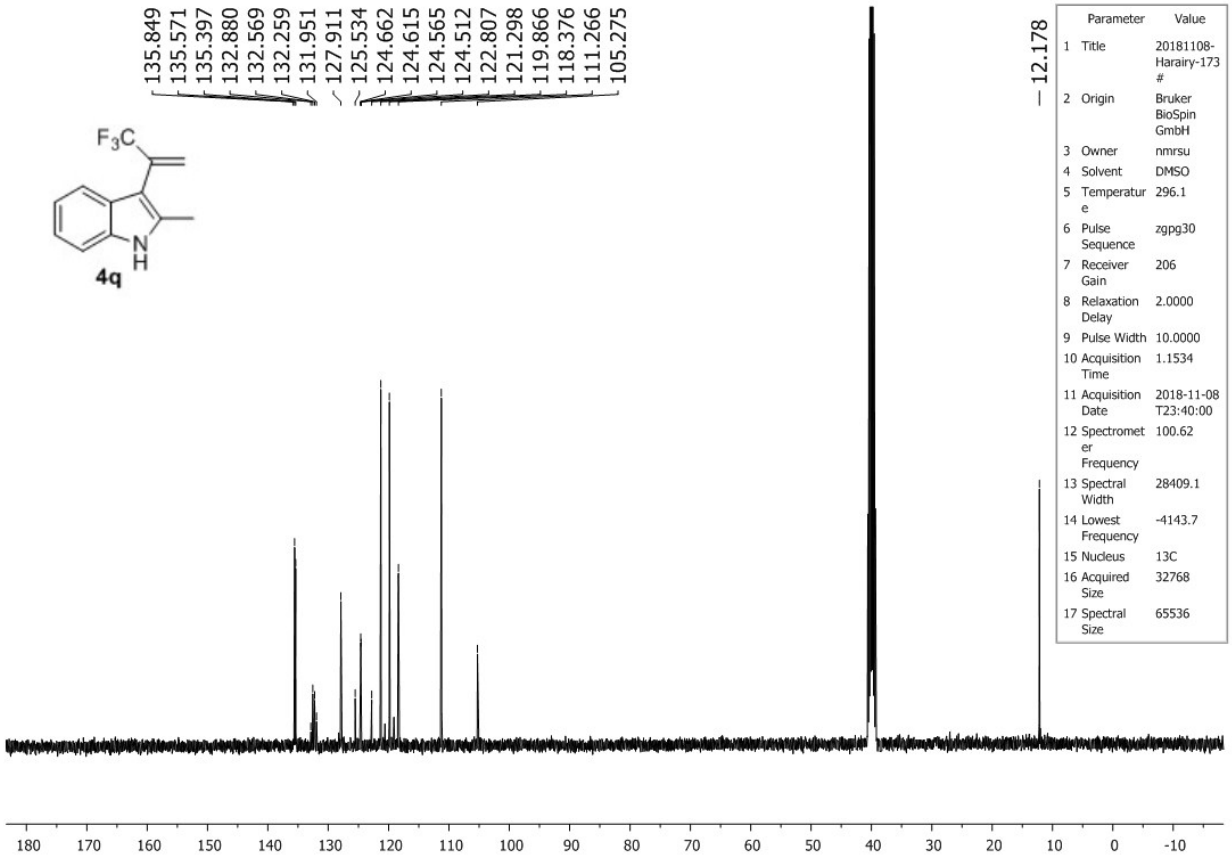

	Parameter	Value
1	Title	20181108-Harairy-173 #
2	Origin	Bruker BioSpin GmbH
3	Owner	nmrsu
4	Solvent	DMSO
5	Temperature	296.1
6	Pulse Sequence	zgpg30
7	Receiver Gain	206
8	Relaxation Delay	2.0000
9	Pulse Width	10.0000
10	Acquisition Time	1.1534
11	Acquisition Date	2018-11-08 T23:40:00
12	Spectrometer Frequency	100.62
13	Spectral Width	28409.1
14	Lowest Frequency	-4143.7
15	Nucleus	13C
16	Acquired Size	32768
17	Spectral Size	65536

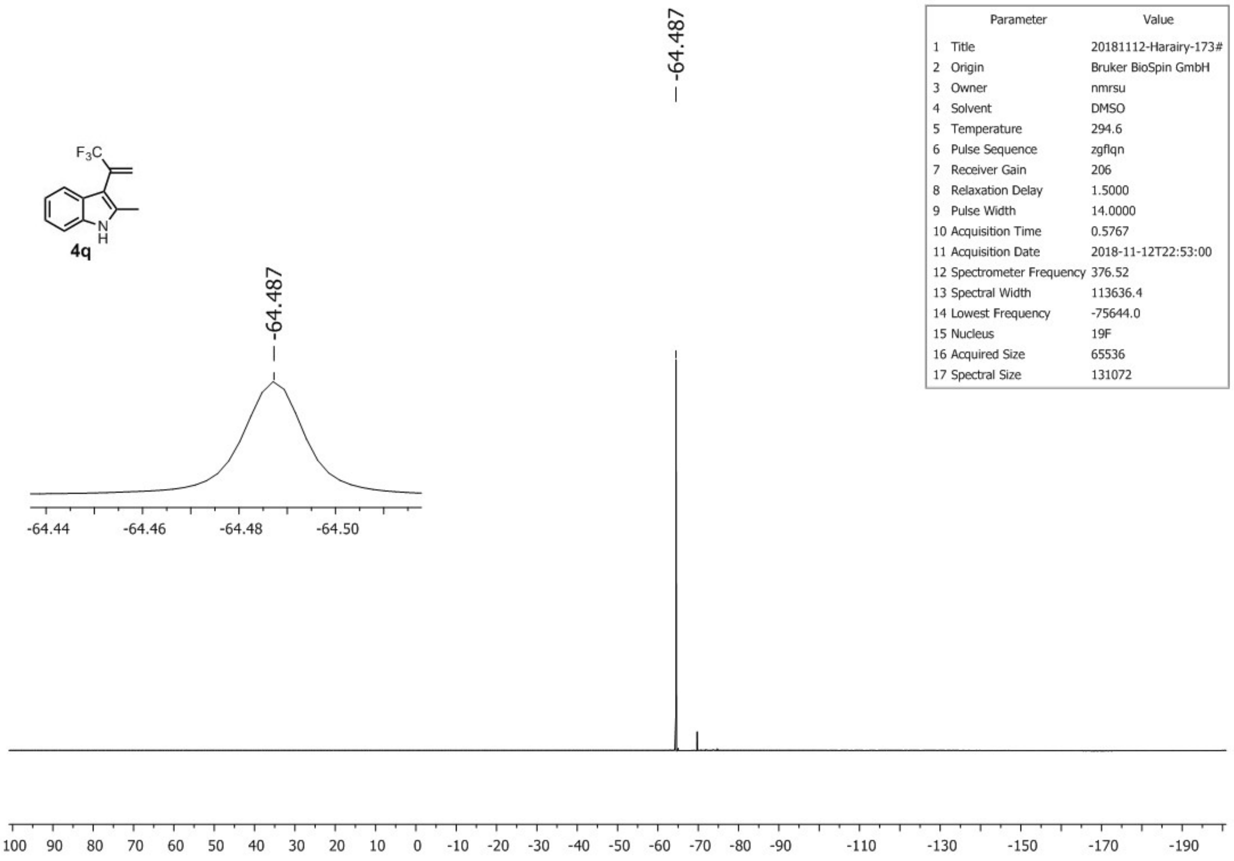
-64.487
F3C
N
H
4q
-64.487
-64.44 -64.46 -64.48 -64.50
Parameter Value
1 Title 20181112-Harairy-173#
2 Origin Bruker BioSpin GmbH
3 Owner nmrsu
4 Solvent DMSO
5 Temperature 294.6
6 Pulse Sequence zgflqn
7 Receiver Gain 206
8 Relaxation Delay 1.5000
9 Pulse Width 14.0000
10 Acquisition Time 0.5767
11 Acquisition Date 2018-11-12T22:53:00
12 Spectrometer Frequency 376.52
13 Spectral Width 113636.4
14 Lowest Frequency -75644.0
15 Nucleus 19F
16 Acquired Size 65536
17 Spectral Size 131072
100 90 80 70 60 50 40 30 20 10 0 -10 -20 -30 -40 -50 -60 -70 -80 -90 -110 -130 -150 -170 -190

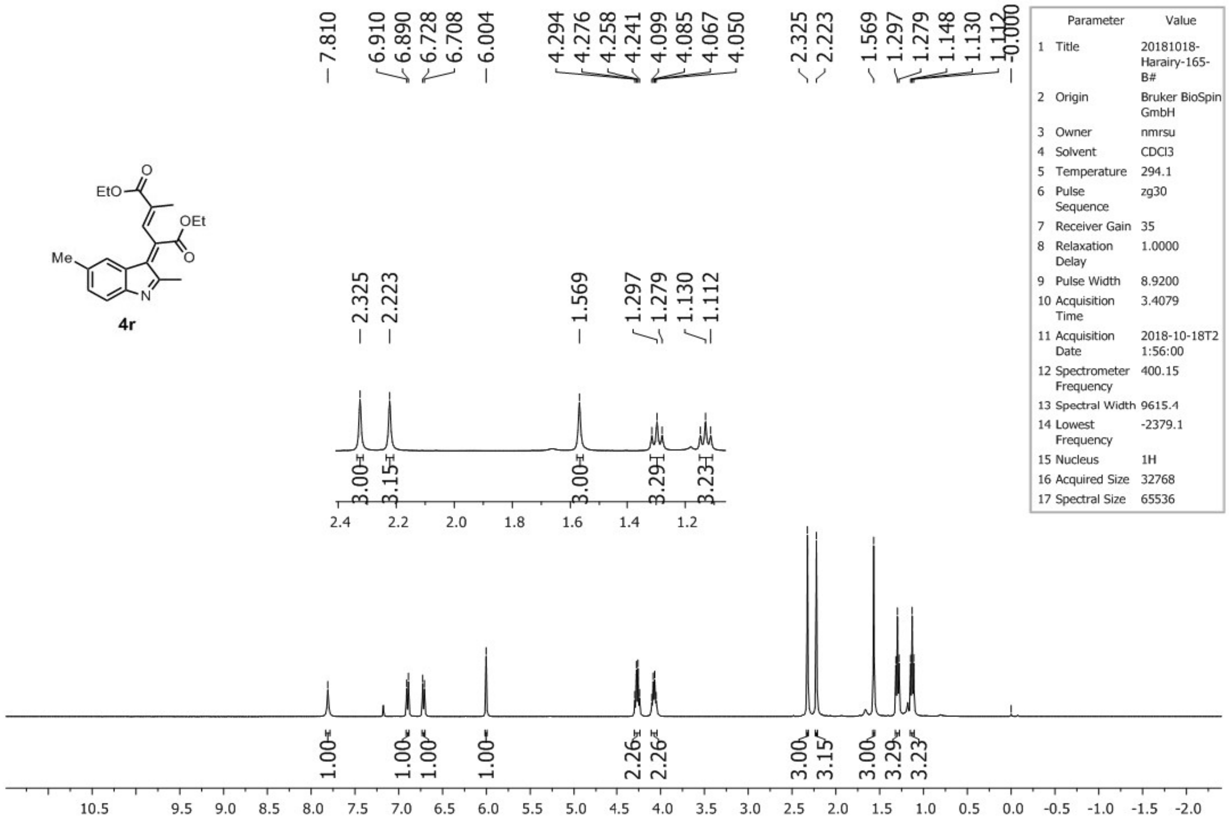

	Parameter	Value
1	Title	20181018-Harairy-165-B#
2	Origin	Bruker BioSpin GmbH
3	Owner	nmrsu
4	Solvent	CDCl3
5	Temperature	294.1
6	Pulse Sequence	zg30
7	Receiver Gain	35
8	Relaxation Delay	1.0000
9	Pulse Width	8.9200
10	Acquisition Time	3.4079
11	Acquisition Date	2018-10-18T21:56:00
12	Spectrometer Frequency	400.15
13	Spectral Width	9615.4
14	Lowest Frequency	-2379.1
15	Nucleus	1H
16	Acquired Size	32768
17	Spectral Size	65536

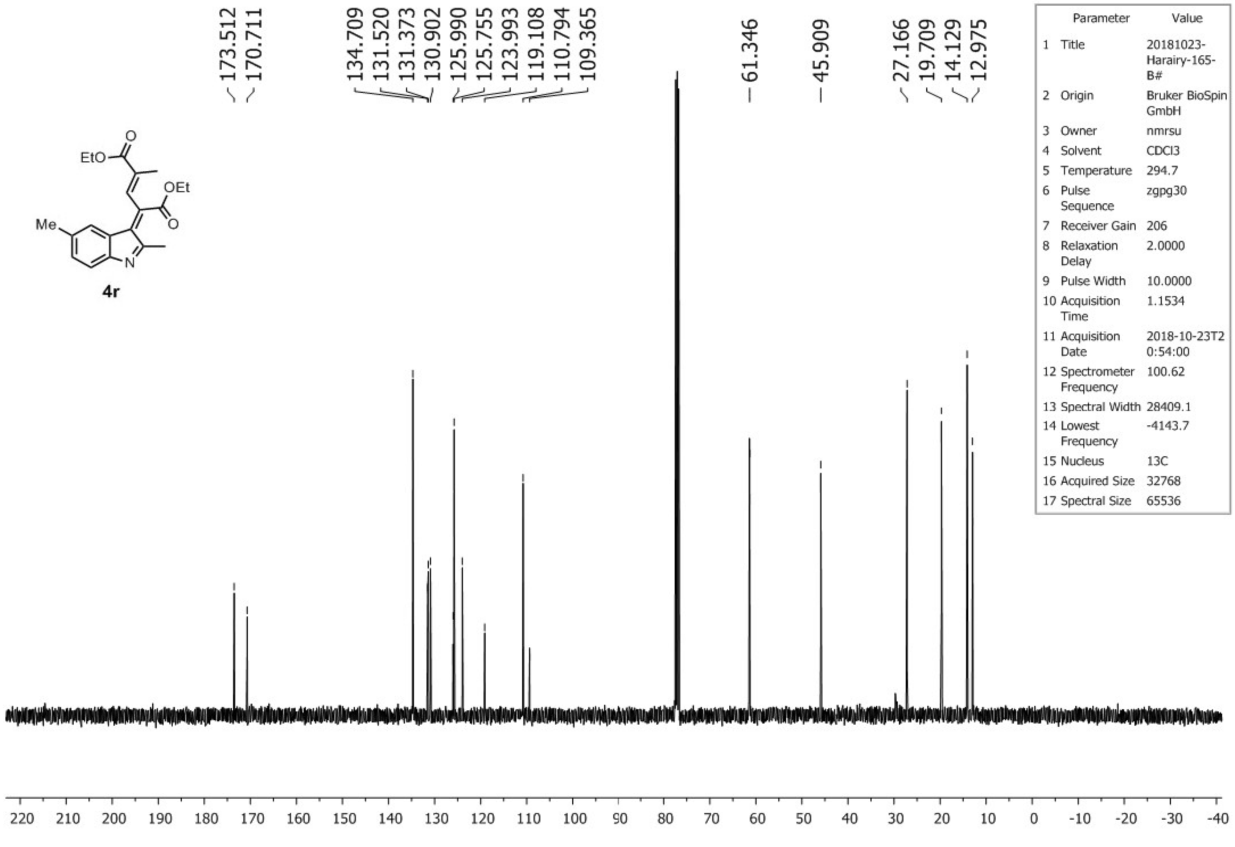

	Parameter	Value
1	Title	20181023-Harairy-165-B#
2	Origin	Bruker BioSpin GmbH
3	Owner	nmrsu
4	Solvent	CDCl3
5	Temperature	294.7
6	Pulse Sequence	zgpg30
7	Receiver Gain	206
8	Relaxation Delay	2.0000
9	Pulse Width	10.0000
10	Acquisition Time	1.1534
11	Acquisition Date	2018-10-23T20:54:00
12	Spectrometer Frequency	100.62
13	Spectral Width	28409.1
14	Lowest Frequency	-4143.7
15	Nucleus	13C
16	Acquired Size	32768
17	Spectral Size	65536

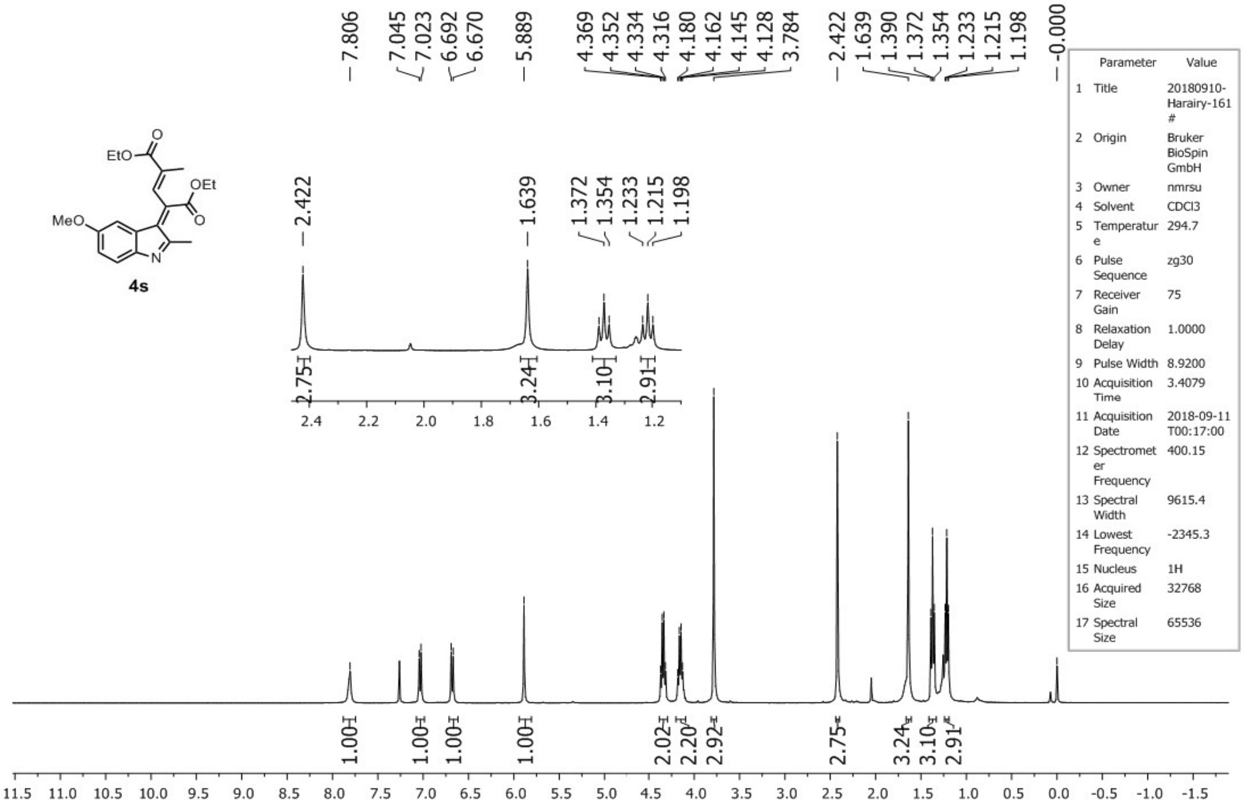

	Parameter	Value
1	Title	20180910-Harairy-161#
2	Origin	Bruker BioSpin GmbH
3	Owner	nmrsu
4	Solvent	CDCl3
5	Temperature	294.7
6	Pulse Sequence	zg30
7	Receiver Gain	75
8	Relaxation Delay	1.0000
9	Pulse Width	8.9200
10	Acquisition Time	3.4079
11	Acquisition Date	2018-09-11T00:17:00
12	Spectrometer Frequency	400.15
13	Spectral Width	9615.4
14	Lowest Frequency	-2345.3
15	Nucleus	1H
16	Acquired Size	32768
17	Spectral Size	65536

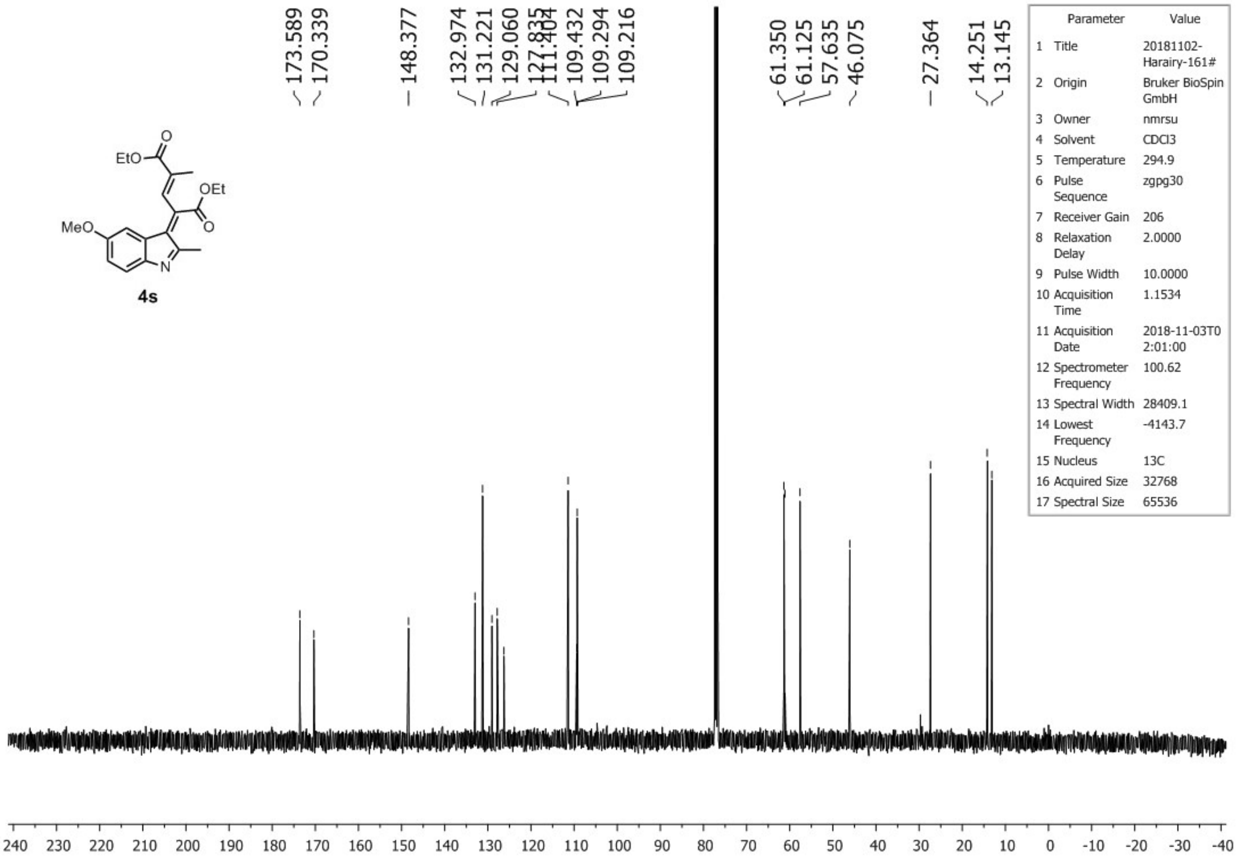

	Parameter	Value
1	Title	20181102-Harairy-161#
2	Origin	Bruker BioSpin GmbH
3	Owner	nmrsu
4	Solvent	CDCl3
5	Temperature	294.9
6	Pulse Sequence	zgpg30
7	Receiver Gain	206
8	Relaxation Delay	2.0000
9	Pulse Width	10.0000
10	Acquisition Time	1.1534
11	Acquisition Date	2018-11-03T02:01:00
12	Spectrometer Frequency	100.62
13	Spectral Width	28409.1
14	Lowest Frequency	-4143.7
15	Nucleus	13C
16	Acquired Size	32768
17	Spectral Size	65536

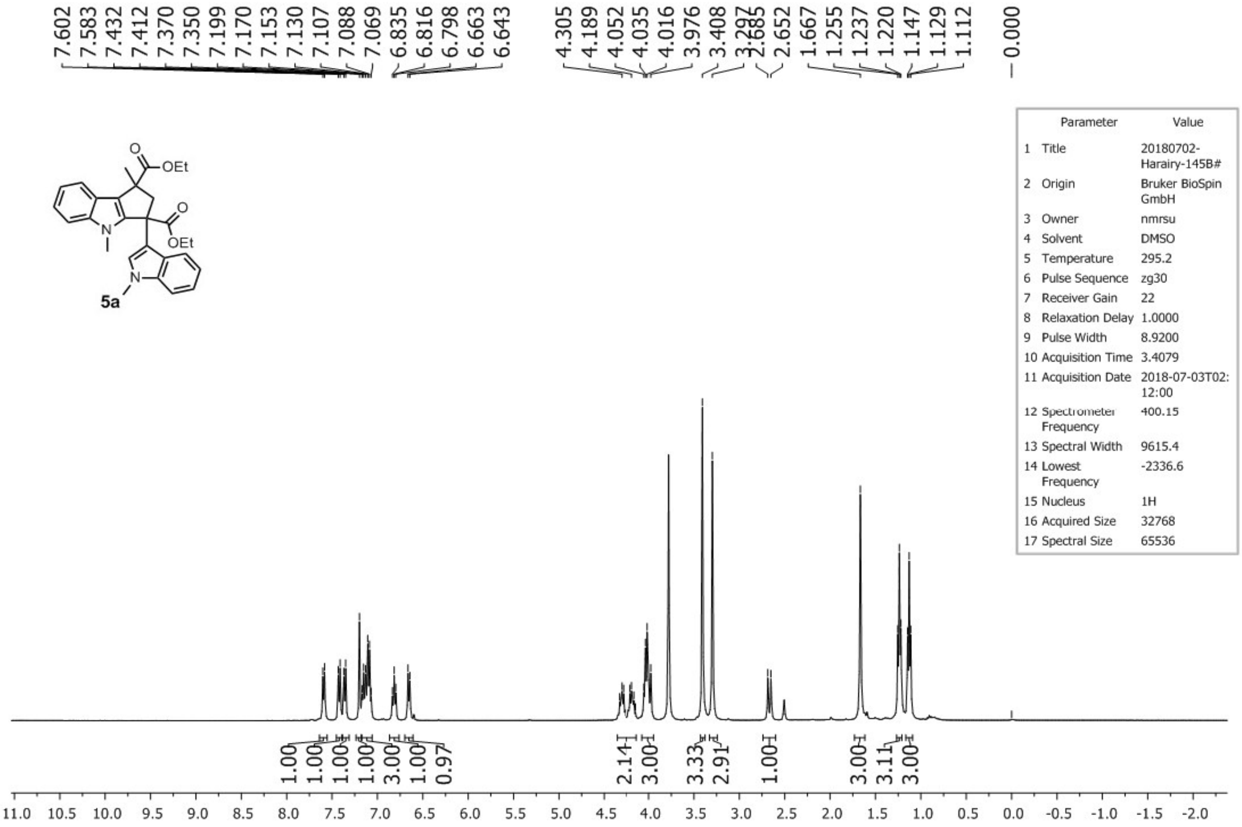
7.602 7.583 7.432 7.412 7.370 7.350 7.199 7.170 7.153 7.130 7.107 7.088 7.069 6.835 6.816 6.798 6.663 6.643
4.305 4.189 4.052 4.035 4.016 3.976 3.408 3.297 2.685 2.652 1.667 1.255 1.237 1.220 1.147 1.129 1.112
0.000
OEt
O
OEt
O
N
N
5a
Parameter Value
1 Title 20180702-Harairy-145B#
2 Origin Bruker BioSpin GmbH
3 Owner nmrsu
4 Solvent DMSO
5 Temperature 295.2
6 Pulse Sequence zg30
7 Receiver Gain 22
8 Relaxation Delay 1.0000
9 Pulse Width 8.9200
10 Acquisition Time 3.4079
11 Acquisition Date 2018-07-03T02:12:00
12 Spectrometer Frequency 400.15
13 Spectral Width 9615.4
14 Lowest Frequency -2336.6
15 Nucleus 1H
16 Acquired Size 32768
17 Spectral Size 65536
1.00 1.00 1.00 1.00 3.00 1.00 0.97 2.14 3.00 3.33 2.91 1.00 3.00 3.11 3.00
11.0 10.5 10.0 9.5 9.0 8.5 8.0 7.5 7.0 6.5 6.0 5.5 5.0 4.5 4.0 3.5 3.0 2.5 2.0 1.5 1.0 0.5 0.0 -0.5 -1.0 -1.5 -2.0

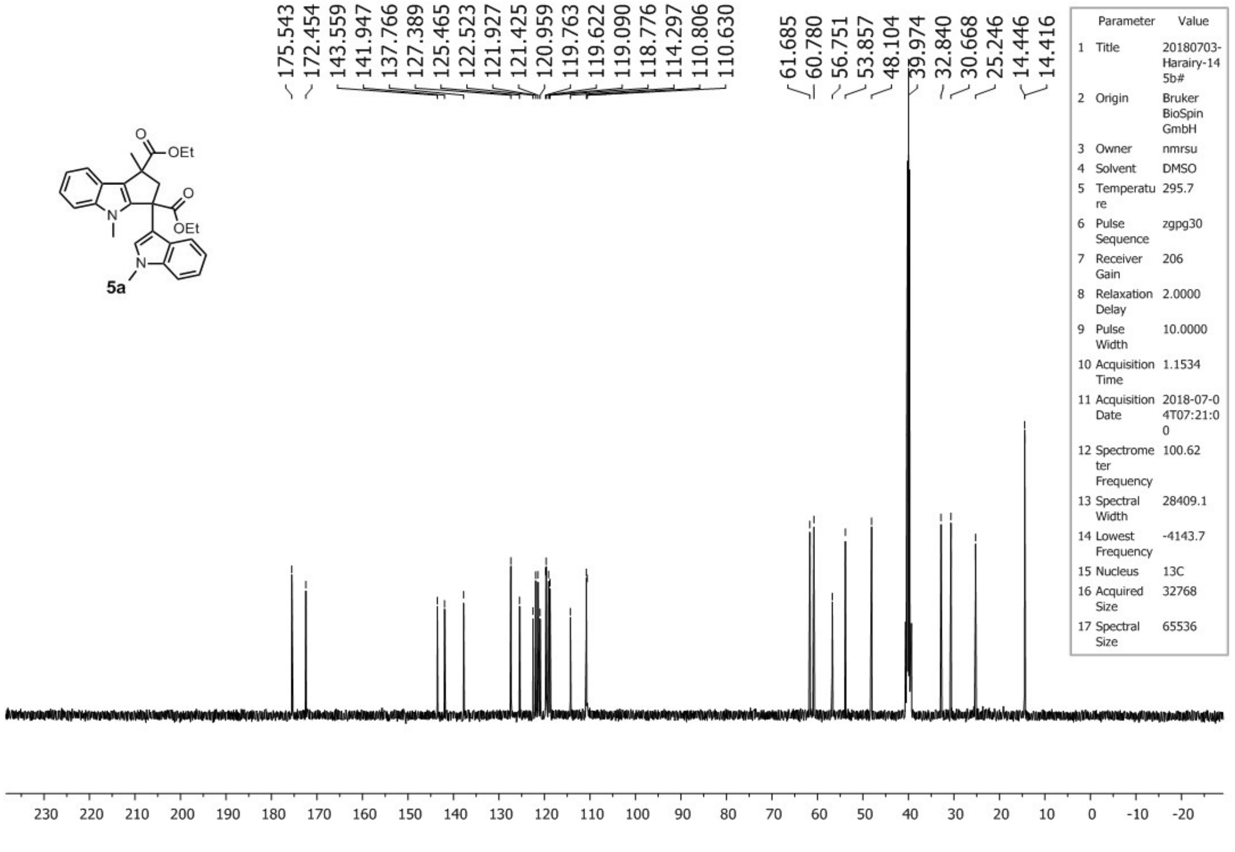
175.543 172.454 143.559 141.947 137.766 127.389 125.465 122.523 121.927 121.425 120.959 119.763 119.622 119.090 118.776 114.297 110.806 110.630
61.685 60.780 56.751 53.857 48.104 39.974 32.840 30.668 25.246 14.446 14.416
OEt
O
OEt
O
N
N
5a
Parameter Value
1 Title 20180703-Harairy-145b#
2 Origin Bruker BioSpin GmbH
3 Owner nmrsu
4 Solvent DMSO
5 Temperature 295.7
6 Pulse Sequence zgpg30
7 Receiver Gain 206
8 Relaxation Delay 2.0000
9 Pulse Width 10.0000
10 Acquisition Time 1.1534
11 Acquisition Date 2018-07-04T07:21:00
12 Spectrometer Frequency 100.62
13 Spectral Width 28409.1
14 Lowest Frequency -4143.7
15 Nucleus 13C
16 Acquired Size 32768
17 Spectral Size 65536
230 220 210 200 190 180 170 160 150 140 130 120 110 100 90 80 70 60 50 40 30 20 10 0 -10 -20
